# Supplementary material for: A multi-omics analysis of advanced triple-negative breast cancer identifies predictors to chemo-immunotherapy and on-treatment dynamics
Source: Cell Rep Med. 2026 Sep 29;7(10):103080. doi: 10.1016/j.xcrm.2026.103080 (PMC13710547; doi:10.1016/j.xcrm.2026.103080)
Supplement: Supplementary file 1 [file mmc1.pdf]

Cell Reports Medicine, Volume *7*

Supplemental information

A multi-omics analysis of advanced triple-negative breast cancer identifies predictors to chemo-immunotherapy and on-treatment dynamics

Nicolaas Van Renne, Elisa Agostinetto, Andrea Joaquín García, Marie Morfouace, Marcin Możejko, Zoë Denis, Sophie Déglise, Bernd Bodenmiller, Henoch S. Hong, David Venet, Marcela Carausu, Robin Liechti, Stéphanie Tissot, Sylvie Rusakiewicz, Mattia Rediti, Xavier Catteau, Daniel Schulz, Delphine Loirat, Daniel Herrero Saboya, Philippe Aftimos, Matteo Benelli, Angel L. Guerrero-Zotano, Andrea Gombos, Hans Wildiers, Jip Van Dinter, Ariane Sickinghe, Nicolas Penel, Jean-Charles Goeminne, Júlio Oliveira, Birute Brasiuniene, Emanuela Romano, Justin M. Balko, Marleen Kok, Ewa Szczurek, Christos Sotiriou, and Laurence Buisseret

Supplemental Information

A multi-omics analysis of advanced Triple-Negative Breast Cancer identifies predictors to chemo-immunotherapy and on-treatment dynamics

Nicolaas Van Renne, Elisa Agostinetto, Andrea Joaquín García, Marie Morfouace, Marcin Możejko, Zoë Denis, Sophie Déglise, Bernd Bodenmiller, Henoch S Hong, David Venet, Marcela Carausu, Robin Liechti, Stéphanie Tissot, Sylvie Rusakiewicz, Mattia Rediti, Xavier Catteau, Daniel Schulz, Delphine Loirat, Daniel Herrero Saboya, Philippe Aftimos, Matteo Benelli, Angel L Guerrero-Zotano, Andrea Gombos, Hans Wildiers, Jip Van Dinter, Ariane Sickinghe, Nicolas Penel, Jean-Charles Goeminne, Júlio Oliveira, Birute Brasiuniene, Emanuela Romano, Justin Balko, Marleen Kok, Ewa Szczurek, Christos Sotiriou and Laurence Buisseret.

Figures S1-S6

Figure S1. Extended characterization of the transcriptomic risk signature in the SYNERGY cohort, related to figures 2 and 3.

Figure S2. Performance of the transcriptomic risk signature across independent metastatic TNBC cohorts, related to figures 2.

Figure S3. TNBC molecular subtypes and clinical outcome across 1st line metastatic TNBC cohorts, related to figure 2.

Figure S4. On-treatment differentially expressed genes from RNA-seq projected on scRNA-seq, related to figure 5.

Figure S5: scRNA-seq landscape of SYNERGY, related to figure 5.

Figure S6: mIF and IMC density analysis, related to figure 6.

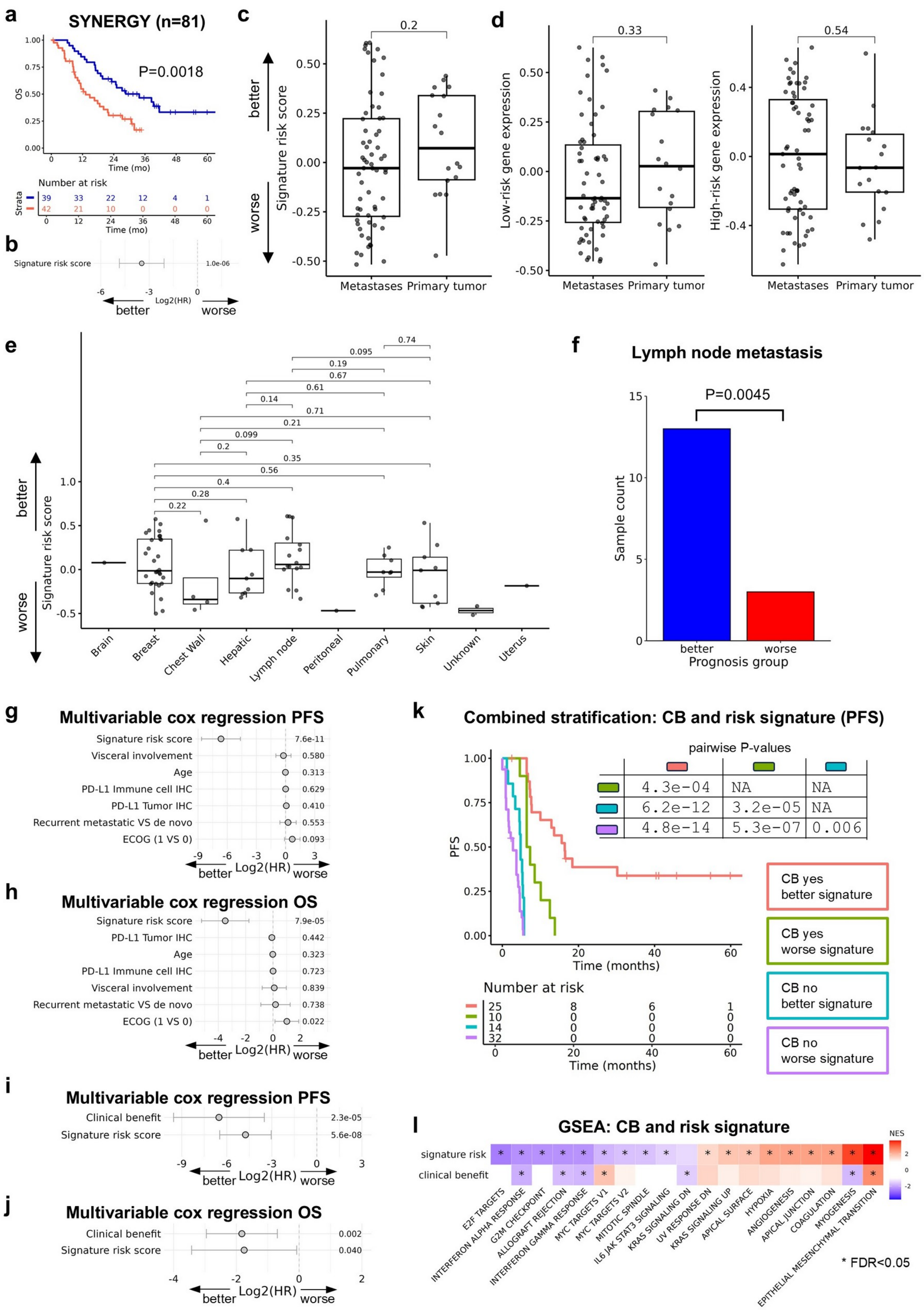
a
SYNERGY (n=81)
P=0.0018
OS
Time (mo)
Number at risk
Strata
39 33 22 12 4 1
42 21 10 0 0 0
b
Signature risk score
1.0e-06
Log2(HR)
better
worse
c
0.2
Signature risk score
better
worse
Metastases
Primary tumor
d
0.33
Low-risk gene expression
0.54
High-risk gene expression
Metastases
Primary tumor
e
Signature risk score
better
worse
Brain
Breast
Chest Wall
Hepatic
Lymph node
Peritoneal
Pulmonary
Skin
Unknown
Uterus
f
Lymph node metastasis
P=0.0045
Sample count
better
worse
Prognosis group
g
Multivariable cox regression PFS
Signature risk score 7.6e-11
Visceral involvement 0.580
Age 0.313
PD-L1 Immune cell IHC 0.629
PD-L1 Tumor IHC 0.410
Recurrent metastatic VS de novo 0.553
ECOG (1 VS 0) 0.093
better Log2(HR) worse
h
Multivariable cox regression OS
Signature risk score 7.9e-05
PD-L1 Tumor IHC 0.442
Age 0.323
PD-L1 Immune cell IHC 0.723
Visceral involvement 0.839
Recurrent metastatic VS de novo 0.738
ECOG (1 VS 0) 0.022
better Log2(HR) worse
i
Multivariable cox regression PFS
Clinical benefit 2.3e-05
Signature risk score 5.6e-08
better Log2(HR) worse
j
Multivariable cox regression OS
Clinical benefit 0.002
Signature risk score 0.040
better Log2(HR) worse
k
Combined stratification: CB and risk signature (PFS)
pairwise P-values
4.3e-04 NA NA
6.2e-12 3.2e-05 NA
4.8e-14 5.3e-07 0.006
PFS
Time (months)
CB yes better signature
CB yes worse signature
CB no better signature
CB no worse signature
Number at risk
25 8 6 1
10 0 0 0
14 0 0 0
32 0 0 0
Time (months)
l
GSEA: CB and risk signature
NES
signature risk
clinical benefit
E2F TARGETS
INTERFERON ALPHA RESPONSE
G2M CHECKPOINT
ALLOGRAFT REJECTION
INTERFERON GAMMA RESPONSE
MYC TARGETS V1
MYC TARGETS V2
MITOTIC SPINDLE
IL6 JAK STAT3 SIGNALING
KRAS SIGNALING DN
UV RESPONSE DN
KRAS SIGNALING UP
APICAL SURFACE
HYPOXIA
ANGIOGENESIS
APICAL JUNCTION
COAGULATION
MYOGENESIS
EPITHELIAL MESENCHYMAL TRANSITION
* FDR<0.05

Figure S1: Extended characterization of the transcriptomic risk signature in the SYNERGY cohort. (a) Kaplan-Meier curves for OS stratified by the transcriptomic risk signature and **(b)** univariable Cox regression analysis showing the association between the continuous signature risk score and OS. **(c)** Signature risk scores and **(d)** GSVA-derived enrichment scores of the high- and low-risk gene sets in primary tumor versus metastatic biopsy samples. P-values indicate two-sided Wilcoxon rank-sum test. **(e)** Distribution of signature risk scores across individual metastatic biopsy sites. P-values indicate two-sided Wilcoxon rank-sum test. **(f)** Distribution of prognosis groups within lymph node metastases. P-value indicates two-sided Fisher's exact test. **(g-h)** Forest plots from multivariable Cox proportional hazards regression analysis including the signature risk score and key clinicopathological variables for PFS and OS. **(i-j)** Multivariable Cox regression including CB status and the signature risk stratification for PFS. **(k)** Kaplan-Meier curves showing joint stratification by CB status and transcriptomic risk signature. **(l)** GSEA showing hallmark pathway enrichment of tumors stratified by the transcriptomic risk signature and CB status. P-values for Kaplan-Meier curves were determined using the two-sided log-rank test. Cox regression P-values indicate two-sided Wald test.

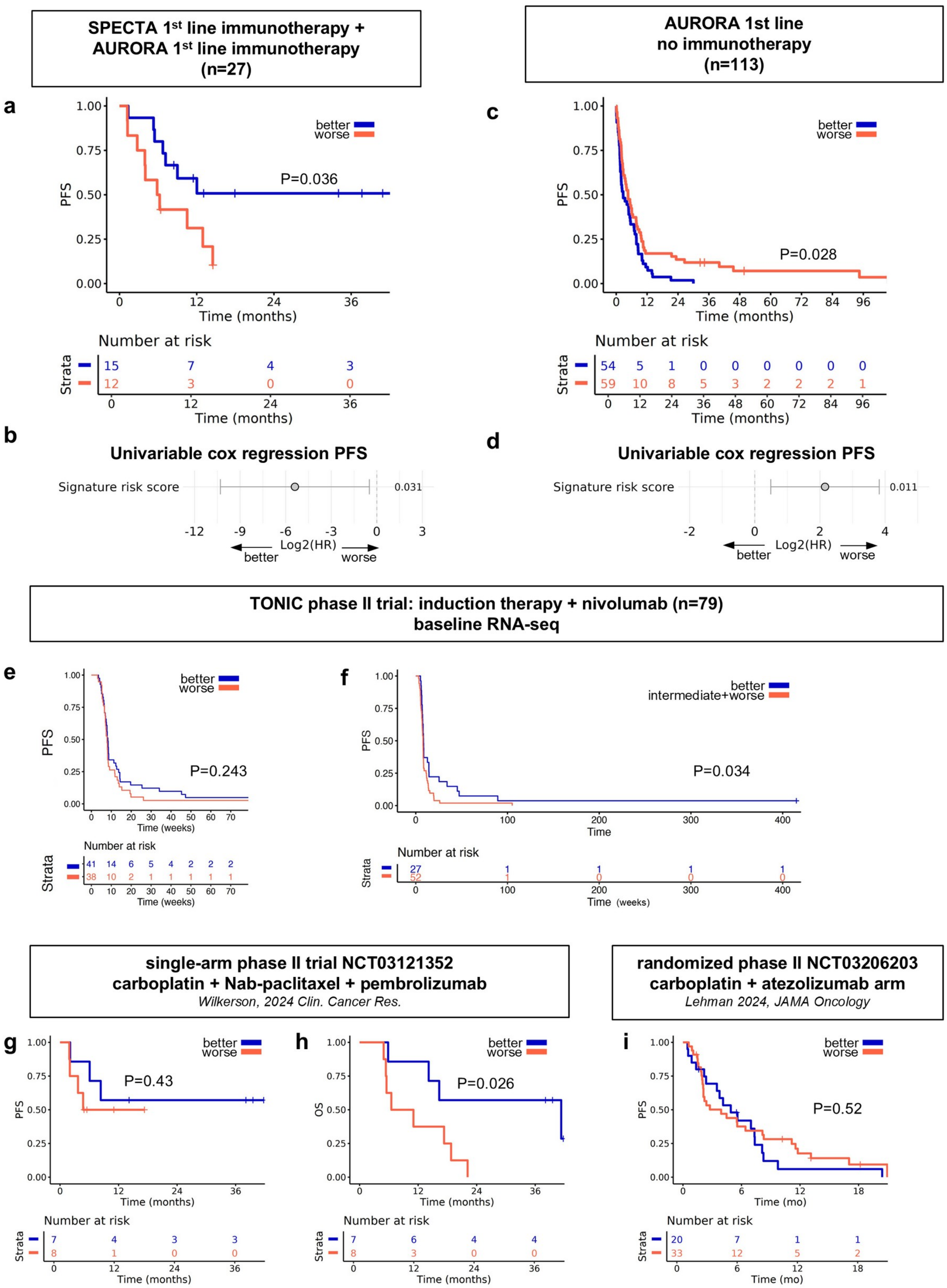
SPECTA 1st line immunotherapy +
AURORA 1st line immunotherapy
(n=27)
AURORA 1st line
no immunotherapy
(n=113)
a
better
worse
P=0.036
PFS
Time (months)
Number at risk
Strata
15 7 4 3
12 3 0 0
Time (months)
c
better
worse
P=0.028
PFS
Time (months)
Number at risk
Strata
54 5 1 0 0 0 0 0 0
59 10 8 5 3 2 2 2 1
Time (months)
b
Univariable cox regression PFS
Signature risk score
0.031
Log2(HR)
better
worse
d
Univariable cox regression PFS
Signature risk score
0.011
Log2(HR)
better
worse
TONIC phase II trial: induction therapy + nivolumab (n=79)
baseline RNA-seq
e
better
worse
PFS
P=0.243
Time (weeks)
Number at risk
Strata
41 14 6 5 4 2 2 2
38 10 2 1 1 1 1 1
Time (weeks)
f
better
intermediate+worse
PFS
P=0.034
Time
Number at risk
Strata
27 1 1 1 1
52 1 0 0 0
Time (weeks)
single-arm phase II trial NCT03121352
carboplatin + Nab-paclitaxel + pembrolizumab
Wilkerson, 2024 Clin. Cancer Res.
randomized phase II NCT03206203
carboplatin + atezolizumab arm
Lehman 2024, JAMA Oncology
g
better
worse
P=0.43
PFS
Time (months)
Number at risk
Strata
7 4 3 3
8 1 0 0
Time (months)
h
better
worse
P=0.026
OS
Time (months)
Number at risk
Strata
7 6 4 4
8 3 0 0
Time (months)
i
better
worse
P=0.52
PFS
Time (mo)
Number at risk
Strata
20 7 1 1
33 12 5 2
Time (mo)

Figure S2: Performance of the transcriptomic risk signature across independent metastatic TNBC cohorts. (a) Kaplan-Meier curves for PFS stratified by the transcriptomic risk signature and **(b)** univariable Cox regression analysis in the combined SPECTA and AURORA first-line chemo-ICI cohort (n=27). **(c-d)** Similarly, for AURORA patients treated with first-line systemic therapy without immunotherapy (n=113). **(e)** Kaplan-Meier curves for PFS stratified by the better-versus-worse transcriptomic risk classification in the TONIC phase II trial (n=79, Kaplan Meier curves truncated at 80 weeks of follow-up) and **(f)** by using a three-group stratification, where samples with low classification confidence (permutation test P>0.05) were assigned to an intermediate group; intermediate and worse-risk groups were combined for comparison. **(g)** Kaplan-Meier curves for PFS and **(h)** OS in the Wilkerson phase II trial (NCT03121352) and **(i)** for PFS in the TBCRC-043 phase II trial (NCT03206203). P-values for Kaplan-Meier curves indicate two-sided log-rank test. Cox regression P-values indicate two-sided Wald test.

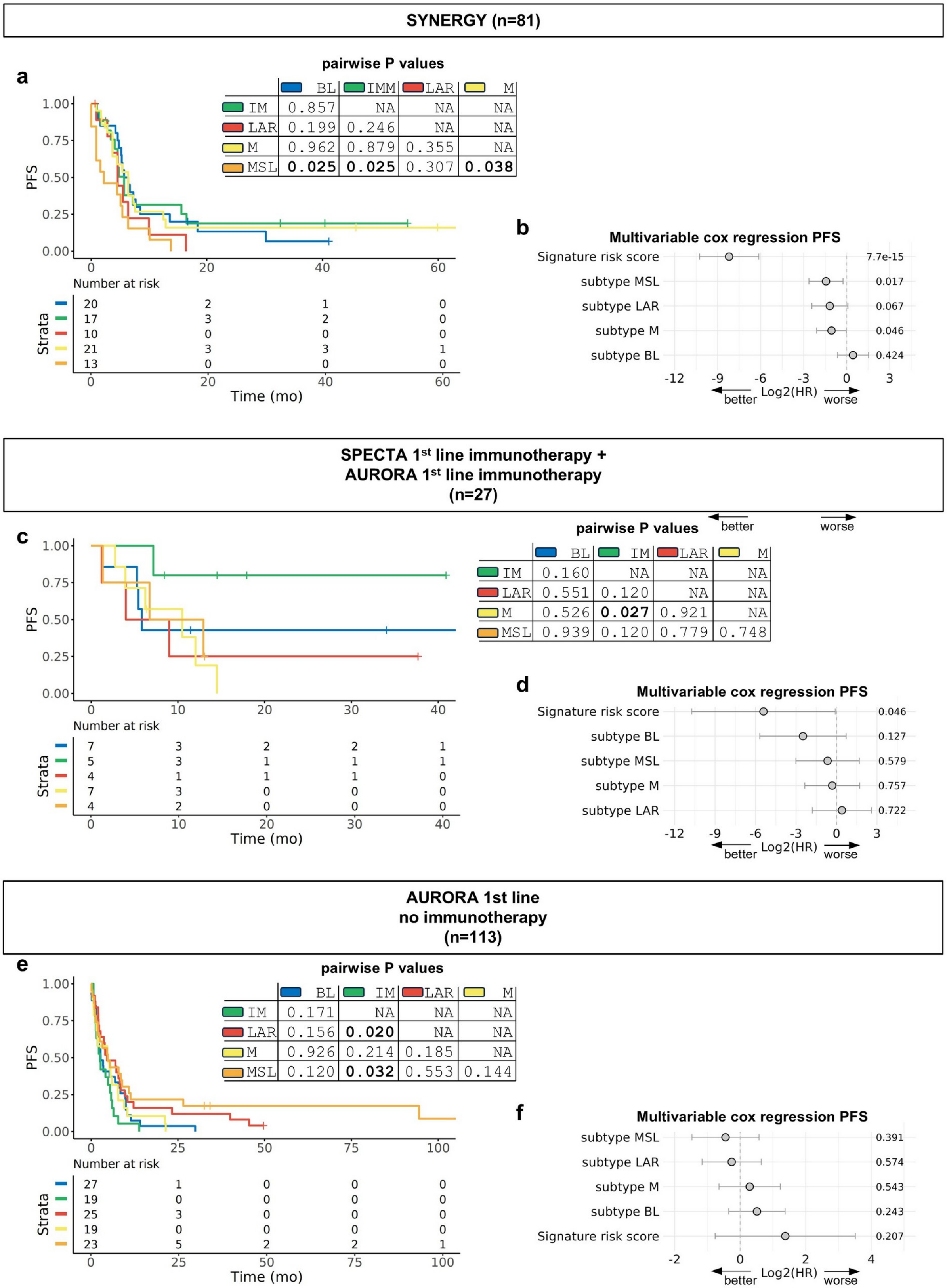

	BL	IMM	LAR	M
IM	0.857	NA	NA	NA
LAR	0.199	0.246	NA	NA
M	0.962	0.879	0.355	NA
MSL	**0.025**	**0.025**	0.307	**0.038**

	BL	IM	LAR	M
IM	0.160	NA	NA	NA
LAR	0.551	0.120	NA	NA
M	0.526	**0.027**	0.921	NA
MSL	0.939	0.120	0.779	0.748

	BL	IM	LAR	M
IM	0.171	NA	NA	NA
LAR	0.156	**0.020**	NA	NA
M	0.926	0.214	0.185	NA
MSL	0.120	**0.032**	0.553	0.144

Figure S3: TNBC molecular subtypes and clinical outcome across 1st line metastatic TNBC cohorts. (a) Kaplan-Meier curves for PFS across molecular subtypes in the SYNERGY cohort. **(b)** Forest plot from multivariable Cox regression including molecular subtype (IM as reference) and the transcriptomic risk score for PFS in SYNERGY. **(c-d)** Similarly for the combined SPECTA and AURORA first-line chemo-ICI cohort (n=27) and **(e-f)** the AURORA patients treated with first-line systemic therapy without immunotherapy (n=113). P-values for Kaplan-Meier curves were determined using the two-sided log-rank test. Cox regression P-values indicate two-sided Wald test.

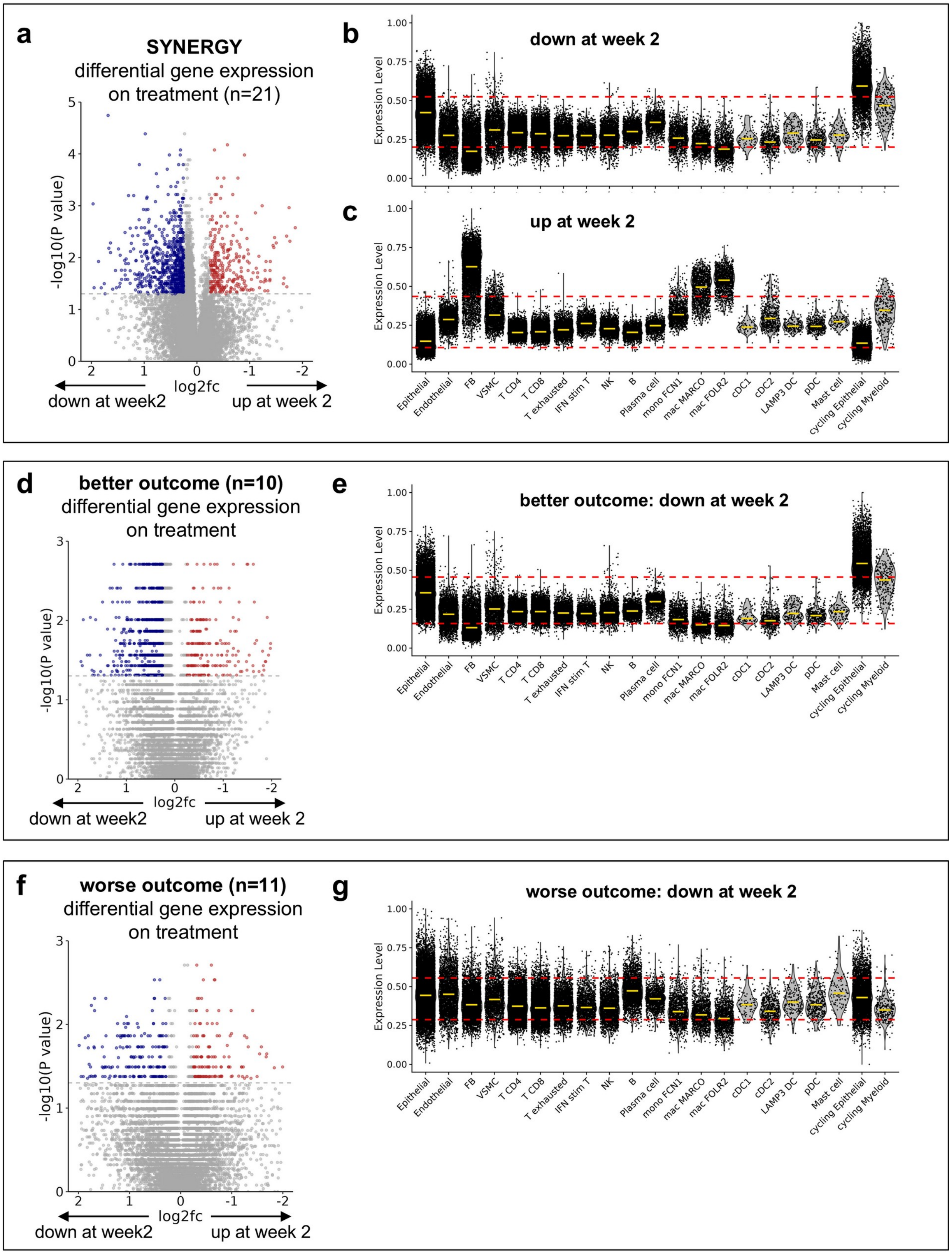
a
SYNERGY
differential gene expression
on treatment (n=21)
-log10(P value)
log2fc
down at week2
up at week 2
b
down at week 2
Expression Level
c
up at week 2
Epithelial
Endothelial
FB
VSMC
T CD4
T CD8
T exhausted
IFN stim T
NK
B
Plasma cell
mono FCN1
mac MARCO
mac FOLR2
cDC1
cDC2
LAMP3 DC
pDC
Mast cell
cycling Epithelial
cycling Myeloid
d
better outcome (n=10)
differential gene expression
on treatment
e
better outcome: down at week 2
f
worse outcome (n=11)
differential gene expression
on treatment
g
worse outcome: down at week 2

Figure S4: On-treatment differentially expressed genes from RNA-seq projected on scRNA-seq. (a) Volcano plot of differentially expressed genes showing bulk RNA-seq genes significantly upregulated (red) and downregulated (blue) on treatment (n=21). Dashed lines indicate 10th and 90th percentile of all module scores. **(b-c)** Scaled module scores of upregulated and downregulated genes are projected on the SYNERGY scRNA-seq landscape. **(d-e)** Similar volcano plots and single-cell projections were generated for the subset of patients with a better prognosis (n=10) and **(f-g)** a worse prognosis (n=11). Volcano plot P values were calculated by two-sided Wilcoxon signed-rank test. Fc: fold change.

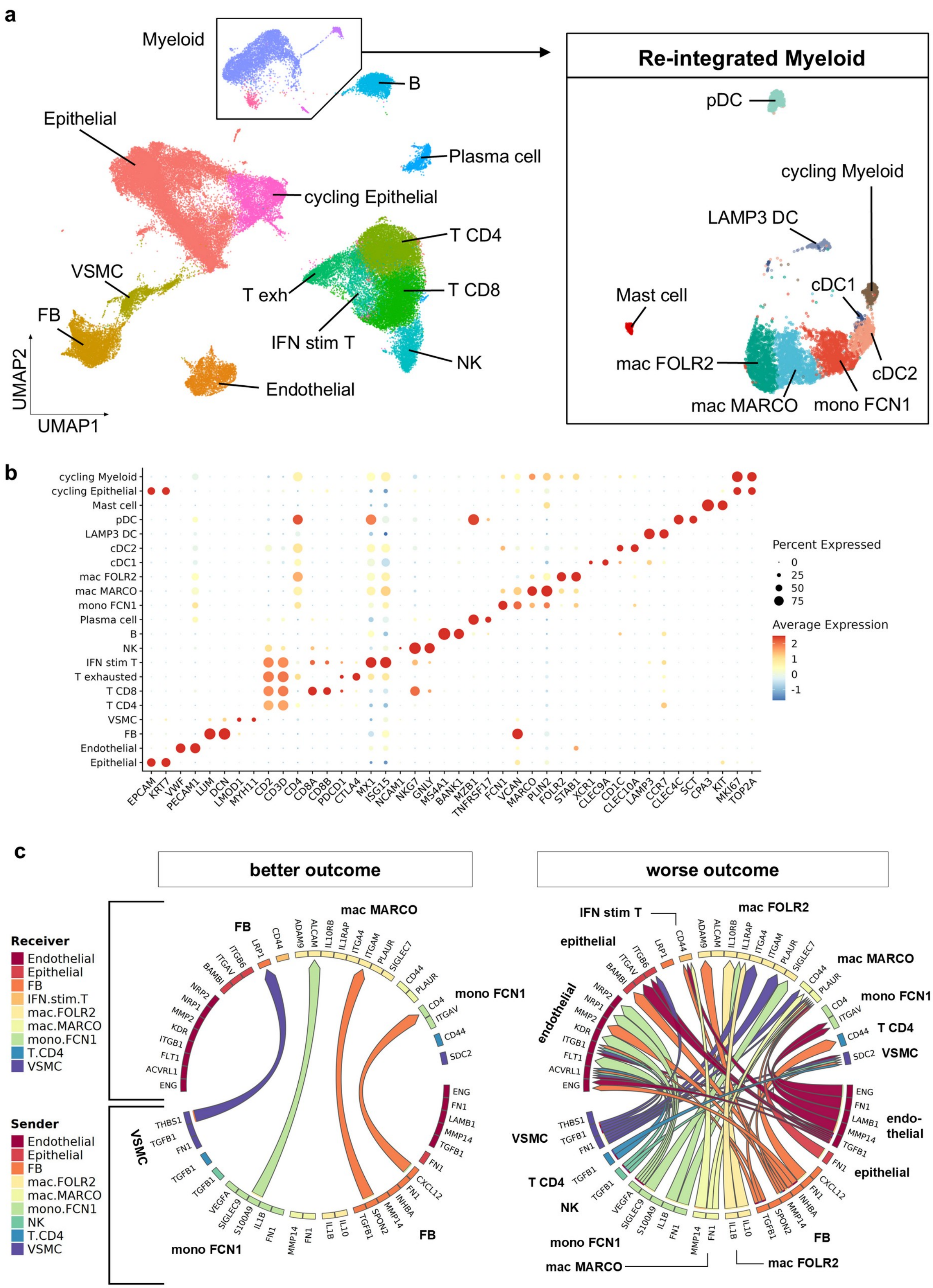
a
Myeloid
B
Plasma cell
Epithelial
cycling Epithelial
T CD4
T CD8
T exh
IFN stim T
NK
VSMC
FB
Endothelial
UMAP1
UMAP2
Re-integrated Myeloid
pDC
cycling Myeloid
LAMP3 DC
cDC1
Mast cell
mac FOLR2
cDC2
mac MARCO
mono FCN1
b
Percent Expressed
Average Expression
c
better outcome
worse outcome
Receiver
Endothelial
Epithelial
FB
IFN.stim.T
mac.FOLR2
mac.MARCO
mono.FCN1
T.CD4
VSMC
Sender
Endothelial
Epithelial
FB
mac.FOLR2
mac.MARCO
mono.FCN1
NK
T.CD4
VSMC

Figure S5: scRNA-seq landscape of SYNERGY. (a) UMAP dimensionality reduction plot with annotated clusters, with the inset showing re-integrated myeloid and granulocyte compartment. **(b)** Dot plots display marker gene expression for each cluster. Dot size indicates the proportions of expressing cells, colored by standardized expression levels. **(c)** MultiNicheNet analysis of cell-cell communication at baseline in better versus worse prognosis patients. Circos plots shows the top 50 differential ligand-receptor interactions between cell types in patients with better outcome (left) and worse outcome (right) at baseline. Arrows indicate directionality from sender to receiver cell type, and arrow color denotes sender cell type. Mac: macrophage. Mono: monocyte. cDC and pDC: conventional and plasmacytoid dendritic cell. IFN stim T: interferon stimulated T cell. Texh: exhausted T cell. VSMC: vascular smooth cell. FB: fibroblast.

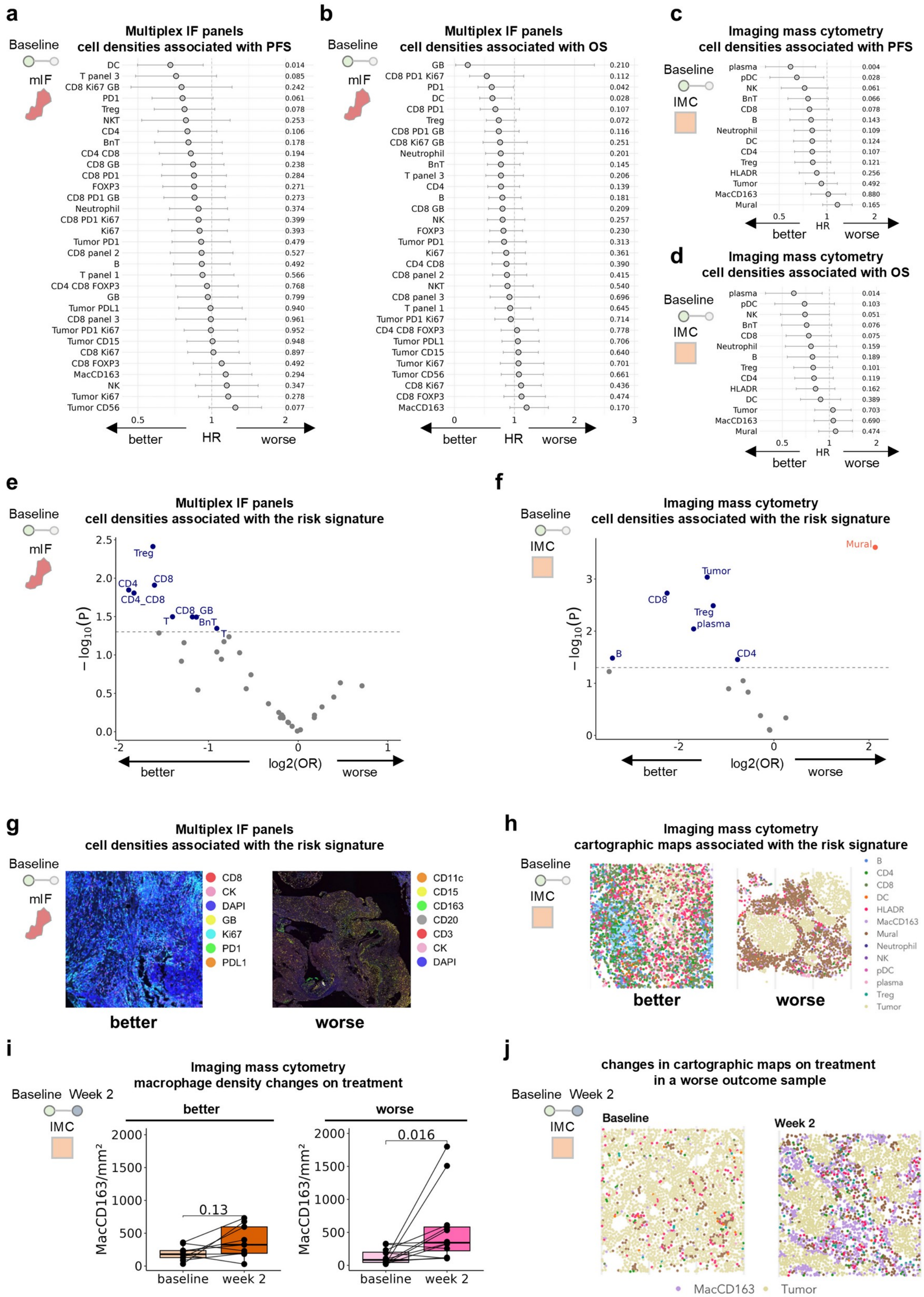

a
Baseline
mIF
Multiplex IF panels
cell densities associated with PFS
DC 0.014
T panel 3 0.085
CD8 Ki67 GB 0.242
PD1 0.061
Treg 0.078
NKT 0.253
CD4 0.106
BnT 0.178
CD4 CD8 0.194
CD8 GB 0.238
CD8 PD1 0.284
FOXP3 0.271
CD8 PD1 GB 0.273
Neutrophil 0.374
CD8 PD1 Ki67 0.399
Ki67 0.393
Tumor PD1 0.479
CD8 panel 2 0.527
B 0.492
T panel 1 0.566
CD4 CD8 FOXP3 0.768
GB 0.799
Tumor PDL1 0.940
CD8 panel 3 0.961
Tumor PD1 Ki67 0.952
Tumor CD15 0.948
CD8 Ki67 0.897
CD8 FOXP3 0.492
MacCD163 0.294
NK 0.347
Tumor Ki67 0.278
Tumor CD56 0.077
0.5
1
2
better
HR
worse
b
Baseline
mIF
Multiplex IF panels
cell densities associated with OS
GB 0.210
CD8 PD1 Ki67 0.112
PD1 0.042
DC 0.028
CD8 PD1 0.107
Treg 0.072
CD8 PD1 GB 0.116
CD8 Ki67 GB 0.251
Neutrophil 0.201
BnT 0.145
T panel 3 0.206
CD4 0.139
B 0.181
CD8 GB 0.209
NK 0.257
FOXP3 0.230
Tumor PD1 0.313
Ki67 0.361
CD4 CD8 0.390
CD8 panel 2 0.415
NKT 0.540
CD8 panel 3 0.696
T panel 1 0.645
Tumor PD1 Ki67 0.714
CD4 CD8 FOXP3 0.778
Tumor PDL1 0.706
Tumor CD15 0.640
Tumor Ki67 0.701
Tumor CD56 0.661
CD8 Ki67 0.436
CD8 FOXP3 0.474
MacCD163 0.170
0
1
2
3
better
HR
worse
c
Baseline
IMC
Imaging mass cytometry
cell densities associated with PFS
plasma 0.004
pDC 0.028
NK 0.061
BnT 0.066
CD8 0.078
B 0.143
Neutrophil 0.109
DC 0.124
CD4 0.107
Treg 0.121
HLADR 0.256
Tumor 0.492
MacCD163 0.880
Mural 0.165
0.5
1
2
better
HR
worse
d
Baseline
IMC
Imaging mass cytometry
cell densities associated with OS
plasma 0.014
pDC 0.103
NK 0.051
BnT 0.076
CD8 0.075
Neutrophil 0.159
B 0.189
Treg 0.101
CD4 0.119
HLADR 0.162
DC 0.389
Tumor 0.703
MacCD163 0.690
Mural 0.474
0.5
1
2
better
HR
worse
e
Baseline
mIF
Multiplex IF panels
cell densities associated with the risk signature
Treg
CD8
CD4
CD4_CD8
CD8_GB
T
BnT
T
−log10(P)
log2(OR)
better
worse
f
Baseline
IMC
Imaging mass cytometry
cell densities associated with the risk signature
Mural
Tumor
CD8
Treg
plasma
B
CD4
−log10(P)
log2(OR)
better
worse
g
Baseline
mIF
Multiplex IF panels
cell densities associated with the risk signature
CD8
CK
DAPI
GB
Ki67
PD1
PDL1
CD11c
CD15
CD163
CD20
CD3
CK
DAPI
better
worse
h
Baseline
IMC
Imaging mass cytometry
cartographic maps associated with the risk signature
B
CD4
CD8
DC
HLADR
MacCD163
Mural
Neutrophil
NK
pDC
plasma
Treg
Tumor
better
worse
i
Baseline Week 2
IMC
Imaging mass cytometry
macrophage density changes on treatment
better
worse
MacCD163/mm²
0.13
0.016
baseline
week 2
j
Baseline Week 2
IMC
changes in cartographic maps on treatment
in a worse outcome sample
Baseline
Week 2
MacCD163
Tumor

Figure S6: mIF and IMC density analysis (a-b) Cox proportional hazards regression analyses for overall survival (OS) and progression-free survival (PFS) across mIF-derived and **(c-d)** IMC-derived cell-type densities. HR: hazard ratio; whiskers show 95% confidence interval. P-values indicate two-sided Wald test. **(e)** Volcano plots showing associations between mIF- and **(f)** IMC-derived cell-type densities and patient outcome according to the risk signature. The x-axis represents the log_2 odds ratio (OR) from logistic regression models for each cell type's association with worse outcome (positive values) or better outcome (negative values) patients. P values indicate two-sided Wald test. GB: granzyme B^+ T cell, pDC plasmacytoid dendritic cell, BnT: B or T cell in close proximity, HLADR: undefined HLA-DR^+ antigen presenting cell, Plasma: plasma cell. **(g)** Example miF panels and **(h)** IMC cartographic maps of patients with a differential clinical outcome. **(i)** Boxplots showing differences in macrophage levels between baseline and week 2 on-treatment. P values indicate Wilcoxon signed-rank test. **(j)** Representative worse outcome sample at baseline and week 2, illustrating increased macrophage levels on-treatment.
