# Supplementary material for: A multi-omics analysis of advanced triple-negative breast cancer identifies predictors to chemo-immunotherapy and on-treatment dynamics
Source: Cell Rep Med. 2026 Sep 29;7(10):103080. doi: 10.1016/j.xcrm.2026.103080 (PMC13710547; doi:10.1016/j.xcrm.2026.103080)
Supplement: Supplementary file 2 [file mmc8.pdf]

Cell Reports
Medicine

Article

A multi-omics analysis of advanced triple-negative breast cancer identifies predictors to chemo-immunotherapy and on-treatment dynamics

Graphical abstract

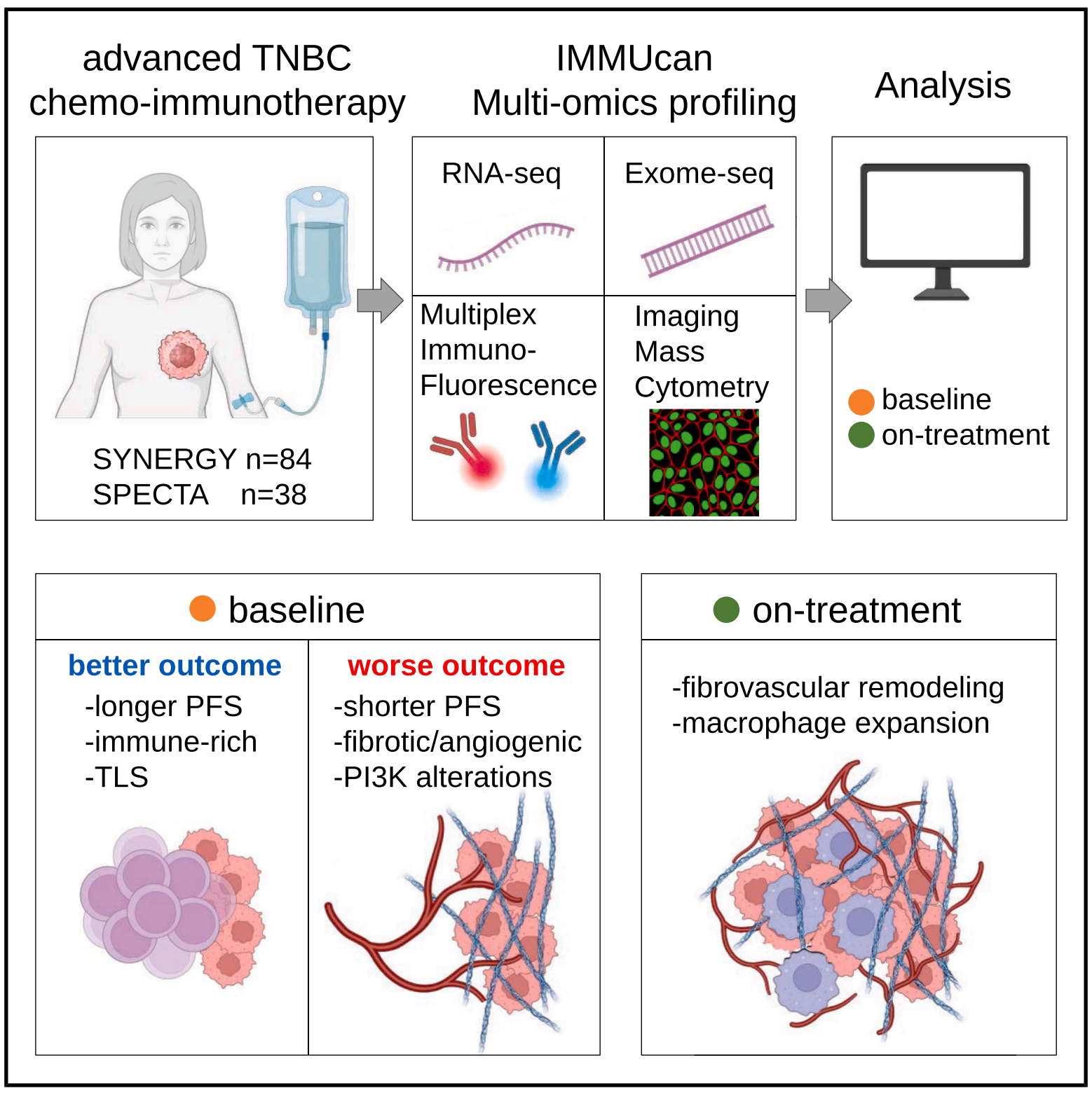

Authors

Nicolaas Van Renne, Elisa Agostinetto, Andrea Joaquín García, ..., Ewa Szczurek, Christos Sotiriou, Laurence Buisseret

Correspondence

laurence.buisseret@bordet.be

In brief

Van Renne, Agostinetto, et al. perform a multi-omics characterization of advanced TNBC to identify response predictors and treatment-induced dynamics during chemo-ICI. Poor outcomes are associated with fibrovascular and myeloid remodeling, highlighting coordinated resistance mechanisms, actionable therapeutic targets, and the value of microenvironment-based stratification for future ICI-based trials.

Highlights

- A transcriptomic signature stratifies chemo-immunotherapy outcomes in TNBC
- Immune-rich and fibrovascular TMEs associate with distinct clinical outcomes
- Resistance to ICI involves fibrovascular remodeling and immune reprogramming
- Our findings support biologically-guided ICI trials and new therapeutic targets

Van Renne et al., 2026, Cell Reports Medicine 7, 103080
October 20, 2026 © 2026 The Authors. Published by Elsevier Inc.
https://doi.org/10.1016/j.xcrm.2026.103080

CellPress

Cell Reports Medicine

CellPress
OPEN ACCESS

Article

A multi-omics analysis of advanced triple-negative breast cancer identifies predictors to chemo-immunotherapy and on-treatment dynamics

Nicolaas Van Renne,[1,28] Elisa Agostinetto,[1,2,28] Andrea Joaquín García,[1] Marie Morfouace,[3,4] Marcin Możejko,[5] Zoë Denis,[1] Sophie Déglise,[6,7] Bernd Bodenmiller,[6,7] Henoch S. Hong,[8] David Venet,[1] Marcela Carausu,[1] Robin Liechti,[9] Stéphanie Tissot,[10] Sylvie Rusakiewicz,[10] Mattia Rediti,[1,11] Xavier Catteau,[2] Daniel Schulz,[6,7] Delphine Loirat,[12] Daniel Herrero Saboya,[13,14] Philippe Aftimos,[2] Matteo Benelli,[15]

(Author list continued on next page)

[1]Breast Cancer Translational Research Laboratory J-C Heuson (BCTL), Institut Jules Bordet, Université Libre de Bruxelles (ULB), Bruxelles, Belgium
[2]Hôpital Universitaire de Bruxelles (H.U.B), Institut Jules Bordet, Université Libre de Bruxelles (ULB), Brussels, Belgium
[3]EORTC Headquarters, Brussels, Belgium
[4]Institut Gustave Roussy, Villejuif, France
[5]Faculty of Mathematics, Informatics and Mechanics, University of Warsaw, Warsaw, Poland
[6]Department of Quantitative Biomedicine, University of Zurich, Zurich, Switzerland
[7]Institute of Molecular Health Sciences, ETH Zurich, Zurich, Switzerland
[8]The Healthcare Business of Merck KGaA, Darmstadt, Germany
[9]Vital-IT Group, SIB Swiss Institute of Bioinformatics, Lausanne, Switzerland
[10]Center of Experimental Therapeutics, CHUV, Lausanne, Switzerland
[11]IFOM ETS, the AIRC Institute of Molecular Oncology, Milan, Italy
[12]Medical Oncology Department, Institut Curie, Paris, France
[13]Computational and Systems Biology of Cancer, Institut Curie, INSERM U1331, Mines ParisTech, PSL University, Paris, France
[14]Computational Medicine, Servier Research & Development, Saclay, France
[15]Department of Experimental and Clinical Biomedical Sciences, University of Florence, Florence, Italy
[16]Medical Oncology Department, Instituto Valenciano de Oncologia, Valencia, Spain
[17]Grupo Español de Investigación Cáncer de Mama (GEICAM), San Sebastián de los Reyes, Spain
[18]Department of General Medical Oncology, University Hospitals Leuven, Leuven, Belgium

(Affiliations continued on next page)

SUMMARY

No validated biomarkers beyond PD-L1 currently guide immune checkpoint inhibition (ICI) in advanced triple-negative breast cancer (TNBC), and how tumors remodel their microenvironment in response to ICI remains incompletely understood. We perform a temporal analysis of patients with advanced TNBC treated with chemo-immunotherapy in the SYNERGY trial (NCT03616886; $n = 84$) and the EORTC-1553-SPECTA initiative (NCT02834884; $n = 38$) using RNA sequencing, whole-exome sequencing, multiplex immunofluorescence, and imaging mass cytometry. A transcriptomic progression-free survival (PFS)-based risk signature stratifies outcomes and is validated across ICI-treated cohorts, while showing context-dependent behavior in non-ICI settings. Favorable outcomes are associated with immune-enriched tumors with organized lymphoid architecture, while poor outcomes are linked to fibrotic, angiogenic microenvironments with frequent PI3K alterations. Upon treatment, early progression was characterized by fibrovascular remodeling, macrophage expansion, and upregulation of alternative immunosuppressive mediators. These findings establish fibrovascular and myeloid reprogramming as a coordinated resistance program, identify actionable therapeutic targets, and support microenvironment stratification in future ICI-based trials.

INTRODUCTION

The advent of immune checkpoint inhibition (ICI) has marked a paradigm shift in oncology. In locally advanced or metastatic triple-negative breast cancer (TNBC), combining chemotherapy with ICI is now the standard of care (SOC) for patients with PD-L1-positive tumors.[1] Although this has led to meaningful progress in patient outcomes, not all patients derive durable benefit.[2]

Currently, PD-L1 expression by immunohistochemistry (IHC) is the only approved biomarker to guide immunotherapy

This is an open access article under the CC BY license (http://creativecommons.org/licenses/by/4.0/).

Angel L. Guerrero-Zotano,[16,17] Andrea Gombos,[2] Hans Wildiers,[18] Jip Van Dinter,[19] Ariane Sickinghe,[19,20] Nicolas Penel,[21] Jean-Charles Goeminne,[22] Júlio Oliveira,[23] Birute Brasiuniene,[24,25] Emanuela Romano,[12] Justin M. Balko,[26] Marleen Kok,[19,20] Ewa Szczurek,[5,27] Christos Sotiriou,[1,2,29] and Laurence Buisseret[1,2,29,30,*]
[19]Division of Tumor Biology and Immunology, The Netherlands Cancer Institute, Amsterdam, the Netherlands
[20]Division of Medical Oncology, The Netherlands Cancer Institute, Amsterdam, the Netherlands
[21]Centre Oscar Lambret, Lille, France
[22]Centre Hospitalier Universitaire UCL Namur (site Sainte Elisabeth), Namur, Belgium
[23]Instituto Português de Oncologia do Porto, Porto, Portugal
[24]National Cancer Center of Vilnius University Hospital Santaros clinics, Vilnius, Lithuania
[25]Faculty of Medicine, Vilnius University, Vilnius, Lithuania
[26]Department of Medicine, Vanderbilt University Medical Center, Nashville, TN, USA
[27]Institute of AI for Health, Helmholtz Munich, Neuherberg, Germany
[28]These authors contributed equally
[29]These authors contributed equally
[30]Lead contact
*Correspondence: laurence.buisseret@bordet.be
https://doi.org/10.1016/j.xcrm.2026.103080

selection in advanced TNBC. However, its predictive value is limited, and there is a need for more effective predictive biomarkers.[3] The intrinsic heterogeneity of TNBC further complicates biomarker discovery. Molecular subtyping has emerged as a powerful approach to capture this diversity,[4–8] and more recently, subtype-guided trials such as the FUTURE[9–11] program have shown their therapeutic relevance in metastatic TNBC. Nevertheless, the relationship between subtype and chemo-immunotherapy response in advanced TNBC cohorts remains incompletely defined.

To identify prognostic biomarkers and resolve how molecular subtypes shape ICI response, we performed comprehensive multi-omics profiling of advanced TNBC from two patient cohorts treated with ICI, either in the context of a clinical trial or as SOC. These data were generated within the IMMUcan consortium (https://immucan.eu/), a European immuno-oncology profiling initiative that integrates genomic, transcriptomic, proteomic, and multiplex protein imaging modalities with longitudinal clinical information.

RESULTS

Patient population and multimodal characterization

The IMMUcan advanced TNBC cohort (BC1 cohort, n = 122) included patients from the phase 1/2 SYNERGY trial (NCT03616886, n = 84) and the EORTC-1553-SPECTA study (NCT02834884, n = 38) (Figure 1A). Tumor biopsies underwent comprehensive molecular and immune profiling, including bulk RNA sequencing (RNA-seq), whole-exome sequencing (WES), multiplex immunofluorescence (mIF), and imaging mass cytometry (IMC) (Figure 1B). Additionally, for the SYNERGY trial, histological characterization and IHC data of the full cohort[12] and single cell RNA-seq (scRNA-seq) profiling of 27 tumors have been previously reported.[13] Briefly, the SYNERGY trial is an investigator-initiated, randomized study designed to assess whether inhibiting the immunosuppressive adenosine pathway through the anti-CD73 antibody oleclumab could enhance the antitumor effects of chemo-immunotherapy in advanced TNBC. Patients were randomized to receive weekly carboplatin and paclitaxel for 12 cycles in combination with the anti-PD-L1 antibody durvalumab with (arm A) or without (arm B) oleclumab (Figure 1C). The primary endpoint was the clinical benefit (CB) rate at 24 weeks, defined as stable disease or partial or complete response per RECIST v.1.1. Secondary endpoints included progression-free survival (PFS) and overall survival (OS). The primary endpoint of SYNERGY was previously reported,[12] showing no significant difference between the two treatment arms. PFS and OS were subsequently presented, also showing no difference between the treatment arms.[14] In SYNERGY, baseline tumor biopsies (metastasis: n = 66, and primary tumors: n = 18) (Figure 1D) and on-treatment biopsies at week 2 (n = 21) were obtained.

Patients from the EORTC-1553-SPECTA study received first-line treatment according to the SOC, with 22 of 38 receiving chemo-immunotherapy and the other 16 receiving chemotherapy alone. Tumor biopsies (metastasis; n = 5 and primary tumors; n = 33) (Figure 1D) and on-treatment biopsies (n = 3) were similarly collected. An overview of patient characteristics and all data types is provided for SYNERGY (Figures 1E; Table S1) and SPECTA (Table S2).

Baseline tumor transcriptomes are associated with clinical outcome

To identify prognostic biomarkers, we aimed to define a transcriptomic signature associated with PFS using a previously described machine learning approach.[15,16] PFS was chosen over the categorical CB endpoint as it captures both the depth and duration of the response and provides a standardized endpoint for consistent evaluation across cohorts. After excluding patients with missing RNA-seq data, absence of ICI treatment, or uncurated clinical data, baseline samples from the SYNERGY cohort (n = 81) were used to train the model, while the independent SPECTA cohort (n = 17) was used for validation. Briefly, for every gene, a Cox score was computed to assess the association between the gene's expression level and PFS. Only genes that consistently met the significance threshold ($p < 0.05$) across leave-one-sample-out cross-iterations were retained, resulting in a set of 271 genes (Table S3). Among these, 119 genes with a positive Cox score were associated with disease progression and classified as "high-risk genes." In comparison, 152 genes with a negative Cox score were inversely associated

CellPress
OPEN ACCESS

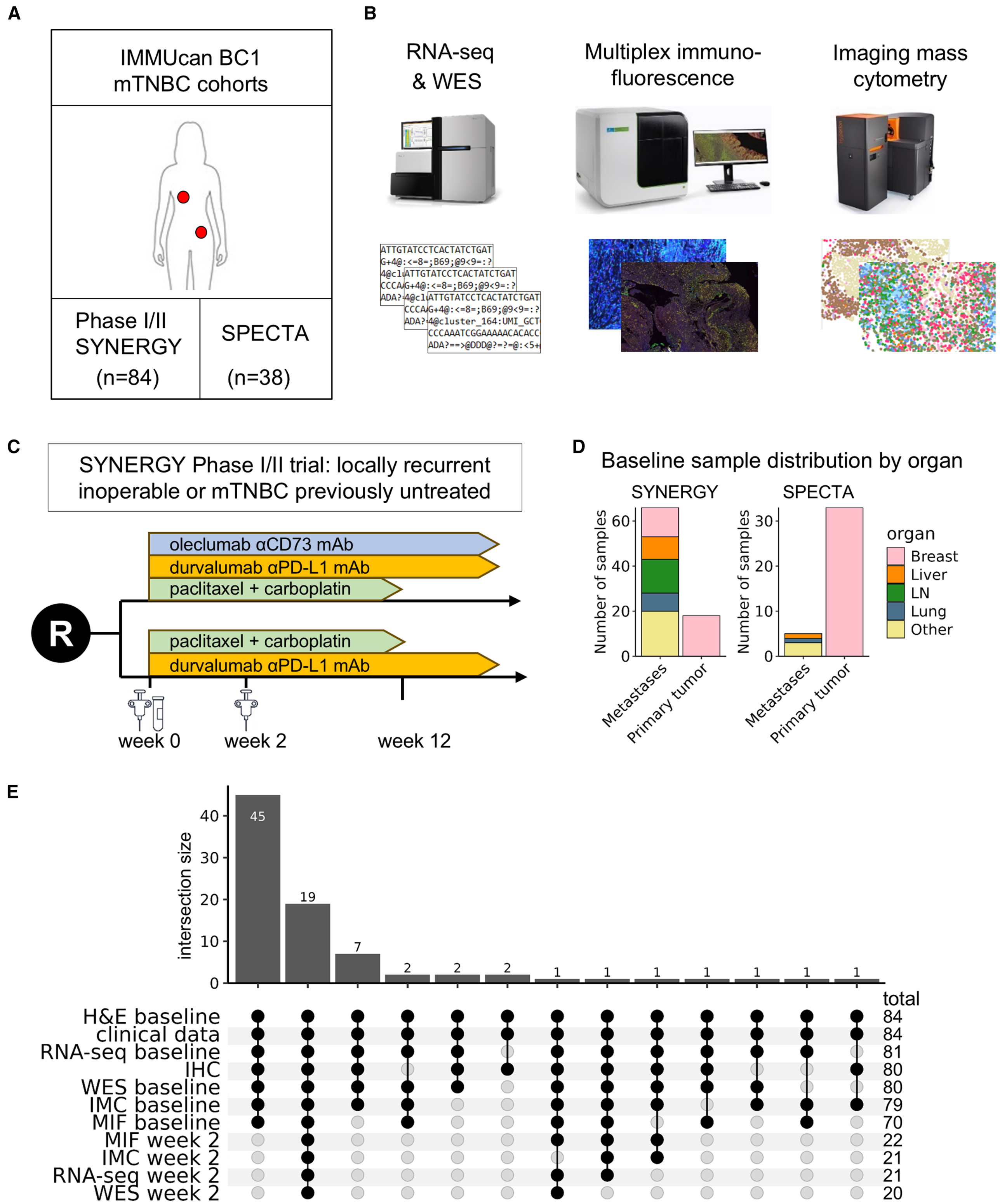

Figure 1. Overview of IMMUcan BC1

(A–C) (A) Patient cohorts, (B) applied technologies, and (C) SYNERGY trial design.

(D) Tissue origin of samples collected for BC1.

(E) Upset plot showing data modalities across BC1 SYNERGY. mTNBC, metastatic TNBC.

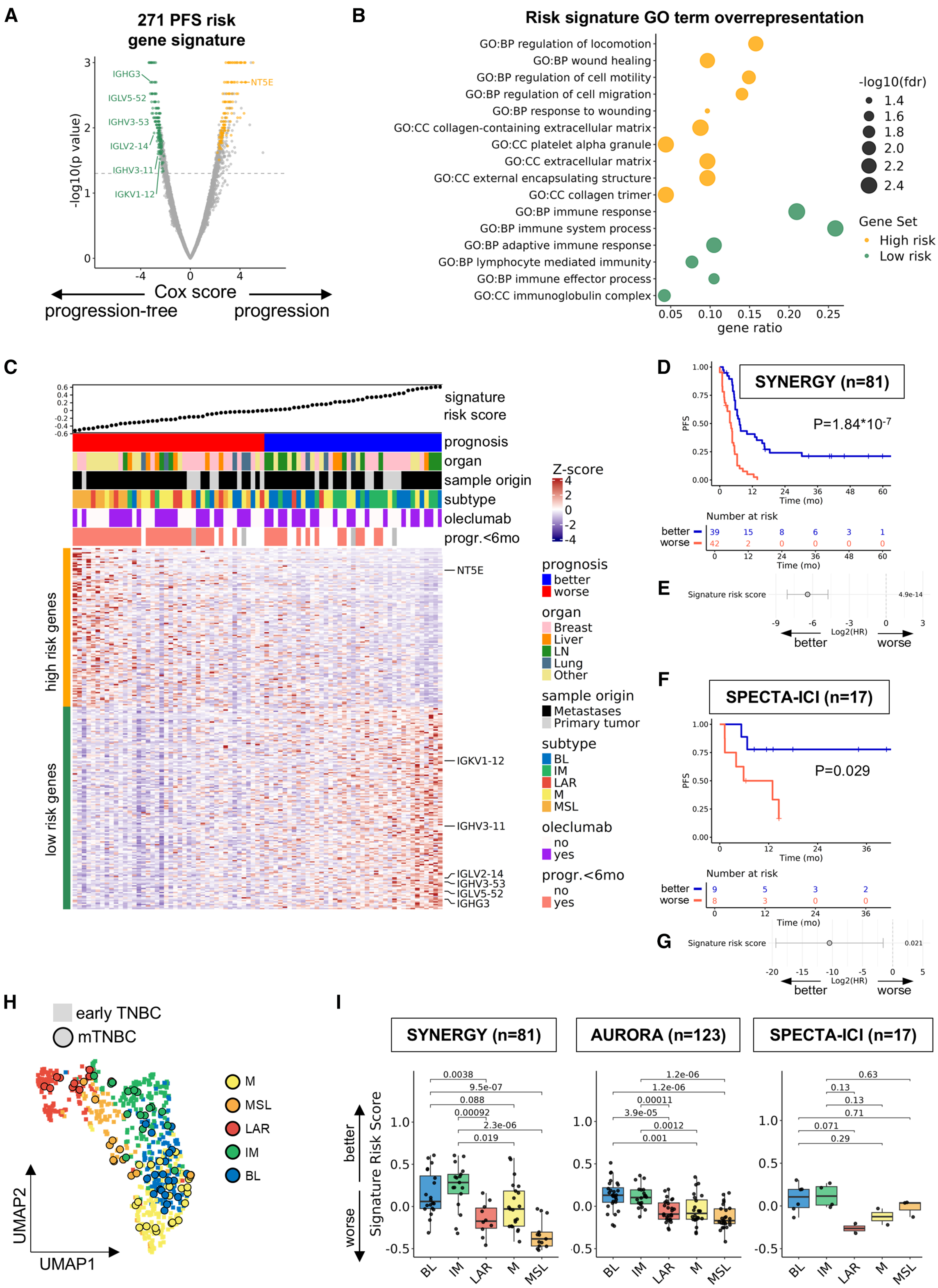

Figure 2. A 271-gene signature predicts PFS

(A) Volcano plot of risk genes. *p* values indicate two-sided random permutation test. The dashed line indicates the $p = 0.05$ cutoff in the leave-one-sample-out iteration.

(legend continued on next page)

CellPress
OPEN ACCESS

with disease progression and classified as "low-risk genes" (Figure 2A). In this signature, low-risk genes were mainly involved in immune response functions, with a striking presence of B cell receptor genes. In contrast, high-risk genes were mainly related to fibrosis or wound healing (Figures 2B; Table S3). Notably, the high-risk genes included *NT5E*, encoding CD73,[17] which is the target of oleclumab, tested in combination with chemo-ICI in the SYNERGY trial.

A signature risk score was subsequently calculated for each sample using a class prediction algorithm,[18] dividing the cohort into two prognostic groups based on the sign of the signature risk score (Figure 2C). Patients assigned to the "better outcome" group (i.e., positive signature score) exhibited a significantly longer PFS (Figure 2D) and OS (Figure S1A) compared to those assigned to the "worse outcome" group. Consistently, in univariable Cox regression analysis, the continuous signature risk score was significantly associated with PFS (Figure 2E) and OS (Figure S1B), showing a protective effect for higher scores. Since *NT5E* (CD73) was among the high-risk genes, we assessed whether patients in the worse-outcome group benefited from CD73 blockade with oleclumab. However, no significant difference in PFS or OS was observed (data not shown).

As the SYNERGY baseline samples comprise both primary tumor and metastatic biopsies, we examined whether the transcriptomic signature differed between these two sample types. No statistically significant differences were observed, neither in signature risk scores nor in gene set variation analysis (GSVA)-derived enrichment scores of the high- and low-risk gene sets (Figures S1C and S1D). However, metastatic samples tended to display lower (i.e., worse) risk scores and reduced expression of low-risk genes. Indeed, metastatic lesions typically harbor reduced immune cell infiltration,[19] which is expected to result in lower expression of the immune-related transcripts that constitute the low-risk gene set. In line with this, when risk scores were compared across individual metastatic biopsy sites, no significant differences were observed overall (Figure S1E), though lymph node metastases, given their intrinsic immune-rich microenvironment, displayed a significant enrichment of better-prognosis tumors (13 of 16; $p = 0.0045$) (Figure S1F).

To ensure the signature constitutes an independent prognostic factor, we performed multivariable Cox regression combining the signature risk score with key clinicopathological variables. The signature remained strongly and independently associated with both PFS and OS (Figures S1G and S1H) and likewise captured prognostic information independent of CB (Figures S1I and S1J). When jointly stratified by CB status and transcriptomic risk, the signature further refined outcome prediction within the group with CB, suggesting that our signature could be helpful to identify patients who will have a very durable response to chemo-immunotherapy (Figure S1K).

Next, we validated the signature in the independent SPECTA cohort where low-risk patients showed significantly longer PFS ($p = 0.029$) (Figure 2F). Univariable Cox regression analysis confirmed the association between the continuous risk score and PFS (Figure 2G).

To further assess the performance of the signature, we leveraged data from the AURORA program,[20,21] a large international molecular profiling initiative of metastatic breast cancer including RNA-seq data linked with curated clinical annotations from more than 1,150 patients recruited across 11 European countries. Within this database, 123 patients with first-line advanced TNBC were identified. Among these, 10 patients had received first-line chemo-ICI and were combined with the SPECTA cohort to generate an expanded "real-world" validation set ($n = 27$) matched to the clinical context of our study. In this combined cohort, the transcriptomic signature confirmed its prognostic value for PFS (Figures S2A and S2B).

Interestingly, when applying the signature to AURORA TNBC patients treated with first-line systemic therapy without ICI ($n = 113$), the direction of association was reversed, with better-risk tumors showing inferior PFS compared to worse-risk tumors (Figures S2C and S2D). These findings suggest that the transcriptomic signature captures biological features specifically associated with benefit from chemo-ICI, rather than reflecting a general prognostic signal independent of the treatment context.

We further evaluated the signature in the TONIC trial, a phase 2 study of nivolumab following different induction strategies in heavily pretreated metastatic TNBC,[22] representing a biologically distinct context compared to first-line chemo-ICI. While the predefined binary better-versus-worse classification showed only a trend toward outcome separation without reaching statistical significance (Figure S2E), we additionally incorporated classification confidence into the analysis by assigning samples with low-confidence predictions (permutation test $p > 0.05$) to an intermediate group.[16] Under this confidence-aware stratification, combining intermediate and worse-risk tumors revealed significant separation from the better-risk group ($p = 0.034$) (Figure S2F), suggesting that the signature retains the ability to identify a subgroup of patients with more favorable outcomes even in this later-line setting.

Finally, we applied the signature to two independent publicly available clinical trial datasets. First, in a prospective single-arm phase 2 trial of carboplatin, nab-paclitaxel, and pembrolizumab in patients with metastatic TNBC with up to two prior lines of therapy (NCT03121352),[23] the transcriptomic risk signature was significantly associated with OS despite the limited sample size

(B) Ontology enrichment of risk genes. Gene ratio indicates proportion annotated to each term.
(C) Heatmap shows risk gene expression in SYNERGY. Progr.<6 months, progression in <6 months. Gray indicates patients with <6 months' follow-up.
(D–G) (D) PFS curves among risk-stratified groups and (E) univariable Cox regression of signature risk score for PFS in SYNERGY and (F and G) SPECTA. *p* values for Kaplan-Meier curves indicate two-sided log rank test. HR, hazard ratio. Two-sided Wald test for Cox regression is indicated to the right of the 95% confidence interval.
(H) UMAP of RNA-seq of SCAN-B (early TNBC) and SYNERGY by subtype.
(I) Distribution of signature risk scores across subtypes in first-line metastatic cohorts. Box plots indicate the range (central 50% of the data) with the central line indicating the median. Whiskers extend to 1.5× the interquartile range. Pairwise *p* values indicate two-sided Wilcoxon rank-sum test.

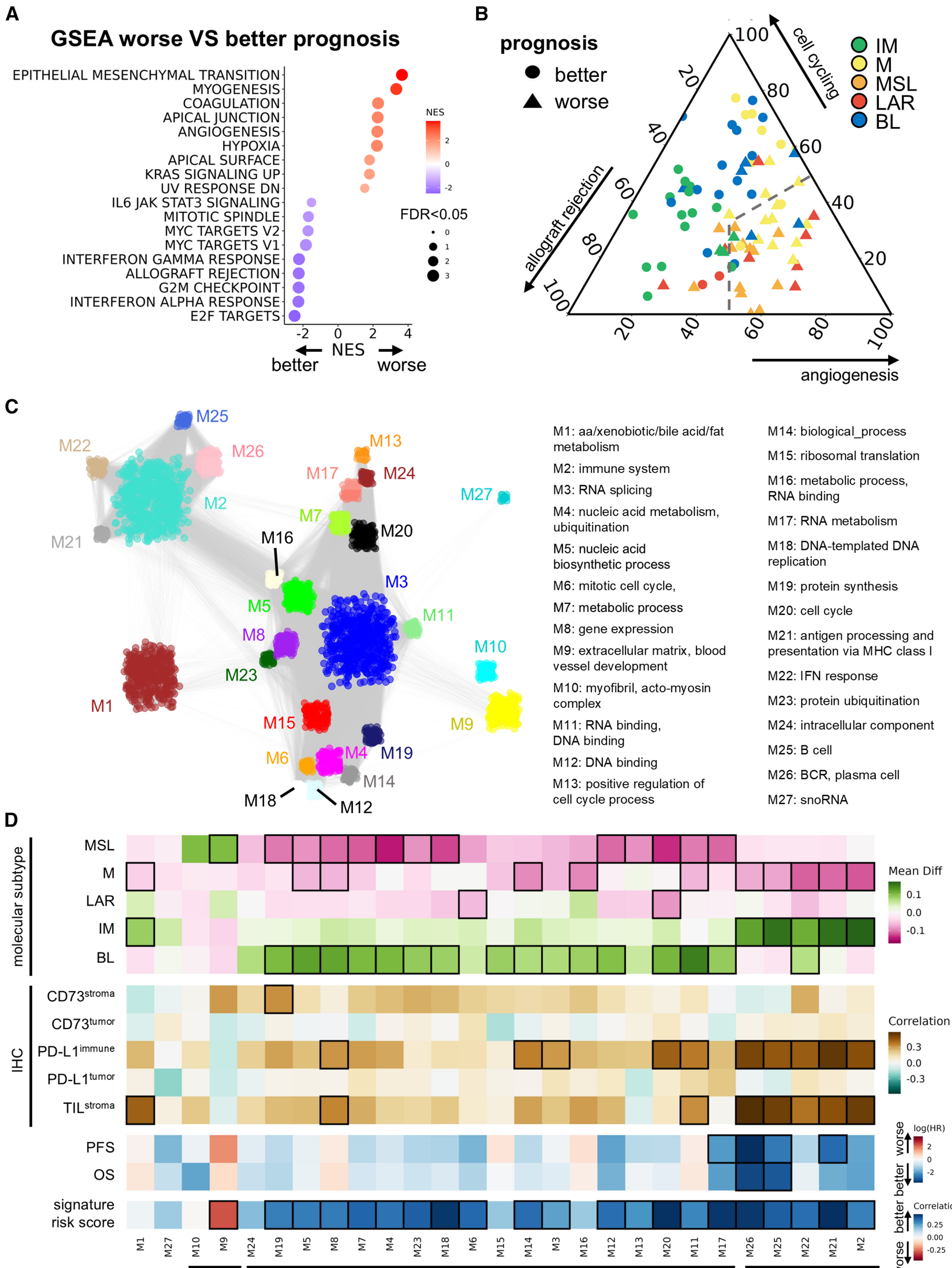

Figure 3. Baseline RNA-seq characterization of SYNERGY

(A) GSEA of hallmark gene sets comparing signature risk groups. Only hallmarks with FDR<0.05 are shown. NES, normalized enrichment score; FDR, false discovery rate.

(legend continued on next page)

($p = 0.026$), while PFS showed a non-significant trend ($p = 0.43$) (Figures S2G and S2H). We next assessed the signature in the TBCRC-043 phase 2 trial ($n = 106$), in which patients with metastatic TNBC were randomized to carboplatin alone or carboplatin plus atezolizumab across first-, second-, and third-line metastatic settings (NCT03206203).[24] In the atezolizumab plus carboplatin arm, no significant stratification of PFS could be observed (Figure S2I). This likely reflects fundamental differences in the tumor microenvironment (TME) composition between cohorts, as evidenced by markedly lower PD-L1 positivity in TBCRC-043 compared to SYNERGY, both on immune cells (20% vs. 69%) and tumor cells (4% vs. 7%).[12,24]

Transcriptomic subtyping captures response heterogeneity in metastatic TNBC

Molecular subtyping of TNBC was originally established in primary tumors and has been extensively characterized in the early-stage setting.[4,6–8] Yet, whether these subtypes are preserved in metastatic biopsies and whether the ICI benefit differs across them remains unknown. We, therefore, first asked whether transcriptomic subtyping established in early TNBC is applicable to metastatic disease. Comparing subtypes in the SYNERGY cohort with primary tumors from patients with early-stage TNBC in the SCAN-B initiative[25] ($n = 649$), we found highly concordant uniform manifold approximation and projection (UMAP) distributions, indicating that the five molecular subtypes, basal-like (BL), immunomodulatory (IM), mesenchymal (M), mesenchymal stem-like (MSL), and luminal androgen receptor (LAR),[4,6,7] are preserved in metastatic TNBC (Figure 2H).

We then explored the relationship between these molecular subtypes, the transcriptomic risk signature, and clinical outcome. Across first-line metastatic cohorts (SYNERGY, SPECTA, and AURORA), BL and IM tumors displayed higher (i.e., better-outcome) signature risk scores than M, MSL, and LAR subtypes (Figure 2I). In the SYNERGY cohort, IM tumors were associated with more favorable outcomes, whereas MSL tumors exhibited the worst prognosis (Figure S3A). In multivariable Cox regression models including both subtype and the transcriptomic risk score, the latter remained significantly associated with PFS, demonstrating prognostic value beyond molecular subtype classification (Figure S3B). Similar results were observed in the combined SPECTA and AURORA first-line chemo-ICI cohort, despite limited sample size (Figures S3C and S3D).

In contrast, in AURORA patients treated with first-line systemic therapy without immunotherapy, subtype-associated outcomes were reversed, with IM tumors displaying worse outcomes and MSL tumors showing more favorable prognosis (Figure S3E), echoing the inversion observed for the transcriptomic risk signature (Figure S2C). In this setting, the risk score did not retain independent significance in multivariable analysis (Figure S3F).

Altogether, these findings indicate that while BL and IM subtypes associate with better risk scores, the transcriptomic risk signature provides additional prognostic information for chemo-ICI response beyond subtype classification alone.

Unraveling the transcriptomic architecture of the TME associated with chemo-ICI response

To further dissect the biological interpretation of the risk signature, we performed gene set enrichment analysis (GSEA) comparing patients with better versus worse outcomes based on the signature risk score in the SYNERGY cohort. Hallmark gene sets related to immune functions and cell cycling were enriched in patients with better outcomes, while those related to (myo)fibrovascular pathways were enriched in patients with worse outcomes (Figure 3A). Given the central role of these three features, we visualized GSVA scores for hallmarks "allograft rejection," "angiogenesis," and a cell cycle gene set for each sample in a ternary plot (Figure 3B). Interestingly, MSL and LAR samples generally had a worse outcome and were clustered mainly toward the angiogenesis apex. This highlights that high angiogenic transcription, low proliferation, and low immune infiltration are emblematic of worse outcomes to chemo-ICI. Notably, angiogenic and proliferation-related pathways were specifically captured by the signature stratification but not by CB-based stratification (Figure S1L), suggesting these programs contribute to longer-term PFS beyond the CB endpoint. Intriguingly, tumors of the M subtype were distributed among both prognostic groups, with the key distinction being their proliferation status (Figure 3B). This suggests that baseline proliferation status alone may define the response of M tumors to chemo-ICI.

In addition, we performed a weighted gene correlation network analysis[26] (WGCNA) on baseline RNA-seq of the SYNERGY cohort. This identified 27 modules of co-expressed genes, each capturing distinct biological processes (Figure 3C; Table S4). For each sample, a module eigengene was calculated for every module that summarizes its overall gene expression. A correlation analysis between the module eigengenes identified three "supermodules" of highly correlating modules, representing (myo)fibrovascular-related genes, immune system genes, and a group of modules covering cell cycle, gene expression, and translation (Table S5). Next, we correlated the module eigengenes with baseline clinico-pathological and molecular tumor characteristics, as well as with patient survival outcomes (Figure 3D).

Two modules, M25 and M26, demonstrated a statistically significant association with improved PFS and OS. These two modules were enriched with genes related to B cells and plasma cells, respectively. Additionally, two modules, M17 (enriched for RNA metabolism) and M21 (representing peptide processing and MHC-I presentation), showed a significant association with improved PFS but not with OS. Overall, only

(B) Ternary plot of GSVA scores for hallmarks allograft rejection, angiogenesis, and a cell cycling gene set. Dashed line indicates the angiogenesis apex.
(C) WGCNA network plot: genes function as nodes and edges connect gene pairs with strong co-expression.
(D) Heatmap showing the associations between module gene expression and baseline tumor characteristics and survival outcomes. Continuous features by two-sided Spearman correlation test (FDR<0.05), binary features by two-sided Wilcoxon rank-sum test (FDR<0.05), and OS/PFS by univariate Cox regression (likelihood ratio test $p < 0.05$). Black outlined boxes indicate significant associations.

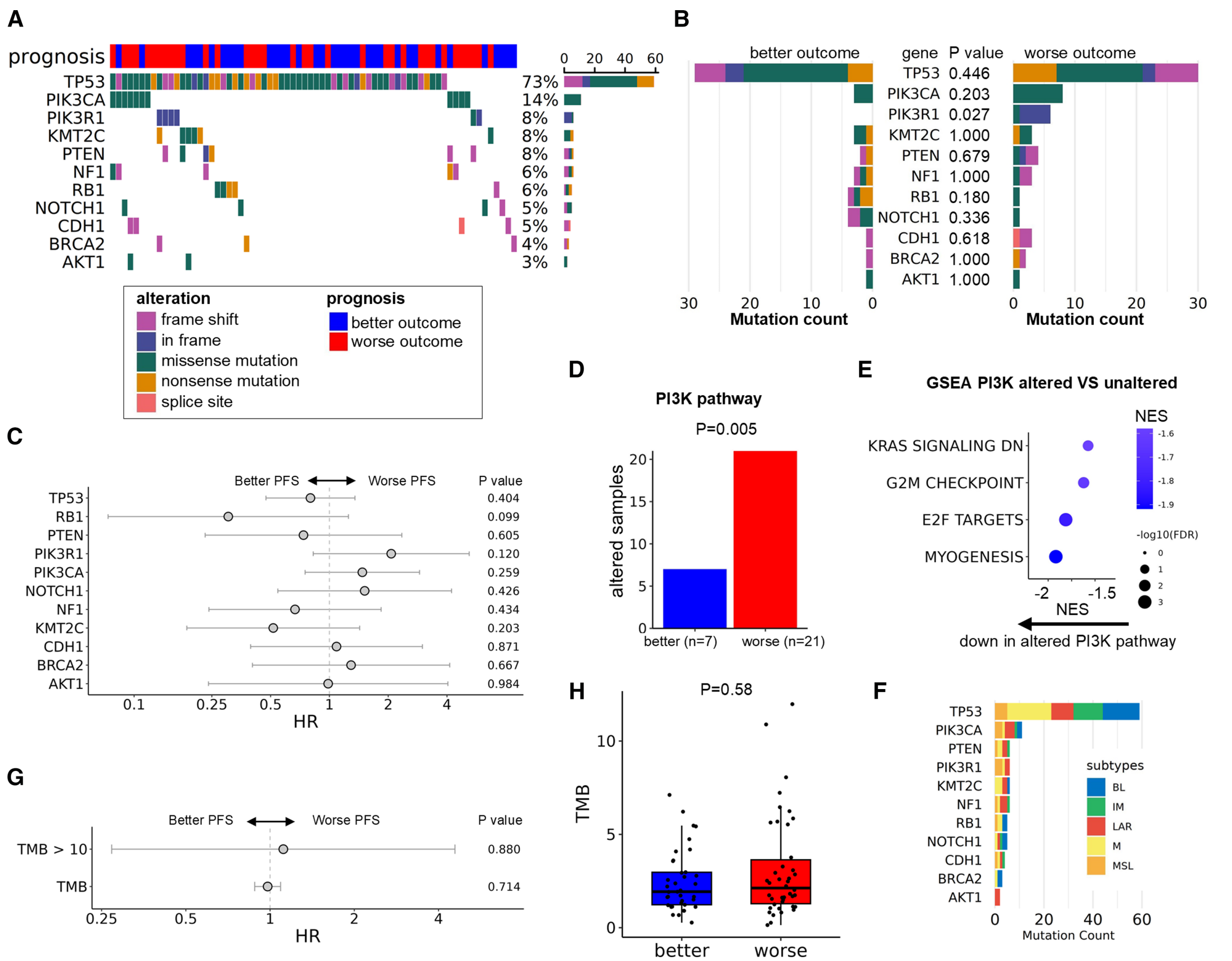

Figure 4. WES analysis at baseline in SYNERGY

(A) Oncoplot showing distribution of the key driver mutations; percentages indicate fraction of samples harboring a mutation.

(B) Bar plot comparing driver mutation frequency between transcriptomic risk groups. *p* values indicate two-sided Fisher's exact test.

(C) Univariate Cox regression for PFS across selected driver mutations. HR, hazard ratio. Whiskers indicate 95% confidence interval. *p* values indicate two-sided Wald test.

(D) Bar plot showing the number of patients with at least one mutation in PI3K pathway genes in transcriptomic risk groups. *p* values indicate two-sided Fisher's exact tests.

(E) GSEA of baseline RNA-seq comparing tumors with altered versus unaltered PI3K pathway.

(F) Distribution of TNBC subtypes by driver mutation status.

(G) Association between TMB and PFS by univariate Cox regression. Whiskers indicate 95% confidence interval. *p* values indicate two-sided Wald test.

(H) Boxplot comparing TMB levels between transcriptomic risk groups. Boxplots indicate the range of the central 50% of the data with the central line marking the median. Whiskers extend to 1.5× the interquartile range. *p* values indicate two-sided Wilcoxon rank-sum test.

the fibrovascular module (M9) was strongly correlated with a worse outcome as defined by our signature risk score. In contrast, modules related to cell cycle (M20) and immune response (M2, M21, M22, M25, and M26) were associated with a better outcome. These immune-related modules were significantly associated with higher levels of stromal tumor-infiltrating lymphocytes (TILs), with increased PD-L1 IHC expression on immune cells, and with the IM subtype. Conversely, they showed an inverse significant correlation with the M subtype, indicating that M tumors are characterized by a low immune cell infiltration. Additionally, MSL was the only subtype to demonstrate a significant correlation to the fibrovascular M9 module, once again linking MSL to fibrosis and worse outcome.

WES reveals an association between PI3K pathway alterations and worse outcome

Given the marked transcriptional differences between outcome groups, we investigated whether specific genomic alterations could account for these patterns by performing WES of baseline tumors (n = 79). This identified 10,889 somatic mutations (Table S6), with the highest frequencies observed in known

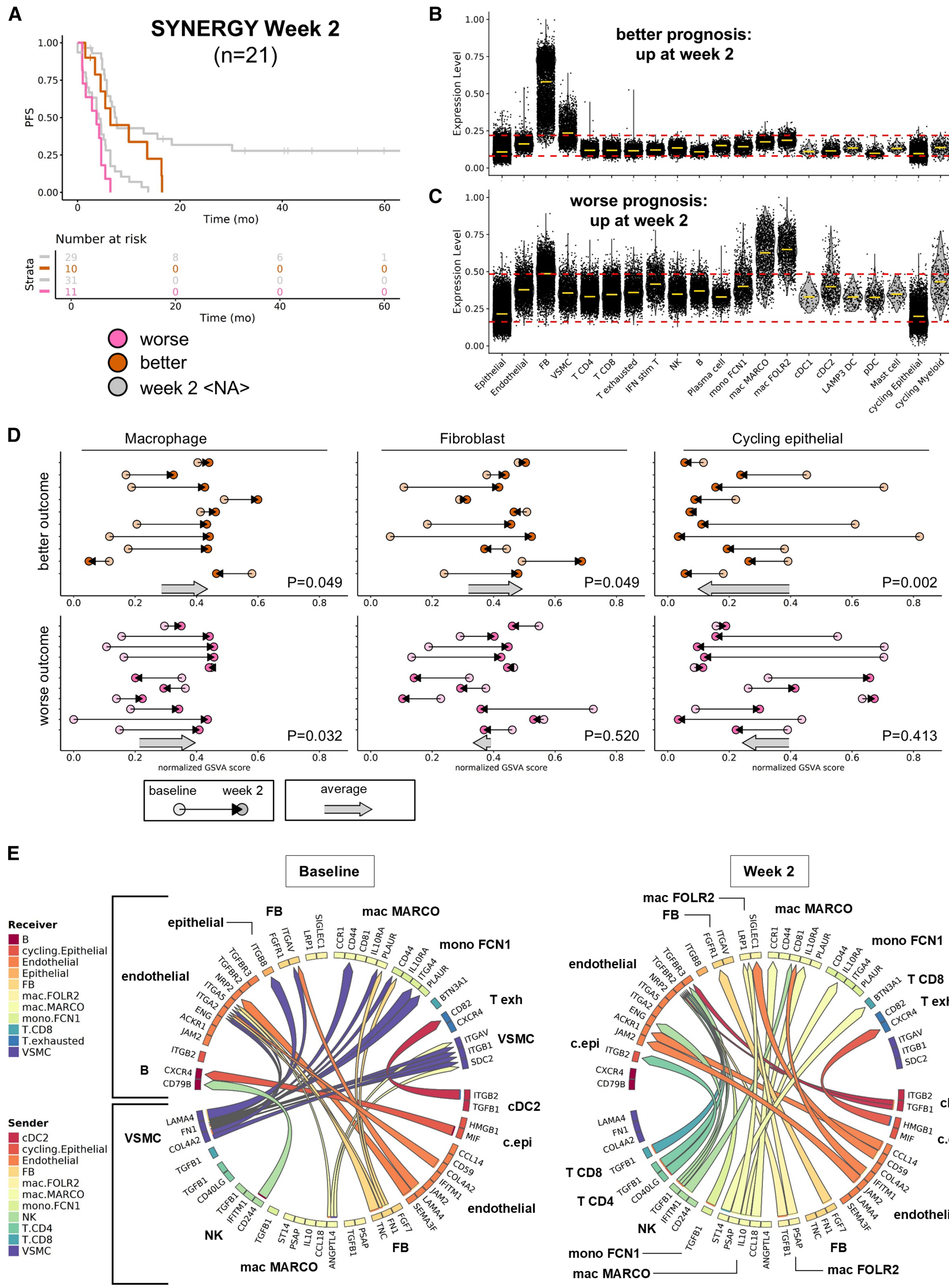

(legend on next page)

TNBC driver genes,[8,27] including *TP53* (n = 58, 73%) and *PIK3CA* (n = 11, 14%) (Figure 4A).

We compared driver mutation frequencies between outcome groups as defined by the risk signature (Figure 4B). None were significantly associated with PFS (Figure 4C). However, PI3K/AKT/mTOR pathway mutations were significantly more frequent in worse outcome tumors as defined by the risk signature (p = 0.005, Figure 4D), suggesting a potential role in chemo-ICI resistance. Consistently, PI3K pathway-altered tumors showed repression in cell cycling pathways (G2M checkpoint and E2F targets) (Figure 4E), suggesting that PI3K alterations convey a low proliferation phenotype with a worse outcome in the context of chemo-ICI. Furthermore, *PIK3CA* mutations were also commonly observed in LAR tumors, exhibiting a more immunosuppressive TME, consistent with previous studies in early-stage TNBC[6] (Figure 4F). In addition, tumor mutational burden (TMB) was not significantly associated with PFS (Figure 4G), or outcome group (Figure 4H), suggesting that TMB is not an informative biomarker for chemo-ICI response in this context.

Chemo-ICI induces early changes in the TME

To explore early transcriptional changes induced by chemo-ICI, we analyzed paired bulk RNA-seq data from baseline and week 2 biopsies (n = 21, SYNERGY cohort), evenly distributed over the two treatment arms, with 11 of 21 patients having received oleclumab. All patients with available paired samples had a relatively poor outcome, with a median PFS of less than 10 and 5 months for the "better" (n = 10) and "worse" (n = 11) outcome groups, respectively (Figure 5A). It should thus be taken into account that patients with exceptionally long PFS are absent from this analysis.

First, we identified genes up- or downregulated after 2 weeks of treatment (Figure S4A). To determine the cellular origin of these transcriptional changes, we leveraged the single-cell atlas of SYNERGY[13] (Figures S5A and S5B) and projected these genes onto this single-cell reference. Downregulated genes were predominantly expressed in cycling epithelial cells (Figure S4B), suggesting reduced proliferation or diminished tumor cell abundance. In contrast, upregulated genes localized mainly to fibroblasts and macrophages (Figure S4C), pointing to fibrotic TME remodeling and macrophage expansion.

To better understand how these changes relate to treatment outcomes, we stratified patients based on the risk signature. In patients with better outcomes, downregulated genes were highly expressed in cycling epithelial cells (Figures S4D and S4E), while upregulated genes were expressed in fibroblasts (Figure 5B). In worse outcome patients, downregulated genes did not display a specific cellular expression pattern (Figures S4F and S4G), while upregulated genes were highly expressed in macrophages (Figure 5C).

These findings suggest that the decrease in proliferating tumor cells and increase in fibroblasts occurred mainly in patients with relatively better PFS. In contrast, the increase in macrophages was observed predominantly in patients with worse PFS. This divergence indicates that the transcriptional activity of these three cell types varies with clinical outcomes. To further explore this, we first identified marker genes for fibroblasts, macrophages, and cycling epithelial cells from the SYNERGY scRNA-seq landscape and calculated GSVA scores for these marker genes for each sample collected at baseline and week 2. We constructed a ternary plot and visualized the individual components separately for each paired sample, showing the individual patient trajectories on treatment (Figure 5D). This indicates that upon treatment, patients with relatively better outcomes showed a significant decrease in proliferation (p = 0.002) and an increase in fibroblasts (p = 0.049), and also in macrophages (p = 0.049), whereas patients with the worst outcomes showed only a significant increase in macrophages (p = 0.032).

To characterize potential changes in intercellular communication induced by 2 weeks of chemo-ICI, we applied MultiNicheNet[28] to the scRNA-seq data of SYNERGY. The top 50 ligand-receptor interactions revealed a reconfiguration of the cell-cell signaling circuitry upon treatment (Figure 5C). At week 2, macrophage-derived IL-10 signaling could be seen, both in an autocrine fashion and toward monocytes. Additionally, TGFB1-mediated communication between immune cells and endothelial cells was observed, whereas the baseline communication detected between vascular smooth muscle cells (VSMCs) was lost after 2 weeks of therapy, further highlighting the dynamic remodeling of vascular signaling networks under treatment pressure. Furthermore, a self-reinforcing feedback loop from fibroblasts signaling through a FGF7-FGFR1 ligand-receptor interaction could be seen, suggesting the implication of FGFR1 as a fibrogenic actor.

Strikingly, these immunosuppressive signaling patterns were not unique to the on-treatment setting. A MultiNicheNet analysis comparing baseline tumor samples from patients with better versus worse prognosis revealed the same immunosuppressive axes observed in worse outcome tumors: TGFB1 signaling converging on endothelial receptors, macrophage-derived IL-10 signaling in an autocrine fashion, and a strong angiogenic signal characterized by monocyte-derived VEGFA signaling toward endothelial cells (Figure S5C). Together, these findings suggest that the on-treatment TME reconfiguration in refractory patients recapitulates key features of the intrinsically worse prognosis state already present at baseline.

Figure 5. Temporal tissue adaptation to chemo-immunotherapy in SYNERGY

(A) Kaplan-Meier curves stratified by risk signature and availability of on-treatment RNA-seq samples. <NA>, not available.

(B) Upregulated genes in on-treatment bulk RNA-seq of better prognosis patients projected on SYNERGY scRNA-seq landscape. Dashed lines indicate 10th and 90th percentiles of all module scores. Yellow lines indicate median.

(C) Similarly, for worse prognosis patients.

(D) Individual components of the ternary plot of normalized GSVA scores for macrophage, fibroblast, and cycling gene sets. Patient-level arrows indicate change from baseline to week 2. Gray arrows indicate average. p values indicate two-sided Wilcoxon signed-rank test.

(E) Differences in predicted ligand-receptor activity between patients at baseline and on treatment. The top 50 differential ligand-receptor pairs are shown.

CellPress OPEN ACCESS

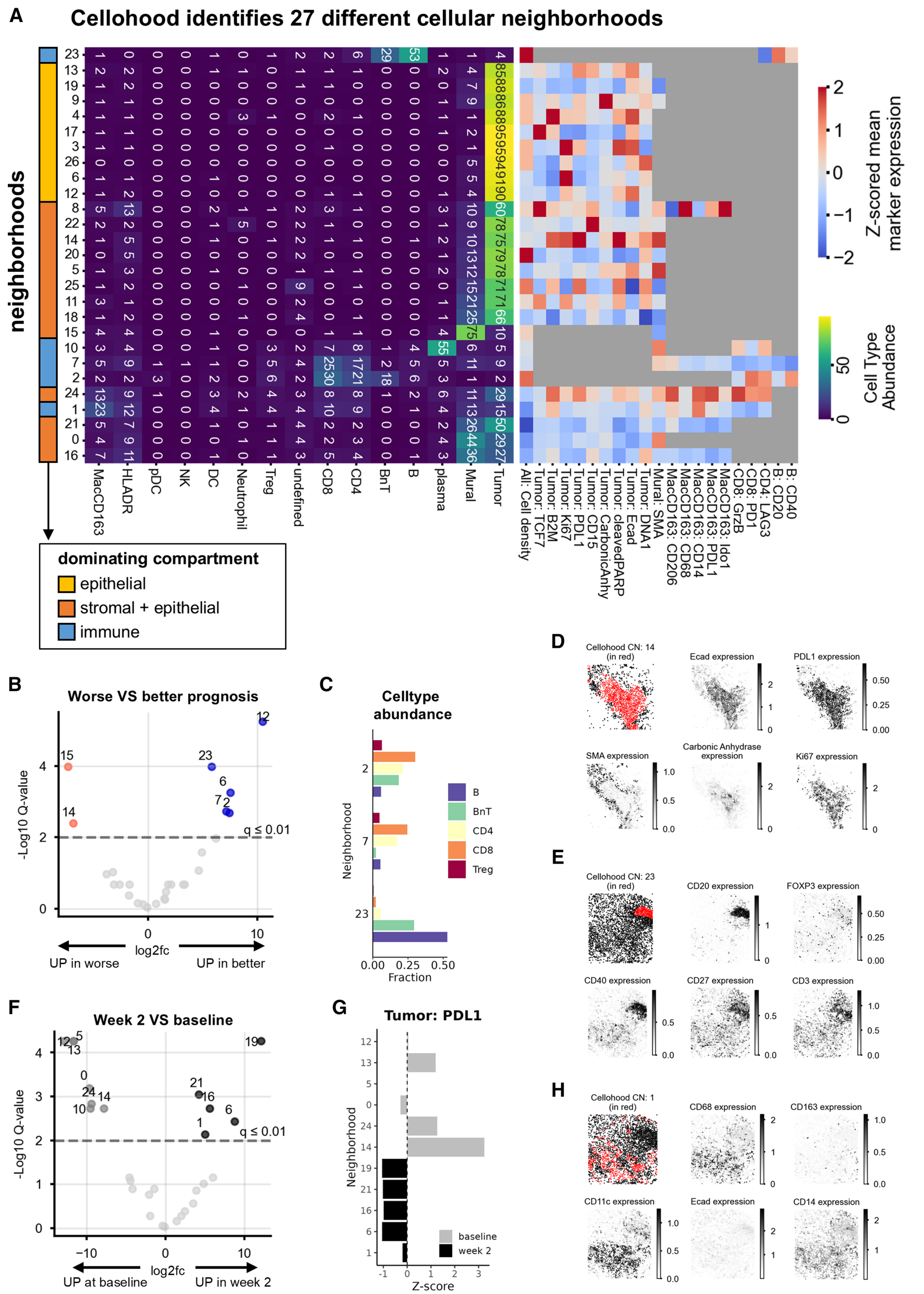

Figure 6. Cellular neighborhood analysis in SYNERGY using Cellohood

(A) Neighborhoods with cell-type abundance (% of cells) (left panel) and standardized marker gene expression (right panel). Gray tiles indicate underrepresented cell types (≤5%) in the neighborhood when marker expression is not computed.

(legend continued on next page)

Spatial organization of the immune compartment is associated with clinical outcome

To complement the transcriptomic findings with an independent layer of biological information, we next characterized the TME at the protein level using mIF and IMC, covering 16 (3 panels) and 40 markers, respectively, to generate spatially resolved tissue profiles.[29] Baseline mIF analysis showed that higher dendritic cell levels were associated with improved PFS and OS, and PD1$^+$ cell densities with better OS (Figures S6A and S6B). IMC further revealed that plasma cell abundance was positively correlated with both OS and PFS, and plasmacytoid dendritic cells (pDCs), with improved PFS (Figures S6C and S6D).

We next compared baseline TME composition according to the risk signature-defined outcome groups. Tumors from patients with better outcomes exhibited higher densities of CD4$^+$ T cells, CD8$^+$ T cells, Tregs, granzyme B$^+$ T cells, B cells, and plasma cells, along with increased tumor cellularity. In contrast, worse outcome tumors showed fewer immune features but instead displayed higher densities of PDGFRB$^+$ and SMA$^+$ mural cells, i.e., perivascular smooth muscle cells and activated contractile fibroblasts that represent the tumor stroma (Figures S6E and S6F). Representative mIF and IMC images for both outcomes are shown in Figures S6G and S6H. To investigate TME changes induced by treatment, we analyzed paired tumor biopsies (baseline and week 2) from 21 patients stratified by the risk signature. Consistent with the transcriptomic data, IMC analysis showed an increase in macrophages on-treatment in patients with worse outcomes (Figures S6I and S6J).

Because individual cell-type densities provided limited explanatory power for clinical outcomes, particularly in mIF and only marginally more so in IMC (Figure S6), we next integrated spatial tissue context using Cellohood. This transformer-based autoencoder generates fixed-size representations of cellular neighborhoods (CNs), enabling systematic analysis of tissue architecture across patients.[30] Applied to baseline and week 2 IMC slides from the SYNERGY cohort, this model identified 27 distinct CNs, each characterized by a specific cellular composition and spatial features (Figure 6A).

At baseline, tumors with worse transcriptomic risk scores exhibited neighborhoods enriched in PDGFRB$^+$ and SMA$^+$ "mural cells," indicative of fibrotic remodeling (Figures 6A and 6B). For example, CN15 represented an almost exclusively mural cell-dominant environment, while CN14 captured hypoxic carbonic anhydrase$^+$, PD-L1$^+$ tumor cells embedded within a mural cell-rich neighborhood (Figure 6D). In contrast, patients with better outcomes demonstrated CNs with low mural cell content, such as CN6 and CN12, as well as immune-enriched CNs like CN2, CN7, and CN23 (Figures 6B and 6C). Notably, CN23 displayed hallmarks of a functional tertiary lymphoid structure (TLS)-like organization,[31] including spatially organized clusters of CD20$^+$ CD27$^+$ CD40$^+$ B cells adjacent to T cells (Figure 6E). These markers indicate activated memory B cells, suggesting local antigen-driven maturation processes consistent with TLS activity. Such features are undetectable via conventional H&E staining. This finding suggests that mural cell enrichment may hinder the establishment of organized TLS structures, potentially restricting effective anti-tumor immunity.

Upon chemo-ICI treatment, we observed a reduction in PD-L1$^+$ tumor-associated neighborhoods (CN12, CN13, CN14, and CN24), which were replaced by PD-L1$^-$ tumor CNs (CN19 and CN6) and mixed stromal-immune neighborhoods containing mural cells, macrophages, and PD-L1$^-$ tumor cells (CN21, CN16, and CN1) (Figures 6F and 6G). These shifts suggest a remodeling of tumor architecture under therapeutic pressure, potentially involving PD-L1 downregulation. Notably, the fibrotic immune-desert CN0 disappeared upon treatment, while macrophage-enriched stromal neighborhoods (CN21, CN16, and CN1) appeared (Figures 6A and 6H), highlighting treatment-associated recruitment of macrophages within a stromal, PD-L1$^-$ tumor environment in metastatic TNBC.

DISCUSSION

We performed a multi-omics analysis of advanced TNBC patients from the IMMUcan project, integrating bulk and scRNA-sequencing, IHC, WES, mIF, and IMC. At baseline, tumors from patients with better survival outcomes displayed enriched immune activity, higher proliferative status, and spatial colocalization of B and T cells. In contrast, poor prognosis was linked to a mural cell-rich angiogenic and fibrotic TME characterized by low proliferation and limited immune infiltration. These findings align with previous reports suggesting that fibrosis may contribute to immune evasion by creating a mechanical barrier impeding immune cell infiltration and by promoting immunosuppressive signaling.[32] Cellohood analysis further demonstrated that immune cells were spatially organized into aggregates resembling functional TLS in tumors with better outcomes, whereas fibrotic tumors lacked TLS. This suggests that stromal remodeling may be associated with a reduced capacity to support TLS formation, potentially limiting local anti-tumor immune priming.[33]

We also observed an increased frequency of PI3K pathway alterations in patients with worse prognosis. In TNBC, *PIK3CA* mutations have been associated with poor prognosis and resistance to chemotherapy, in early disease settings.[34–36] Mechanistically, PI3K/mTOR activation has been linked to upregulation of PD-L1,[37] reduced antigen presentation, and modulation of myeloid polarization,[38] whereas pathway inhibition can enhance dendritic cell, CD8$^+$ T cell, and NK cell infiltration and improve response to ICI.[39] Thus, PI3K/AKT/mTOR alterations may contribute to poor

(B) Differential presence of neighborhoods in samples at baseline with a worse compared to better prognosis.
(C) Highlight of B and T cell abundance in selected neighborhoods.
(D and E) (D) Example of CN14 in a worse prognosis sample and (E) CN23 in a better prognosis sample at baseline.
(F) Differential presence of neighborhoods at baseline compared to week 2.
(G) Highlight of standardized PD-L1 protein expression on epithelial tumor cells in selected neighborhoods.
(H) Example of CN1 of a week 2 sample with increased macrophage signal.
BnT, ambiguous B or T cell in close proximity; HLADR, HLA-DR$^+$ cell lacking canonical lineage markers. q values indicate FDR-corrected two-sided t test.

outcomes through a dual mechanism of enhanced tumor survival and immune evasion.[40]

We further examined longitudinal TME changes upon treatment with chemo-ICIs. On-treatment biopsies showed fibrovascular reconstruction, macrophage expansion, and reduced PD-L1 expression on tumor cells along with increased expression of TGFβ1 and IL-10, suggesting the emergence of alternative immunosuppressive programs under therapeutic pressure. Our findings highlight the need for combination strategies targeting immune checkpoints alongside pathways involved in fibrosis, angiogenesis, and/or immunosuppressive macrophages. Ongoing development of agents such as bispecific antibodies against PD-(L)1-VEGFA[41,42] or antiangiogenic/antifibrotic tyrosine kinase inhibitors in combination with anti-PD-(L)1 antibodies[43] may help overcome resistance. Compounds that target immunosuppressive macrophages may also help reverse or delay disease progression. PI3K pathway inhibitors also emerge as potential therapeutic interventions to counter both tumor-intrinsic aggressiveness and immunosuppression.

Finally, we assessed the generalizability of the transcriptomic signature across independent cohorts. Its performance was strongly context dependent, reflecting the substantial biological heterogeneity of metastatic TNBC. Notably, in AURORA patients treated without immunotherapy, the signature direction was reversed, with better-risk tumors showing inferior PFS, consistent with molecular subtype-specific biology. Immune-enriched tumors, typically associated with better outcomes under immunotherapy, may paradoxically carry a worse prognosis in the absence of ICI, while MSL and LAR subtypes, characterized by lower immune infiltration and indolent low-proliferation may display improved outcome in non-immunotherapy contexts.

In the TBCRC-043 cohort, the absence of signature performance may be related to differences in the study population, where patients were more heavily pre-treated compared to the patients included in the SYNERGY (first-line setting). Moreover, marked differences in PD-L1 positivity on tumor-infiltrating immune cells in TBCRC-043 suggest distinct immunological landscapes between these cohorts. This is further supported by the absence of durable long-term responders in TBCRC-043, where all patients progressed within approximately 20 months (median 4.11 months), in contrast to SYNERGY, where a subset of patients exhibited prolonged disease control exceeding 5 years. Similar context dependency has been observed in the IMpassion130 trial, where benefit from atezolizumab was largely restricted to PD-L1-positive immune-infiltrated tumors.[44,45]

Several limitations of this study warrant consideration. First, SYNERGY included patients with both PD-L1^{+} and PD-L1^{-} tumors, as the trial was designed before results became available from the two large regulatory phase 3 clinical trials[45,46] leading to the approval of chemo-ICI as SOC for first-line PD-L1^{+} TNBC. Second, the treatment landscape is rapidly evolving: the widespread adoption of neoadjuvant pembrolizumab in early TNBC will fundamentally alter the biology of patients presenting with recurrent metastatic disease, potentially rendering current findings less applicable to this emerging population. Third, paired on-treatment biopsies were unavailable for patients with durable long-term responses, limiting our ability to characterize the transcriptomic and microenvironmental features associated with exceptional long-term benefit.

Taken together, these findings add to a growing body of evidence demonstrating that TNBC is not a single disease but encompasses multiple biologically distinct entities with different molecular dependencies, immune landscapes, and therapeutic vulnerabilities. While this heterogeneity has been recognized for more than a decade,[47] it remains only partially reflected in current treatment algorithms. Moving beyond the historical TNBC label toward biologically driven classifications integrating tumor genomics and TME characteristics will likely be essential for the next generation of precision therapeutic strategies.

Looking ahead, the development of robust predictive biomarkers for metastatic TNBC will require moving beyond static, single-time-point molecular profiling toward dynamic biomarker strategies that capture the evolving tumor-immune interplay under treatment pressure. Integrating longitudinal molecular readouts with imaging, metabolic, and systemic host response parameters within adaptive clinical trial designs will be essential to identify which patients benefit from ICI-based regimens, when resistance emerges and how treatment strategies should be modified in real time.

Limitations of the study

Our translational analysis has some limitations. First, on-treatment biopsies were available for only a subset of patients, and none were obtained from patients with exceptional durable responses, largely because these patients frequently achieved early complete radiological responses precluding repeat biopsy. Consequently, we could not characterize the dynamic transcriptomic and microenvironmental changes associated with long-term benefit. Second, although we validated our transcriptomic signature across multiple independent cohorts, suitable datasets of ICI-treated advanced TNBC remained limited. Available cohorts differed in treatment line, therapeutic regimen, and baseline immune characteristics, particularly PD-L1 prevalence, highlighting the biological and clinical heterogeneity of advanced TNBC and emphasizing that biomarker performance is highly context dependent. Finally, although our multi-omics approach integrated bulk transcriptomics, WES, single-cell transcriptomics, and spatial proteomics (mIF and IMC), it did not include spatial transcriptomics. Future studies combining spatial transcriptomics with high-dimensional imaging may provide an even more comprehensive understanding of the cellular interactions underlying the fibrovascular and myeloid resistance programs identified here.

RESOURCE AVAILABILITY

Lead contact

Further information and requests should be directed to the lead contact, Laurence Buisseret (laurence.buisseret@bordet.be).

Materials availability

This study did not generate any new materials.

Data and code availability

- Bulk RNA and WES sequencing data for EORTC-1553-SPECTA and SYNERGY are deposited in the European Genome-Phenome

Archive (EGA) under accession numbers EGAS50000001587 and EGAS50000002010, respectively. IMC and mIF data for EORTC-1553-SPECTA and SYNERGY are available (Zenodo: https://doi.org/10.5281/zenodo.21538432). All IMMUcan data are available in line with EORTC data sharing policy (https://www.eortc.org/app/uploads/2023/06/L-01-POL-01.pdf). Requests can be submitted at https://www.eortc.be/services/forms/erp/request.aspx. Single-cell RNA-seq data of SYNERGY is available (EGA: EGAD50000000748). RNA-seq data for NCT03206203 (TBCRC-043) are available (NCBI: PRJNA995589). RNA-seq data for NCT03121352 are available (NCBI: PRJNA1010464). RNA-seq data for TONIC are available (EGA: EGAS00001003535). RNA-seq data for AURORA are available through the AURORA data sharing portal (https://aurora-ctc.hubruxelles.be/) upon approval of a research proposal and execution of a data transfer agreement.

- The code to reproduce the figures is available (GitHub: https://github.com/BCTL-Bordet/IMMUcan-BC1 and Zenodo: https://doi.org/10.5281/zenodo.21538432). The code for Cellohood is available (Github: https://github.com/marmarmarmar/cellohood).
- Any additional information required to reanalyze the data reported in this work is available from the lead contact upon request.

ACKNOWLEDGMENTS

The authors thank all patients who provided samples and their families and the Institut Jules Bordet as sponsor of the SYNERGY study. SYNERGY was conducted with financial support from AstraZeneca/MedImmune, which provided the study drugs (durvalumab and oleclumab) and a funding contribution for the trial. None of the funders had any role in the study design, data collection, data analysis, data interpretation, or writing of the report. We thank Andres Ocampo for helpful discussions. The authors acknowledge support from Le Fonds de la Recherche Scientifique (FNRS), La Fondation contre le Cancer (grant FCC C/2022/2057), and L'Association Jules Bordet (grant AJB-2021-25). IMMUcan funding acknowledgment: IMI2 JU grant agreement 821558, supported by EU's Horizon 2020 and EFPIA. This publication reflects the authors' view and that neither IMI nor the EU, EFPIA is responsible for any use that may be made of the information contained therein. The SPECTA Platform is supported by Alliance Healthcare. Alliance Healthcare will become Cencora. E.S. acknowledges the support from the Polish National Science Centre SONATA BIS grant no. 2020/38/E/NZ2/00305. M.R. is supported by an AIRC fellowship for Italy.

AUTHOR CONTRIBUTIONS

C.S. and L.B. conceived and designed this study. All authors were involved in data acquisition. N.V.R., E.A., and A.J.G. analyzed the data. All authors were involved in data interpretation. N.V.R., E.A., A.J.G., and L.B. wrote the manuscript. N.V.R., E.A., and A.J.G. produced the manuscript figures and tables. H.S.H. and M. Morfouace. oversaw all molecular and cellular tumor profiling efforts in IMMUcan. All authors were involved in reviewing and editing drafts of the paper and approving the manuscript.

DECLARATION OF INTERESTS

E.A. served on the advisory board or received honoraria from Eli Lilly, AstraZeneca, Abscint, Bayer, Gilead, and Novartis; received research grant to her institution from Gilead BeLux; and received meeting/travel grants from Novartis, Roche, Eli Lilly, Daiichi Sankyo, AstraZeneca, Abscint, Menarini, and Gilead. H.S.H. is an employee of the Healthcare business of Merck KGaA, Darmstadt, Germany. D.L. serves on the advisory board of AstraZeneca, ExactSciences, Gilead, MSD, Novartis, and Pfizer; is an invited speaker or other for AstraZeneca, Gilead, Lilly, MSD, Novartis, Pfizer, Roche; and receives travel/congress grants from AstraZeneca, Gilead, MSD, Novartis, Pfizer, and Roche. P.A. serves as a consultant for Boehringer Ingelheim, Macrogenics, Roche, Novartis, Amcure, Servier, G1 Therapeutics, Radius, Deloitte, Daiichi Sankyo, and Olema and receives honoraria from Synthon, Amgen, Novartis, Gilead, Lilly, and Menarini and travel grants from Amgen, MSD, Pfizer, Roche, and Daiichi Sankyo. His institution receives research funding from Roche. H.W.'s institution received financial compensation on his behalf for advisory boards, lecture fees, and/or consultancy fees from Agendia, AstraZeneca, Augustine Therapeutics NV, Boehringer Ingelheim int., Daiichi-Sankyo, Eli Lily Benelux N.V., Gilead Sciences, Immutep Limited, MediMix bv, Menarini, Novartis, NV Hict, NV Roche SA, Pfizer SA, PSI Cro AG, Seagen, Stemline Therapeutics, and Syneos Health. He received travel support from Gilead Sciences. E.R. received institutional grants from BMS, AstraZeneca, and Replimune; research fundings from Fondation Amgen, Fondation BMS, and Amgen; and travel grants from Roche, BMS, and Merck. A.G. has financial interests and receives institutional funding from AstraZeneca. C.S. serves on the advisory board of AMGEN, Astellas, Cepheid, Merck & Co, Seattle Genetics, and Signatur Biosciences; is an invited speaker for Exact Sciences and Prime oncology; receives travel support from Pfizer and Roche; and holds stocks/shares in Signatur Biosciences. L.B.'s salary is partly covered by the Fondation contre le Cancer (Belgium). She is a member of the IMMUcan Consortium (EORTC). Her institution receives research funding from Astra Zeneca/MedImmune for the SYNERGY and Neo-CheckRay trials. Her institution received financial compensation for advisory roles, lectures, or consultancies from Domain Therapeutics, iTeos Therapeutics, AstraZeneca, Roche, and Novartis and travel grants from GILEAD Sciences, AstraZeneca, Roche, and Eli Lily. Projects in Szczurek lab at the University of Warsaw are co-funded by Merck Healthcare. A.L.G-Z.: institutional grant by Pfizer; advisory by Novartis, Palex, Pfizer, AstraZeneca, and Pierre Fabre; travel grant by Roche, Pfizer, and Novartis; and speaker honoraria by Novartis, Pfizer, Lilly, and AstraZeneca. Travel grant: AstraZeneca, Gilead, Menarini, and Novartis. J.M.B. receives research support from Genentech/Roche and Incyte Corporation; has received advisory board payments from AstraZeneca, Eli Lilly, and Mallinckrodt; and is an inventor on patents regarding immunotherapy targets and biomarkers in cancer. M.K. receives research funding to the institute from Bristol Myers Squibb (BMS), Roche, and AstraZeneca/MedImmune and an advisory role/speakers' fee (all compensated to the institute) from Alderaan, BMS, Domain Therapeutics, Medscape, Roche, MSD, and Daiichi Sankyo, outside the submitted work. B. Brasiuniene received speaker honorarium or covering conference registration fee, travel expenses from Bristol MS, MSD, Novartis, Lilly, and AstraZeneca.

DECLARATION OF GENERATIVE AI AND AI-ASSISTED TECHNOLOGIES IN THE MANUSCRIPT PREPARATION PROCESS

During the preparation of this work, the authors used ChatGPT (v.5.5) and Claude (Sonnet 4.6) in order to improve medical writing. After using these tools, the authors reviewed and edited the content as needed and take full responsibility for the content of the published article.

STAR★METHODS

Detailed methods are provided in the online version of this paper and include the following:

- KEY RESOURCES TABLE
- EXPERIMENTAL MODEL AND STUDY PARTICIPANT DETAILS
  - Ethics statement and patient population
- METHOD DETAILS
  - Sample workflow and analysis
  - Molecular sequencing
  - Bulk RNA-seq
  - Risk signature generation and risk-stratification
  - Gene ontology enrichment
  - Molecular subtypes
  - GSEA
  - WGCNA gene expression modules
  - Ternary plot
  - scRNA-seq
  - Whole-exome sequencing
  - Slide preparation
  - Spectral IF

- ○ Imaging mass cytometry (IMC)
- ○ IMC downstream processing
- ○ Cellohood
- ○ Immunohistochemistry
- ○ TILs scoring
- QUANTIFICATION AND STATISTICAL ANALYSIS
- ADDITIONAL RESOURCES

SUPPLEMENTAL INFORMATION

Supplemental information can be found online at https://doi.org/10.1016/j.xcrm.2026.103080.

Received: December 25, 2025
Revised: June 19, 2026
Accepted: September 3, 2026
Published: September 29, 2026

REFERENCES

1. Gennari, A., André, F., Barrios, C.H., Cortés, J., de Azambuja, E., DeMichele, A., Dent, R., Fenlon, D., Gligorov, J., Hurvitz, S.A., et al. (2021). ESMO Clinical Practice Guideline for the diagnosis, staging and treatment of patients with metastatic breast cancer. Ann. Oncol. *32*, 1475–1495. https://doi.org/10.1016/j.annonc.2021.09.019.

2. Debien, V., De Caluwé, A., Wang, X., Piccart-Gebhart, M., Tuohy, V.K., Romano, E., and Buisseret, L. (2023). Immunotherapy in breast cancer: an overview of current strategies and perspectives. Npj Breast Cancer *9*, 7. https://doi.org/10.1038/s41523-023-00508-3.

3. Wang, X., Collet, L., Rediti, M., Debien, V., De Caluwé, A., Venet, D., Romano, E., Rothé, F., Sotiriou, C., and Buisseret, L. (2023). Predictive Biomarkers for Response to Immunotherapy in Triple Negative Breast Cancer: Promises and Challenges. J. Clin. Med. *12*, 953. https://doi.org/10.3390/jcm12030953.

4. Lehmann, B.D., Bauer, J.A., Chen, X., Sanders, M.E., Chakravarthy, A.B., Shyr, Y., and Pietenpol, J.A. (2011). Identification of human triple-negative breast cancer subtypes and preclinical models for selection of targeted therapies. J. Clin. Investig. *121*, 2750–2767. https://doi.org/10.1172/JCI45014.

5. Burstein, M.D., Tsimelzon, A., Poage, G.M., Covington, K.R., Contreras, A., Fuqua, S.A.W., Savage, M.I., Osborne, C.K., Hilsenbeck, S.G., Chang, J.C., et al. (2015). Comprehensive genomic analysis identifies novel subtypes and targets of triple-negative breast cancer. Clin. Cancer Res. *21*, 1688–1698. https://doi.org/10.1158/1078-0432.CCR-14-0432.

6. Bareche, Y., Venet, D., Ignatiadis, M., Aftimos, P., Piccart, M., Rothe, F., and Sotiriou, C. (2018). Unravelling triple-negative breast cancer molecular heterogeneity using an integrative multiomic analysis. Ann. Oncol. *29*, 895–902. https://doi.org/10.1093/annonc/mdy024.

7. Wang, X., Venet, D., Lifrange, F., Larsimont, D., Rediti, M., Stenbeck, L., Dupont, F., Rouas, G., Garcia, A.J., Craciun, L., et al. (2024). Spatial transcriptomics reveals substantial heterogeneity in triple-negative breast cancer with potential clinical implications. Nat. Commun. *15*, 10232. https://doi.org/10.1038/s41467-024-54145-w.

8. Jiang, Y.-Z., Ma, D., Suo, C., Shi, J., Xue, M., Hu, X., Xiao, Y., Yu, K.-D., Liu, Y.-R., Yu, Y., et al. (2019). Genomic and Transcriptomic Landscape of Triple-Negative Breast Cancers: Subtypes and Treatment Strategies. Cancer Cell *35*, 428–440.e5. https://doi.org/10.1016/j.ccell.2019.02.001.

9. Fan, L., Wang, Z.-H., Ma, L.-X., Wu, S.-Y., Wu, J., Yu, K.-D., Sui, X.-Y., Xu, Y., Liu, X.-Y., Chen, L., et al. (2024). Optimising first-line subtyping-based therapy in triple-negative breast cancer (FUTURE-SUPER): a multi-cohort, randomised, phase 2 trial. Lancet Oncol. *25*, 184–197. https://doi.org/10.1016/S1470-2045(23)00579-X.

10. Liu, Y., Zhu, X.-Z., Xiao, Y., Wu, S.-Y., Zuo, W.-J., Yu, Q., Cao, A.-Y., Li, J.-J., Yu, K.-D., Liu, G.-Y., et al. (2023). Subtyping-based platform guides precision medicine for heavily pretreated metastatic triple-negative breast cancer: The FUTURE phase II umbrella clinical trial. Cell Res. *33*, 389–402. https://doi.org/10.1038/s41422-023-00795-2.

11. Liu, Y., Yang, F., Zuo, W., Liu, X.Y., Gong, Y., Shen, Y., Liang, Q., Di, G., Li, J., Liu, G., et al. (2026). Impact of bevacizumab on the efficacy of antibody–drug conjugates as later-line treatment of basal-like immune-suppressed triple-negative breast cancer. J. Clin. Oncol. *44*, 1020. https://doi.org/10.1200/jco.2026.44.16_suppl.1020.

12. Buisseret, L., Loirat, D., Aftimos, P., Maurer, C., Punie, K., Debien, V., Kristanto, P., Eiger, D., Goncalves, A., Ghiringhelli, F., et al. (2023). Paclitaxel plus carboplatin and durvalumab with or without oleclumab for women with previously untreated locally advanced or metastatic triple-negative breast cancer: the randomized SYNERGY phase I/II trial. Nat. Commun. *14*, 7018. https://doi.org/10.1038/s41467-023-42744-y.

13. Metoikidou, C., Karnaukhov, V., Boeckx, B., Timperi, E., Bonté, P.-E., Wang, L., Espenel, M., Albaud, B., Loirat, D., Wang, X., et al. (2025). Continuous replenishment of the dysfunctional CD8 T cell axis is associated with response to chemoimmunotherapy in advanced breast cancer. Cell Rep. Med. *6*, 101973. https://doi.org/10.1016/j.xcrm.2025.101973.

14. Agostinetto, E., Venet, D., Krisanto, P., Loirat, D., Aftimos, P.G., Denis, Z., Wildiers, H., Van Renne, N., Ghiringhelli, F., de Azambuja, E., et al. (2025). First-line durvalumab plus chemotherapy with or without oleclumab for locally advanced or metastatic triple-negative breast cancer: SYNERGY overall survival and circulating tumor DNA analysis. J. Clin. Oncol. *43*, 1097. https://doi.org/10.1200/JCO.2025.43.16_suppl.1097.

15. Hoshida, Y., Villanueva, A., Kobayashi, M., Peix, J., Chiang, D.Y., Camargo, A., Gupta, S., Moore, J., Wrobel, M.J., Lerner, J., et al. (2008). Gene expression in fixed tissues and outcome in hepatocellular carcinoma. N. Engl. J. Med. *359*, 1995–2004. https://doi.org/10.1056/NEJMoa0804525.

16. Van Renne, N., Ballet, F., Van Hees, S., Cuypers, B., Furquim d'Almeida, A., Vanderlinden, A., Meysman, P., Feld, J.J., Sangro Del Alcázar, P., Bourgeois, S., et al. (2026). A liver gene signature links liver cancer risk in chronic viral hepatitis to intrahepatic IgA plasma cells. JHEP Rep. JHEP Rep. *8*, 101714. https://doi.org/10.1016/j.jhepr.2025.101714.

17. Buisseret, L., Pommey, S., Allard, B., Garaud, S., Bergeron, M., Cousineau, I., Ameye, L., Bareche, Y., Paesmans, M., Crown, J.P.A., et al. (2018). Clinical significance of CD73 in triple-negative breast cancer: multiplex analysis of a phase III clinical trial. Ann. Oncol. *29*, 1056–1062. https://doi.org/10.1093/annonc/mdx730.

18. Hoshida, Y. (2010). Nearest template prediction: a single-sample-based flexible class prediction with confidence assessment. PLoS One *5*, e15543. https://doi.org/10.1371/journal.pone.0015543.

19. Cimino-Mathews, A., Ye, X., Meeker, A., Argani, P., and Emens, L.A. (2013). Metastatic triple-negative breast cancers at first relapse have fewer tumor-infiltrating lymphocytes than their matched primary breast tumors: a pilot study. Hum. Pathol. *44*, 2055–2063. https://doi.org/10.1016/j.humpath.2013.03.010.

20. Caballero, C., Irrthum, A., Goulioti, T., Cameron, D., Norton, L., and Piccart, M. (2023). International research to address the challenges of metastatic breast cancer: the AURORA Program (BIG 14-01). NPJ Breast Cancer *9*, 42. https://doi.org/10.1038/s41523-023-00548-9.

21. Aftimos, P., Oliveira, M., Irrthum, A., Fumagalli, D., Sotiriou, C., Gal-Yam, E.N., Robson, M.E., Ndozeng, J., Di Leo, A., Ciruelos, E.M., et al. (2021). Genomic and Transcriptomic Analyses of Breast Cancer Primaries and Matched Metastases in AURORA, the Breast International Group (BIG) Molecular Screening Initiative. Cancer Discov. *11*, 2796–2811. https://doi.org/10.1158/2159-8290.CD-20-1647.

22. Voorwerk, L., Slagter, M., Horlings, H.M., Sikorska, K., van de Vijver, K.K., de Maaker, M., Nederlof, I., Kluin, R.J.C., Warren, S., Ong, S., et al. (2019). Immune induction strategies in metastatic triple-negative breast cancer to enhance the sensitivity to PD-1 blockade: the TONIC trial. Nat. Med. *25*, 920–928. https://doi.org/10.1038/s41591-019-0432-4.

23. Wilkerson, A.D., Parthasarathy, P.B., Stabellini, N., Mitchell, C., Pavicic, P.G., Fu, P., Rupani, A., Husic, H., Rayman, P.A., Swaidani, S., et al.

(2024). Phase II Clinical Trial of Pembrolizumab and Chemotherapy Reveals Distinct Transcriptomic Profiles by Radiologic Response in Metastatic Triple-Negative Breast Cancer. Clin. Cancer Res. *30*, 82–93. https://doi.org/10.1158/1078-0432.CCR-23-1349.

24. Lehmann, B.D., Abramson, V.G., Dees, E.C., Shah, P.D., Ballinger, T.J., Isaacs, C., Santa-Maria, C.A., An, H., Gonzalez-Ericsson, P.I., Sanders, M.E., et al. (2024). Atezolizumab in Combination With Carboplatin and Survival Outcomes in Patients With Metastatic Triple-Negative Breast Cancer: The TBCRC 043 Phase 2 Randomized Clinical Trial. JAMA Oncol. *10*, 193–201. https://doi.org/10.1001/jamaoncol.2023.5424.

25. Saal, L.H., Vallon-Christersson, J., Häkkinen, J., Hegardt, C., Grabau, D., Winter, C., Brueffer, C., Tang, M.-H.E., Reuterswärd, C., Schulz, R., et al. (2015). The Sweden Cancerome Analysis Network - Breast (SCAN-B) Initiative: a large-scale multicenter infrastructure towards implementation of breast cancer genomic analyses in the clinical routine. Genome Med. *7*, 20. https://doi.org/10.1186/s13073-015-0131-9.

26. Langfelder, P., and Horvath, S. (2008). WGCNA: an R package for weighted correlation network analysis. BMC Bioinf. *9*, 559. https://doi.org/10.1186/1471-2105-9-559.

27. Cancer Genome Atlas Network (2012). Comprehensive molecular portraits of human breast tumours. Nature *490*, 61–70. https://doi.org/10.1038/nature11412.

28. Browaeys, R., Gilis, J., Sang-Aram, C., De Bleser, P., Hoste, L., Tavernier, S., Lambrechts, D., Seurinck, R., and Saeys, Y. (2023). MultiNicheNet: a flexible framework for differential cell-cell communication analysis from multi-sample multi-condition single-cell transcriptomics data. Preprint at bioRxiv. https://doi.org/10.1101/2023.06.13.544751.

29. Eling, N., Dorier, J., Rusakiewicz, S., Liechti, R., Devanand, P., Daniel, M., Windhager, J., Fernandez, B.P., Déglise, S., Despland, L., et al. (2025). Multi-modal image analysis for large-scale cancer tissue studies within IMMUcan. Cell Rep. Methods *5*, 101170. https://doi.org/10.1016/j.crmeth.2025.101170.

30. Możejko, M., Schulz, D., Gogolewski, K., Eling, N., Krawczyk, J., Bogatyrova, O., Golab, J., Nowis, D., Liechti, R., Morfouace, M., et al. (2025). Cellohood: multi-granular discovery of cellular neighborhoods with a permutation-invariant set transformer auto-encoder. Preprint at bioRxiv. https://doi.org/10.1101/2025.11.10.687184.

31. Teillaud, J.-L., Houel, A., Panouillot, M., Riffard, C., and Dieu-Nosjean, M.-C. (2024). Tertiary lymphoid structures in anticancer immunity. Nat. Rev. Cancer *24*, 629–646. https://doi.org/10.1038/s41568-024-00728-0.

32. Caligiuri, G., and Tuveson, D.A. (2023). Activated fibroblasts in cancer: Perspectives and challenges. Cancer Cell *41*, 434–449. https://doi.org/10.1016/j.ccell.2023.02.015.

33. Sautès-Fridman, C., Petitprez, F., Calderaro, J., and Fridman, W.H. (2019). Tertiary lymphoid structures in the era of cancer immunotherapy. Nat. Rev. Cancer *19*, 307–325. https://doi.org/10.1038/s41568-019-0144-6.

34. Mallick, S., Duttaroy, A.K., and Dutta, S. (2024). The PIK3CA gene and its pivotal role in tumor tropism of triple-negative breast cancer. Transl. Oncol. *50*, 102140. https://doi.org/10.1016/j.tranon.2024.102140.

35. Pascual, J., and Turner, N.C. (2019). Targeting the PI3-kinase pathway in triple-negative breast cancer. Ann. Oncol. *30*, 1051–1060. https://doi.org/10.1093/annonc/mdz133.

36. Hu, H., Zhu, J., Zhong, Y., Geng, R., Ji, Y., Guan, Q., Hong, C., Wei, Y., Min, N., Qi, A., et al. (2021). PIK3CA mutation confers resistance to chemotherapy in triple-negative breast cancer by inhibiting apoptosis and activating the PI3K/AKT/mTOR signaling pathway. Ann. Transl. Med. *9*, 410. https://doi.org/10.21037/atm-21-698.

37. Mansour, F.A., Al-Mazrou, A., Al-Mohanna, F., Al-Alwan, M., and Ghebeh, H. (2020). PD-L1 is overexpressed on breast cancer stem cells through notch3/mTOR axis. Oncoimmunology *9*, 1729299. https://doi.org/10.1080/2162402X.2020.1729299.

38. Collet, L., Venet, D., Karaköse, B., Saura Manich, C., Nuciforo, P.G., Fesl, C., Zoppoli, G., von Moos, R.A.F., Prat, A., André, F., et al. (2025). 116MO PIK3CA helical but not kinase mutations and enrichment of M2 macrophages predict response to taselisib in the LORELEI phase II randomized trial. Ann. Oncol. *36*, S240–S241. https://doi.org/10.1016/j.annonc.2025.08.550.

39. Bergholz, J.S., Wang, Q., Wang, Q., Ramseier, M., Prakadan, S., Wang, W., Fang, R., Kabraji, S., Zhou, Q., Gray, G.K., et al. (2023). PI3Kβ controls immune evasion in PTEN-deficient breast tumours. Nature *617*, 139–146. https://doi.org/10.1038/s41586-023-05940-w.

40. Teo, Z.L., Versaci, S., Dushyanthen, S., Caramia, F., Savas, P., Mintoff, C.P., Zethoven, M., Virassamy, B., Luen, S.J., McArthur, G.A., et al. (2017). Combined CDK4/6 and PI3Kα Inhibition Is Synergistic and Immunogenic in Triple-Negative Breast Cancer. Cancer Res. *77*, 6340–6352. https://doi.org/10.1158/0008-5472.CAN-17-2210.

41. Ouyang, Q., Wang, X., Tian, C., Shao, X., Huang, J., Chen, Z.-H., wang, Y., Sun, T., Yi, T., Yu, X., et al. (2024). 347MO The safety and efficacy of ivonescimab in combination with chemotherapy as first-line (1L) treatment for triple-negative breast cancer (TNBC). Ann. Oncol. *35*, S360–S361. https://doi.org/10.1016/j.annonc.2024.08.295.

42. Wu, J., Zhang, J., Tong, Z., Zhang, Q., Wang, Y., Cheng, Q., Chen, X., Li, Z., Yin, Y., Du, Y., and Meng, Y. (2024). 348MO A phase Ib/II study to assess the safety and efficacy of PM8002/BNT327 in combination with nab-paclitaxel for first-line treatment of locally advanced or metastatic triple-negative breast cancer. Ann. Oncol. *35*, S361. https://doi.org/10.1016/j.annonc.2024.08.296.

43. Zhang, L., and Sun, T. (2024). Abstract PO2-06-09: A prospective phase 2 study on efficacy and safety of AK105, anlotinib combined with nab-paclitaxel (nab-P) as a first-line therapy in patients(pts) with advanced triple-negative breast cancer(TNBC). Cancer Res. *84*, PO2. https://doi.org/10.1158/1538-7445.SABCS23-PO2-06-09.

44. Emens, L.A., Goldstein, L.D., Schmid, P., Rugo, H.S., Adams, S., Barrios, C.H., Schneeweiss, A., Dieras, V., Iwata, H., Chang, C.W., et al. (2021). The tumor microenvironment (TME) and atezolizumab + nab-paclitaxel (A+nP) activity in metastatic triple-negative. breast cancer (mTNBC), IMpassion130.

45. Schmid, P., Adams, S., Rugo, H.S., Schneeweiss, A., Barrios, C.H., Iwata, H., Diéras, V., Hegg, R., Im, S.-A., Shaw Wright, G., et al. (2018). Atezolizumab and Nab-Paclitaxel in Advanced Triple-Negative Breast Cancer. N. Engl. J. Med. *379*, 2108–2121. https://doi.org/10.1056/NEJMoa1809615.

46. Cortes, J., Rugo, H.S., Cescon, D.W., Im, S.-A., Yusof, M.M., Gallardo, C., Lipatov, O., Barrios, C.H., Perez-Garcia, J., Iwata, H., et al. (2022). Pembrolizumab plus Chemotherapy in Advanced Triple-Negative Breast Cancer. N. Engl. J. Med. *387*, 217–226. https://doi.org/10.1056/NEJMoa2202809.

47. Carey, L., Winer, E., Viale, G., Cameron, D., and Gianni, L. (2010). Triple-negative breast cancer: disease entity or title of convenience? Nat. Rev. Clin. Oncol. *7*, 683–692. https://doi.org/10.1038/nrclinonc.2010.154.

48. Joaquin Garcia, A., Rediti, M., Venet, D., Majjaj, S., Kammler, R., Munzone, E., Gianni, L., Thürlimann, B., Laáng, I., Colleoni, M., et al. (2023). Differential Benefit of Metronomic Chemotherapy Among Triple-Negative Breast Cancer Subtypes Treated in the IBCSG Trial 22–00. Clin. Cancer Res. *29*, 4908–4919. https://doi.org/10.1158/1078-0432.CCR-23-1267.

49. Hao, Y., Hao, S., Andersen-Nissen, E., Mauck, W.M., Zheng, S., Butler, A., Lee, M.J., Wilk, A.J., Darby, C., Zager, M., et al. (2021). Integrated analysis of multimodal single-cell data. Cell *184*, 3573–3587.e29. https://doi.org/10.1016/j.cell.2021.04.048.

50. Dyer, N.P., Shahrezaei, V., and Hebenstreit, D. (2019). LiBiNorm: an htseq-count analogue with improved normalisation of Smart-seq2 data and library preparation diagnostics. PeerJ *7*, e6222. https://doi.org/10.7717/peerj.6222.

51. Patro, R., Duggal, G., Love, M.I., Irizarry, R.A., and Kingsford, C. (2017). Salmon provides fast and bias-aware quantification of transcript expression. Nat Methods *14*, 417–419. https://doi.org/10.1038/nmeth.4197.

CellPress
OPEN ACCESS

52. Bolger, A.M., Lohse, M., and Usadel, B. (2014). Trimmomatic: a flexible trimmer for Illumina sequence data. Bioinformatics *30*, 2114–2120. https://doi.org/10.1093/bioinformatics/btu170.

53. Windhager, J., Zanotelli, V.R.T., Schulz, D., Meyer, L., Daniel, M., Bodenmiller, B., and Eling, N. (2023). An end-to-end workflow for multiplexed image processing and analysis. Nat. Protoc. *18*, 3565–3613. https://doi.org/10.1038/s41596-023-00881-0.

54. Dobin, A., Davis, C.A., Schlesinger, F., Drenkow, J., Zaleski, C., Jha, S., Batut, P., Chaisson, M., and Gingeras, T.R. (2013). STAR: ultrafast universal RNA-seq aligner. Bioinformatics *29*, 15–21. https://doi.org/10.1093/bioinformatics/bts635.

55. Ono, A., Goossens, N., Finn, R.S., Schmidt, W.N., Thung, S.N., Im, G.Y., and Hoshida, Y.; Precision Liver Cancer Prevention Consortium (2017). Persisting risk of hepatocellular carcinoma after hepatitis C virus cure monitored by a liver transcriptome signature. Hepatology *66*, 1344–1346. https://doi.org/10.1002/hep.29203.

56. Friedrich, M., Pohin, M., Jackson, M.A., Korsunsky, I., Bullers, S.J., Rue-Albrecht, K., Christoforidou, Z., Sathananthan, D., Thomas, T., Ravindran, R., et al. (2021). IL-1-driven stromal-neutrophil interactions define a subset of patients with inflammatory bowel disease that does not respond to therapies. Nat. Med. *27*, 1970–1981. https://doi.org/10.1038/s41591-021-01520-5.

57. Korsunsky, I., Millard, N., Fan, J., Slowikowski, K., Zhang, F., Wei, K., Baglaenko, Y., Brenner, M., Loh, P.-R., and Raychaudhuri, S. (2019). Fast, sensitive and accurate integration of single-cell data with Harmony. Nat. Methods *16*, 1289–1296. https://doi.org/10.1038/s41592-019-0619-0.

58. Greenwald, N.F., Miller, G., Moen, E., Kong, A., Kagel, A., Dougherty, T., Fullaway, C.C., McIntosh, B.J., Leow, K.X., Schwartz, M.S., et al. (2022). Whole-cell segmentation of tissue images with human-level performance using large-scale data annotation and deep learning. Nat. Biotechnol. *40*, 555–565. https://doi.org/10.1038/s41587-021-01094-0.

STAR★METHODS

KEY RESOURCES TABLE

REAGENT or RESOURCE	SOURCE	IDENTIFIER
Antibodies		
CD15 – mIF	Biolegend	Cat#301902; RRID:AB_314194
panCK - mIF	DAKO	Cat#M3515; RRID:AB_2132885
CD3 - mIF	DAKO	Cat#A0452; RRID:AB_2335677
CD11c – mIF	Cell Marque	Cat#111M-15; RRID:AB_3714915
CD20 – mIF	DAKO	Cat#M0755; RRID:AB_2282030
CD163 – mIF	Histoline	Cat#MB460; RRID: AB_3714914
PD1 - mIF	Biocare	clone NAT105; Cat#ACI3137
PDL1 - mIF	Cell Signaling	Cat#13684; RRID:AB_2687655
GB - mIF	Monosan	Cat#MON7029C; RRID: AB_2114692
Ki67 - mIF	CellMarque	clone SP6; Cat#275 R; RRID: AB_1158037
CD8 - mIF	CellMarque	clone SP16; Cat#108 R; RRID: AB_2892088
CD4 - mIF	CellMarque	clone SP35; Cat#104 R; RRID: AB_1516770
FoxP3 - mIF	Abcam	Cat#Ab99963; RRID:AB_10675258
CD56 - mIF	CellMarque	clone MRQ-42; Cat#156 R; RRID: AB_2941091
MPO – IMC	Dako	Cat#A0398; RRID:AB_2335676
Histone H3 – IMC	CST	Cat#4499BF; RRID:AB_10544537
SMA – IMC	Thermo Fisher	Cat#14–9760-82; RRID:AB_2572996
CD16 – IMC	Abcam	Cat#ab215977; RRID:AB_2877105
CD38 – IMC	Abcam	Cat#ab176886; RRID:AB_2864383
HLA-DR – IMC	Abcam	Cat#ab20181; RRID:AB_445401
CD27 – IMC	R&D Systems	Cat#AF382; RRID:AB_355338
CD15 – IMC	BioLegend	Cat#301902; RRID:AB_314194
CD45RA – IMC	BioLegend	Cat#304102; RRID:AB_314406
CD163 – IMC	Thermo Fisher	Cat#MA1-82342; RRID: AB_2275716
B2M – IMC	CST	Cat#47847
CD20 – IMC	Thermo Fisher	Cat#14–0202-82; RRID:AB_10734340
CD68 - IMC	Thermo Fisher	Cat#14–0688-82; RRID:AB_11151139
IDO1 – IMC	Abcam	Cat#ab245737; RRID:AB_2894840
CD3 –IMC	Dako	Cat#A0452; RRID:AB_2335677
LAG-3 – IMC	CST	Cat#25848; RRID:AB_2943248
CD11c – IMC	CST	Cat#45581BF; RRID:AB_2799286
PD1 – IMC	CST	Cat#86163; RRID:AB_2728833
PDGFRb – IMC	Abcam	Cat#ab215978; RRID:AB_2894841
CD7 – IMC	Abcam	Cat#ab230834; RRID:AB_2889384
GZMB – IMC	CST	Cat#79903; RRID:AB_3665532
PD-L1 – IMC	Abcam	Cat#ab226766; RRID:AB_3073663
TCF7 – IMC	CST	Cat#85942;RRID:AB_3675764
CD45RO – IMC	BioLegend	Cat#304202; RRID:AB_314418
FOXP3 – IMC	Thermo Fisher	Cat#14–4777-82; RRID:AB_467556
ICOS – IMC	CST	Cat#39740; RRID:AB_3676096
CD8a – IMC	Thermo Fisher	Cat#14–0085-82; RRID:AB_11150240
CAIX – IMC	R&D Systems	Cat#AF2188; RRID:AB_416562

(*Continued on next page*)

Continued

REAGENT or RESOURCE	SOURCE	IDENTIFIER
CD33 - IMC	R&D Systems	Cat#MAB11371; RRID:AB_2889385
Ki67 – IMC	BD Biosciences	Cat#556003; RRID:AB_396287
VISTA – IMC	CST	Cat#82119; RRID:AB_3683060
CD40 – IMC	Abcam	Cat#ab228818; RRID:AB_2889383
CD4 – IMC	Abcam	Cat#ab181724; RRID:AB_2864377
CD14 – IMC	Abcam	Cat#ab230903; RRID:AB_2889378
E-cadherin – IMC	BD Biosciences	Cat#610182; RRID:AB_397581
CD303 - IMC	R&D Systems	Cat#AF1376; RRID:AB_354762
CD206 – IMC	R&D Systems	Cat#MAB25341; RRID:AB_10890782
Cleaved PARP – IMC	BD Biosciences	Cat#552596; RRID:AB_394437
panCK-Alexa488 – IF-IMC-co-stain	Thermo Fisher	Cat#53–9003-82; RRID:AB_1834350
CD45-DyLight550 – IF-IMC-co-stain	Novus Biologicals	Cat#NBP2-34528 R; RRID:AB_3287685
CD163-Alexa647 – IF-IMC-co-stain	Abcam	Cat#Ab218294; RRID:AB_2943126
Discovery OmniMap anti-rabbit HRP	Ventana	Cat#760–4311
Discovery OmniMap anti-mouse HRP	Ventana	Cat#760–4310
PD-L1 - IHC	Ventana	Cat#740–4907; RRID:AB_3698154
CD73 - IHC	Abcam	Cat#ab124725; RRID:AB_10976033
**Chemicals, peptides, and recombinant proteins**		
DAPI	Akoya	Cat#FP1490
Iridium intercalator	Standard Biotools	Cat#201192C
Antibody stabilizing solution	Candor Biosciences	Cat#130125
**Critical commercial assays**		
Maxpar labeling kits	Standard Biotools	Cat#201300
AllPrep DNA/RNA FFPE	Qiagen	Cat#80234
KAPA RNA HyperPrep Kit with RiboErase (HMR)	Roche	Cat#08098140702
SMART-Seq Stranded	Takara Bio	Cat#634444
**Deposited data**		
EORTC-1553-SPECTA RNA and WES sequencing	This publication; European Genome-Phenome archive	EGAS50000001587
EORTC-1553-SPECTA IMC and mIF	This publication; Zenodo	https://doi.org/10.5281/zenodo.21538432
SYNERGY RNA and WES sequencing	This publication; European Genome-Phenome archive	EGAS50000002010
SYNERGY IMC and mIF	This publication; Zenodo	https://doi.org/10.5281/zenodo.21538432
Code to reproduce the figures and analyses	This publication; Zenodo	https://doi.org/10.5281/zenodo21538432
SYNERGY scRNA-seq	Metoikidou et al.[13] Cell Reports Medicine; European Genome-Phenome Archive	EGAD50000000748
NCT03206203 (TBCRC-043) RNA-seq	Lehmann et al.[24]; JAMA Oncology	NCBI PRJNA995589
NCT03121352 RNA-seq	Wilkerson et al.[23] Clinical Cancer Research	NCBI PRJNA1010464
TONIC RNA-seq	Voorwerk et al.[22] Nature Medicine	EGAS00001003535
AURORA RNA-seq	Breast International Group (BIG), Aftimos et al.[21] Cancer Discovery, 2021	https://aurora.bigagainstbreastcancer.org
**Software and algorithms**		
Code to reproduce figures for this publication	This publication	https://github.com/BCTL-Bordet/IMMUcan-BC1/and https://doi.org/10.5281/zenodo.21538432
TNBC molecular subtyping	Joaquín-García et al. 2023 Clin. Cancer Res.[48]	https://github.com/BCTL-Bordet/TNBC_molecularsubtypes
VEGAN version 2.1.1	Paul-Antoine Nicolas	https://github.com/bioinfo-pf-curie/vegan https://doi.org/10.5281/zenodo.12167637

(*Continued on next page*)

Continued

REAGENT or RESOURCE	SOURCE	IDENTIFIER
STAR version 2.7.9a_2021–06-25	Alex Dobin	https://github.com/alexdobin/STAR
R statistical software (v4.4.1)	R Core Team	https://www.r-project.org/
GATK (v4.2.4.1)	Broad Institute	https://gatk.broadinstitute.org/
Seurat (v5.1.0 for scRNA-seq integration and v5.3.0 for downstream analyses)	Hao et al.[49]; Nature Biotechnology	https://satijalab.org/seurat/
WGCNA (v1.72–5)	Langfelder and Horvath.[26]; BMC Bioinformatics	https://cran.r-project.org/src/contrib/Archive/WGCNA/WGCNA_1.72-5.tar.gz
nichenetr (v2.2.1)	Browaeys et al.[28]; Nature Methods	https://github.com/saeyslab/nichenetr
multinichenetr (v2.1.0)	Browaeys et al.[28]; BioRxiv	https://github.com/saeyslab/multinichenetr
biomaRt (v2.60.0)	Bioconductor	https://bioconductor.org/packages/biomaRt
gprofiler2 (v0.2.3)	CRAN	https://CRAN.R-project.org/package=gprofiler2
survival (v3.7–0)	CRAN	https://CRAN.R-project.org/package=survival
survminer (v0.5.0)	CRAN	https://CRAN.R-project.org/package=survminer
sva (v3.52.0)	Bioconductor	https://bioconductor.org/packages/sva
GSVA (v1.52.3)	Bioconductor	https://bioconductor.org/packages/GSVA
Cellohood	Mozejko et al.[30]; BioRxiv	https://github.com/marmarmarmar/cellohood
GSEA GUI v4.1.0	The Broad Institute	https://www.gsea-msigdb.org/gsea/index.jsp
LiBiNorm v2.5	Dyer et al.[50]; PeerJ	https://github.com/NigelDyer/LiBiNorm
Salmon (v1.10.2 for IMMUcan; v1.10.1 for NCT03206203 and NCT03121352)	Patro et al., 2017, Nature Methods[51]	https://github.com/COMBINE-lab/salmon
Trimmomatic (v0.39)	Bolger et al.[52]; Bioinformatics	https://github.com/usadellab/Trimmomatic
IFQuant	Eling et al.[29]; Cell Reports Medicine	https://github.com/BICC-UNIL-EPFL/IFQuant
Steinbock toolkit (v0.14.1)	Windhager et al.[53]; Nature Protocols	https://bodenmillergroup.github.io/steinbock/latest/
imcRtools	Windhager et al.[53]; Nature Protocols	https://www.bioconductor.org/packages/release/bioc/html/imcRtools.html
ZEN software (v2.6)	ZEISS	https://www.zeiss.com/microscopy/fr/produits/logiciel/zeiss-zen.html

EXPERIMENTAL MODEL AND STUDY PARTICIPANT DETAILS

Ethics statement and patient population

This research complies with all relevant ethical regulations. The SYNERGY trial was approved by the following ethical committees/authorities: Comité d'éthique hospitalo-facultaire Erasme-ULB and Federal Agency for medicines and health products (Belgium), and Comité de protection des personnes Ile de France and Agence nationale de sécurité du medicament et des produits de santé (France). The study design and conduct complied with all relevant regulations regarding the use of human study participants and was conducted in accordance with the criteria set by the Declaration of Helsinki. All patients signed written informed consent before inclusion. The SYNERGY trial was registered on ClinicalTrials.gov (https://clinicaltrials.gov/) (ID: NCT03616886).

For EORTC-SPECTA, all patients had provided written informed consent at the time of sample collection for molecular and cellular analysis. The IMMUcan project was approved by several ethical committees; see below for the main recruiting sites: Commissie voor Medische Ethiek ZNA, Belgium on 10/03/2021; Comite de protection des personnes "Nord-Ouest I", France on 02/12/2019; Ethik-Kommission der Medizinischen Universität Wien, Austria on 15/04/2020; Cyprus National Bioethics Committee (CNBC), Cyprus on 29/06/2023; Comissão de Ética CHUPorto/ICBAS, Portugal on 03/11/2021; CES do IPO Porto, Portugal on 11/03/2021.

For patients enrolled in SYNERGY, selection criteria to be included in the advanced breast cancer cohort of IMMUcan were the following: i. Informed consent allowing for all aspects of the IMMUcan project; ii. All clinical information available before September 2021; iii. Samples no older than 2 years, for quality reasons: FFPE tissue approved after quality check performed by the central

biobank (based on % of viable tumor area and cellularity, FFPE tissue consistent with the diagnostic) and quantity assessment (enough material to perform all molecular and cellular analysis); iii. Availability of whole blood; iv. No further sample collection was mandatory, but availability of paired samples (pre-post treatment) was a plus.

For patients enrolled in the EORTC-1553-SPECTA study, selection criteria to be included in the advanced breast cancer cohort of IMMUcan were the following: i. Age at enrollment: minimum 18 years old; ii. FFPE tissue approved after quality check performed by the central biobank (based on % of viable tumor area and cellularity, FFPE tissue consistent with the diagnostic) and quantity assessment (enough material to perform all molecular and cellular analysis); iii. Availability of whole blood; iv. Written informed consent given according to ICH/GCP, and national/local regulations; v. Patient was eligible for the advanced breast cancer cohort, namely had confirmed locally advanced or metastatic TNBC, and were to be treated in first line with immune checkpoint inhibitors.

Tables S1 and S2 show the baseline characteristics of patients from the SYNERGY and the EORTC-1553-SPECTA study. Specifically, median age was 57.5 and 52 years in the SYNERGY and in the EORTC-1553-SPECTA study, respectively. All patients were of female gender. Health and immune status was assessed by the local investigators and considered permissive for the patients to receive the oncological treatment. Details on previous treatments are provided in Table S1. All patients were treated in first-line setting and did not receive any prior treatment for metastatic disease.

METHOD DETAILS

Sample workflow and analysis

FFPE samples were sent to the Integrated BioBank of Luxembourg (IBBL). Upon arrival, IBBL assessed: i. quality: an H&E staining was performed for pathology review and assessment of tumor cellularity (based on % of viable tumor area and cellularity (a minimum of 20–30% cellularity was required), FFPE tissue consistent with the diagnostic); ii. quantity: Sufficient material had to be provided to allow molecular analyses (WES, RNA-seq) as well as cellular analyses (mIF, IMC). The required number of slides was adjusted to tissue availability and tumor/stroma content. Based on tumor quality, especially percentage of tumor cells, IBBL was able to determine the adequate number of FFPE slides necessary for nucleic acid extraction.

If insufficient material was provided by the sites, or if the quality of the FFPE material was insufficient, the clinicians were asked for extra FFPE material. In case of unavailability, the patient was not included in the project.

Upon approval from EORTC (after eligibility check), IBBL cut the recommended number of 10 μm slides for molecular analyses and shipped them to CeGaT (Tubingen, Germany). Upon arrival (real time shipment), PaxDNA and PaxRNA tubes were placed at −80°C. Upon approval from EORTC (after eligibility check), IBBL extracted gDNA from the PaxDNA tube, aliquotted it for shipment to the lab performing the molecular analysis, and stored the leftover gDNA at −80°C. Upon arrival (real time shipment), all ACD tubes were processed for PBMCs isolation, count and storage in liquid nitrogen (5 million cells per aliquot). Upon arrival (real time shipment), the STRECK tube was processed to plasma and store at −80°C.

Fresh frozen tissue, serum, and plasma were shipped on dry ice, by batch, yearly. Upon arrival, IBBL stored all samples at −80°C.

Molecular sequencing

FFPE slides and associated gDNA were shipped weekly to CeGat, for molecular analysis. For each set of slides, IBBL also shared the H&E image with CeGat, using Calopix. The tissue slides were marked for macrodissection. CeGaT performed manual macrodissection, after confirmation that the blank slides corresponded to the H&E slides shared by IBBL, using Calopix. After macrodissection, CeGaT extracted DNA and RNA, using the kit AllPrep DNA/RNA FFPE (Qiagen). RNA quality and quantity were assessed using Qubit RNA (Thermo Fischer) and Bioanalyzer RNA (Agilent) and DNA quality and quantity were assessed using Qubit dsDNA BR or HS (Thermo Fischer). For WES, CeGaT used for library preparation SureSelect Human All Exon V7 (Agilent) for enrichment and sequence 2 × 100 bp, on the NovaSeq6000 with expected output of 18Gb for tumor samples and 10Gb for normal samples. For exome sequencing of gDNA from peripheral blood specimen, prepared by IBBL, CeGaT required ≥1 μg high-molecular weight DNA with a concentration ≥50 ng/μL and at least 20 μL total volume. For exome sequencing of DNA extracted from FFPE tissue, prepared by CeGaT, CeGat required ≥250 ng high-molecular weight DNA with a concentration ≥25 ng/μL and at least 20 μL total volume. In case of low DNA input, a low input protocol for which 50 to 100 ng DNA at concentrations as low as 2 ng/μL could be used. For RNAseq, CeGaT used 500 ng of RNA with a concentration ≥50 ng/μL in a volume of 10 μL and a RIN above 3.

CeGaT used KAPA RNA Hyper Prep (Roche) or SMART-Seq Stranded (Takara Bio) for the library preparation and sequencing on the NovaSeq6000, at 2 × 100 bp, at a depth of 100 Mio paired end reads. In case of lower RNA input (≥25 ng) or poor RNA quality (RIN ≥2), CeGaT generated 50 Mio paired end reads. QC data were shared with EORTC and SIB, while raw data were shared with SIB for storage, archival and bioinformatics processing. Left over samples were stored temporarily at CeGaT prior to batch shipment to IBBL for long-term storage.

Bulk RNA-seq

For BC1, reads were aligned to the reference human genome GRCh38 using STAR with default parameters (v2.7.9a_2021–06-25)[54] and GENCODE comprehensive gene annotations (release 29). The number of read counts per gene locus was summarized with LiBiNorm v2.5[50]

A custom R script was developed to assess RNA sample quality based on five metrics: DV200, percentage of unique reads, percentage of mapped reads, mean gene expression level, and the number of detected genes. Samples not meeting the predefined threshold for at least two of these metrics were deemed to fail quality control and were excluded from subsequent analyses (DV200 > 30%, unique reads >30%, mapped reads >60%, mean gene expression level between 2 and 3 CPM, number of detected genes between 14,000 and 20,000).

Transcript abundance was quantified as transcripts per million (TPM) using Salmon (v1.10.2), implemented within the nf-neoant Nextflow pipeline developed by Institut Curie (https://github.com/bioinfo-pf-curie/nf-neoant). The same genome annotation files used for STAR alignment were employed for transcript quantification to ensure consistency.

For SYNERGY, 81 baseline and 21 on-treatment high-quality RNA-seq samples were retained for downstream analysis. For SPECTA, 36 high-quality samples were available (33 baseline samples and 3 on-treatment); 17 patients were retained based on having received first line immunotherapy and samples being collected prior to treatment initiation. Transcript-level expression data were provided as TPM values, and ENSEMBL transcript IDs were annotated with GSEA GUI v4.1.0 (https://www.gsea-msigdb.org/gsea/index.jsp) using the GENCODE v29 gene annotation, and genes with multiple values were collapsed using sum of read counts. For SYNERGY, genes with a median expression of 0 were removed.

For the differential expression analysis on paired baseline and week 2 on-treatment RNA-seq samples of SYNERGY, only genes were retained that were present in both baseline and on-treatment gene expression tables. After log-transformation, a two-sided Wilcoxon signed-rank test was performed on paired baseline and week 2 RNA-seq samples. For the volcano plot, genes were considered significantly differentially expressed if they had a log2 fold-change (log2fc) in expression greater than 0.25 and a nominal p-value <0.05.

The SCAN-B dataset was described previously.[25] The SCAN-B dataset v3 was retrieved from https://data.mendeley.com/datasets/yzxtxn4nmd/3 where it is stored as "/Root/StringTie FPKM Gene Data LibProtocol adjusted/SCANB.9206.genematrix_noNeg.Rdata". Its meta data is stored in "Root/Supplemental Data Table/Supplementary Data Table 1–2023–01-13.xlsx". Only TNBC samples with well annotated clinico-pathological features and survival data were retained. Ensembl gene identifiers were mapped to gene symbols using the biomaRt package in R (biomaRt_2.60.0). For Ensembl gene identifiers mapping to multiple gene symbols, only the one with the highest mean across samples was retained.

AURORA RNA sequencing was performed as described previously.[21] Transcript-level TPM values were retrieved from the AURORA data repository and only metastatic TNBC samples were retained. Transcript-level expression was collapsed to gene-level by summing TPM values across all transcripts mapping to the same gene symbol, using the Human Ensembl Transcript ID MSigDB chip file (v2024.1).

RNA-seq of TBCRC-043 (NCT03206203) and NCT03121352 were described by Lehmann et al.[24] and Wilkerson et al.,[23] respectively. Raw sequencing .fastq files were retrieved from NCBI repository (bioproject PRJNA995589 and PRJNA1010464, respectively). Adapter trimming was performed with Trimmomatic (v0.39)[52] in paired-end mode using TruSeq3-PE adapters (settings: LEADING:3, TRAILING:3, MINLEN:30). Trimmed reads were quantified at transcript level using Salmon (v1.10.1) against a GRCh38 transcriptome reference based on GENCODE release 46, with GC bias and sequence bias correction enabled (–gcBias –seqBias). Ensembl version suffixes were removed, and transcript-level expression was collapsed to gene-level by summing TPM values per gene symbol using the Human Ensembl Transcript ID MSigDB chip file (v2024.1). For TBCRC-043 (NCT03206203), gene expression was log-transformed and batch correction between FFPE and fresh-frozen samples was performed with ComBat (SVA R package), with parametric empirical Bayes priors.

Bulk RNAseq data for the TONIC trial were generated and processed as previously described.[22]

Risk signature generation and risk-stratification

A predictive transcriptomic signature was generated from the SYNERGY cohort as described previously.[15,16] Briefly, for every gene a 'cox score' was calculated that quantifies the correlation of gene expression with time-to-disease progression (PFS events). Positive Cox scores correlate with disease progression and are thus high-risk genes, while negative Cox scores are correlated with progression-free survival, and are low-risk genes. p values for each gene were calculated using a random permutation test with 1000 permutations. The mean and standard deviations of the Cox scores in the permutations were subsequently used to standardize the Cox scores in the leave-one-sample-out cross validation to avoid a gene receiving a high Cox score due to an outlier measurement in a single sample. In each leave-one-out iteration, the Cox score was recalculated. Only genes with $p < 0.05$ in each iteration were retained in the signature.

Prediction of clinical outcome was performed with the Nearest Template Prediction (NTP) algorithm[18] using cosine similarity as a metric, and the Cox scores calculated for the gene signature as weights. For each sample, the cosine similarity to the risk signature template was used as the signature risk score, with positive cosine similarity classified as better outcome and negative cosine similarity as worse outcome. For large cohorts with sufficient sample size (SYNERGY, AURORA, TBCRC-043, TONIC), NTP was applied independently within each cohort. For TONIC, an additional three-class stratification was applied,[16] in which samples with low prediction confidence (permutation test $p > 0.05$) were assigned to an intermediate class. For smaller cohorts, cohort-specific strategies were applied. SPECTA was collected within the BC1 study using the same RNA sequencing technology and platform as SYNERGY; risk stratification was therefore performed as previously described,[55] whereby each sample was added individually to the SYNERGY reference dataset prior to NTP classification. The Wilkerson cohort (NCT03121352; baseline n = 15) was generated on a distinct

sequencing platform. Here, samples were concatenated with the SYNERGY baseline dataset, batch effects were corrected using ComBat, and NTP was subsequently applied to the combined dataset. *p*-values for Kaplan Meier curves were calculated using two-sided log rank test. *p*-values for univariate cox regression of the signature risk score (i.e., the signed cosine similarity) were calculated using two-sided Wald test.

Gene ontology enrichment

Gene-ontology over-representation analysis was carried out with the gprofiler2 R package, using the full set of expressed genes as the custom background and applying the Benjamini–Hochberg FDR correction.

Molecular subtypes

Molecular subtypes[8] calculation was performed as described previously[48] (https://github.com/BCTL-Bordet/TNBC_molecularsubtypes). *p*-values for Kaplan Meier curves were calculated using log rank test. Log-transformed RNA-seq matrices from the SYNERGY baseline samples and the SCAN-B dataset were concatenated and only genes shared in the two datasets were retained. Batch correction was performed with ComBat using the sva package. Next, only genes that define the molecular subtypes were retained. Subsequently, the top 1000 most variable genes were selected based on variance across all samples and a UMAP embedding was generated using the uwot package.

GSEA

Gene Set Enrichment Analysis (GSEA) was performed using the GSEA software (GUI v4.1.0) with the Hallmark gene set collection (h.all.v2024.1.Hs.symbols.gmt) from the Molecular Signatures Database (www.gsea-msigdb.org). Log2 ratio was used as metric, with 1000 permutations. All results have a corrected Kolmogorov-Smirnov test FDR<0.05. Hallmark 'spermatogenesis' and 'pancreas beta cells' were omitted because not considered relevant.

WGCNA gene expression modules

To identify modules of co-expressed genes, the Weighted Gene Co-expression Network Analysis (WGCNA) package[26] was applied in R and visualized as previously described.[56] In short, after log-transforming gene expression, power 12 was selected based on scale-free topology model fit and gene modules were calculated using the blockwiseModules() function from the gene expression data with signed topological overlap matrix (TOM), a minimum module size of 30 genes, and a merge cut height of 0.15. Module eigengenes, which quantify the gene expression of the modules in each sample, were calculated using the moduleEigengenes() function. For visualizing the modules as a network of co-expressed genes from RNA-seq expression data, the TOM was computed using the TOM similarityFromExpr() function with Pearson correlation. The resulting TOM was filtered using a threshold of 0.05 to retain only strong gene-gene connections. To build an edge list, the TOM was first binarized into an adjacency matrix, where values above the threshold were set to 1. The lower triangle and diagonal were removed to avoid redundancy and self-loops. The unassigned 'gray' module (M0) was removed. This filtered adjacency matrix was then converted into an edge list representing gene-gene interactions. Subgraphs corresponding to individual modules were created with the igraph package, and grouped into supermodules based on module eigengene correlation. The node coordinates were computed using the Kamada-Kawai layout with jitter and positional offsets for multi-module visualization. Finally, these coordinates were then used to plot the network using the ggplot2 R package. GO enrichment analysis was performed on the module genes using the hypergeometric test using the gprofiler2 R package.

Associations between module eigengenes and patient features consists of three parts. (1) Continuous features were tested using two-sided Spearman correlation test, (2) binary traits were tested using two-sided Wilcoxon rank-sum tests, and for both, *p*-values were Benjamini-Hochberg FDR adjusted. (3) Module eigengene associations with OS and PFS were assessed by univariate Cox proportional hazards model, using log-hazard ratios to show effect direction and two-sided likelihood-ratio tests for significance.

Ternary plot

For the baseline ternary plot, gene expression was log-transformed and GSVA scores were calculated for three gene sets: 'allograft rejection' and 'angiogenesis' from the hallmark gene set collection, and a 'cell cycling' that was extracted from Seurat v5.1.0 package's built-in cc.genes object. GSVA scores were calculated using the GSVA package in R using kcdf = "Gaussian", and subsequently min–max scaled to the [0,1] interval, and then normalized so that each sample's scaled scores summed to one. The ternary diagram was made with the ggtern package.

For the 'week 2 and baseline' ternary plot, three single-cell dataset derived gene sets were used that were described above: 'cycling epithelial', 'fibroblasts', and macrophages ['mac MARCO' & 'mac FOLR2']. Scaled and normalized GSVA scores were scaled and normalized as described above. Each component was plotted separately for each patient with an arrow connecting baseline and week 2. A summary arrow depicts the average of the samples. *p*-values were calculated using two-sided Wilcoxon signed-rank test.

scRNA-seq

The original SYNERGY single-cell Seurat object was described previously[13] and processed using the Seurat toolbox v5.1.0[49] as follows. Raw RNA unique molecular identifiers (UMI) counts were extracted from all cells for a total of 76,079 high-quality cells. For

integration, samples were merged into a single object and log-normalized using NormalizeData(). Highly variable features were identified using FindVariableFeatures() function with 2000 variable features, followed by data scaling using ScaleData() and and dimensionality reduction using runPCA(). Harmony batch correction[57] across samples was performed using the RunHarmony(). UMAP embedding was computed from the Harmony-reduced dimensions using RunUMAP() on the first 30 dimensions. To define cell clusters, a shared nearest neighbor graph was computed using the FindNeighbors() function using the firest 30 dimensions, followed by clustering via FindClusters() with a resolution of 1.0. Resulting clusters were annotated based on canonical marker gene expression. The myeloid/granulocyte compartment was computationally isolated, and re-integrated as described above but with 500 variable features, and using the first 10 dimensions for the RunUMAP() and FindNeighbors() function, and FindClusters() resolution of 0.3. Myeloid/granulocyte celltypes were annotated according to canonical marker expression. A conventional dendritic cell cluster was computationally isolated, re-clustered using FindClusters() with a resolution of 0.2, and annotated as either cDC1 or cDC2 according to their canonical marker gene expression. Finally, doublet clusters displaying canonical markers of more than one celltype were removed, and a cluster of low-quality myeloid cells (relatively low library sizes with low number of genes detected).

Markers for the cell clusters 'cycling epithelial', 'fibroblasts', and macrophages ('mac MARCO' & 'mac FOLR2') were calculated from the integrated object using Seurat FindMarkers() with min.pct = 0.5, logfc.threshold = 0.25, after which the top 100 genes were ranked by average $\log_2$ fold-change, and the top 10 were retained. Marker genes included for macrophages: C1QB, C1QC, C1QA, SLCO2B1, MSR1, TREM2, VSIG4, CD163, SIGLEC1, APOC1. Fibroblast: SFRP4, WISP2, SFRP2, LUM, HTRA3, DPT, DCN, ASPN, CTSK, COL1A1. Cycling epithelial: CDC20, CCNB2, UBE2C, TOP2A, KIFC1, MKI67, NUSAP1, TPX2, CCNB1, BIRC5.

Differentially expressed genes from bulk RNA-seq analyses were grouped into gene sets and projected on the single cell landscape by calculating module scores for each gene set using the Seurat AddModuleScore() function, after which values were min–max scaled to the [0,1] interval.

For the MultiNicheNet analysis the nichenetr v2.2.1 and multinichenetr v2.1.0 packages in R were used. Only samples at baseline and week 2 and were retained for comparing baseline and on-treatment changes. Only samples at baseline with available transcriptomic risk classification were retained for comparing better versus worse outcome. The scRNA-seq Seurat object was transformed to a SingleCellExperiment object using the SingleCellExperiment package. NicheNet's ligand-receptor network and ligand-target matrix were downloaded from Zenodo (https://zenodo.org/record/10229222/files/lr_network_human_allInfo_30112033.rds and https://zenodo.org/record/7074291/files/ligand_target_matrix_nsga2r_final.rds). Celltypes with less than 10 cells were ignored after using the get_abundance_info() with min_cells = 10 to identify low abundance celltypes across conditions. We then filtered genes by minimum expression fraction using get_frac_exprs() using min_sample_prop = 0.50 and fraction_cutoff = 0.05. I.e., for each gene, we required nonzero counts in at least 5% of cells of a given cell type and expression in at least half of the samples for that cell type. Genes meeting these criteria in at least one cell type were retained for pseudobulk and differential expression analyses. Per-sample pseudobulk expression matrices were generated for each expressed gene in each retained cell type using process_abundance_expression_info(). Pseudobulk differential expression (DE) for each cell type and contrast was performed using get_DE_info(). The function combine_sender_receiver_de() produced a unified table of all possible sender–receiver pairs annotated with corresponding DE statistics. Ligand activities were evaluated in each receiver cell type with get_ligand_activities_targets_DEgenes() using logFC_threshold = 0.5 and pval_threshold = 0.05. Prioritization of communication patterns was performed with generate_prioritization_tables(), and ligand–receptor–target correlation analysis was computed using lr_target_prior_cor_inference(). All outputs were collected into a single list object, and a 'lite' output was made using make_lite_output() to facilitates downstream visualization. Finally, the top 50 ligand–receptor pairs by combined prioritization score were extracted using get_top_n_lr_pairs(). These interactions were visualized as side-by-side circos diagrams per condition using make_circos_group_comparison().

Whole-exome sequencing

Whole-exome sequencing (WES) data were processed using the Nextflow VEGAN pipeline (v2.1.1) (https://github.com/bioinfo-pf-curie/vegan). Sequencing reads were aligned to the human reference genome (hg38) with BWA-MEM, and only reads mapping to targeted exome regions with a minimum mapping quality score of 20 were retained. Duplicate reads were identified and removed using the Picard MarkDuplicates tool. Somatic variant calling was performed on matched tumor–normal pairs following the GATK best practices workflow, employing Mutect2. WES of paired blood samples were used as a reference. Variant annotation was conducted with snpEff. For downstream analyses, we included only somatic, coding, non-synonymous variants with a variant allele frequency (VAF) greater than 5% and a sequencing depth of at least 20×. Variants present as common polymorphisms in the gnomAD and 1000 Genomes Project databases were excluded. After variant filtering, 79 samples had at least one somatic coding mutation retained for downstream analyses.

Fisher's exact test (two-sided) was used to assess, for each driver gene, whether mutation status (mutation present or absent) differed between the better and worse prognosis groups. Univariate Cox proportional hazards regression was performed to evaluate the association between driver gene mutation status and progression-free survival (PFS). Hazard ratios (HRs) with 95% confidence intervals (CIs) are shown with p-values indicating two-sided Wald test.

Mutations in PI3K pathway genes (i.e., PIK3CA, PIK3R1, PIK3C2B, PIK3CG, PTEN, MTOR, RICTOR, AKT1) were analyzed as follows. Samples were classified as mutated if at least one gene in the pathway was altered. Fisher's exact test (two-sided) was used to compare the prevalence of PI3K pathway mutations between tumors with better and worse prognostic outcomes.

Tumor mutational burden (TMB) was calculated as part of the Vegan pipeline (see above). Cox proportional hazards regression was performed to evaluate the association between TMB and PFS. Hazard ratios (HRs) with 95% confidence intervals (CIs) are shown with *p*-values indicating two-sided Wald test.

Slide preparation

Slide preparation was centralized at IBBL and stored at −20C. Quarterly shipment on dry ice were organized toward the 2 central labs: CHUV (Lausanne, Switzerland) and UoZ (Zurich, Switzerland). All samples were stored short-term at −80C in the central labs until analysis. For samples that generated at least 10 slides of 3 microns, the FFPE block was serially cut with the 1st slide used for the first mIF panel (CHUV). Slides 2 was incubated with the IMC panel (UoZ) for deep characterization. The third slide was kept for an additional IMC panel not reported here. On slides 4 and 5, the last 2 mIF panels were performed (CHUV). For samples with limited tissue availability (e.g., biopsies), one slide was used to perform the mIF population panels and 2 slides for the IMC panels.

Spectral IF

7-plex IF panels were run using the multiplex Ventana autostainer (Roche) followed by multispectral imaging using the Vetra Polaris (PerkinElmer) as previously described.[29] The Following 3 panels were selected and validated for this project: mIF panel 1: CD11c, CD15, CD163, CD20, CD3, pan-Cytokeratin, DAPI; mIF panel 2: CD8, Granzyme-B, PD-1, PD-L1, Ki67, pan-Cytokeratin, DAPI; mIF panel 3: CD3, CD8, FoxP3, CD4, CD56, pan-Cytokeratin, DAPI. Briefly, staining comprised automated deparaffinization and antigen retrieval, endogenous peroxidase blocking, sequential primary-antibody incubation, detection with species-specific HRP-conjugated secondary reagents, Opal fluorophore deposition, and heat-mediated antibody removal between staining cycles. The mIF stained slides were scanned using the PhenoImager HT (Akoya Biosciences) with the MOTiF^{TM} mode, allowing whole slide multispectral image acquisition. The scanner outputs a multi-channel 8-bit image in QPTIFF format.

mIF cell segmentation and annotation is described previously.[29] Briefly, analysis is performed with the *IFQuant* software (Multimodal image analysis for large scale cancer tissue studies within IMMUcan developed for the IMMUcan project).[29] It provides an integrated web interface to facilitate manual analysis steps. It uses free-hand drawing tool to assign manual annotation masks to the images to either filter unwanted regions (e.g., out of focus, bad tissue quality) or label specific tissue regions (e.g., tumor, necrosis, healthy tissue, adipose tissue). Nuclear segmentation is performed automatically based on the DAPI signal and single cells are defined based on an expansion of the nuclear mask. Tumor islets segmentation is automatically done based on pan-Cytokeratin markers. Finally, *IFQuant* web interface is used to refine automatically set thresholds for each marker to extract positive and negative cells which can simultaneously be visually inspected for quality control. A CSV file is generated for each image containing a row for each cell with the summary intensity of each marker, positivity for each marker, the area of the cell, localization of the cell (tumor, stroma, or TLS), the region of interest (Tumor, next-to-tumor, necrosis, fat) and its X and Y coordinates. Next, cells are labeled based on phenotypes of marker expression as previously described.[29] 'BnT' are cells positive for CD20 and CD3. From these data, densities (cells per mm^2) for each cell type or phenotype are calculated.

Based on the density values at baseline, univariate Cox proportional hazards regression models were applied for survival analyses (PFS and OS) to estimate hazard ratios (HRs) with 95% confidence intervals (CIs). Cell types with zero density in more than 20% of samples were excluded, as well as CD8$^+$CD56$^+$ and CD8$^+$CD56$^+$FOXP3$^+$ populations because of their ambiguous phenotypes or potential marker spillover. In addition, univariate logistic regression models were fitted using the binary prognosis defined by the risk signature stratification, yielding odds ratios (ORs) with 95% Wald confidence intervals. All predictors were z-scored prior to modeling, so HRs and odds ratios (ORs) represent the relative change in risk or odds per one standard deviation increase in the corresponding cell-type density. Reported *p*-values correspond to two-sided Wald tests without correction for multiple comparisons.

Imaging mass cytometry (IMC)

IMC data was generated standardized within the IMMUcan project as described in detail.[29] Antibody conjugation was performed following the manufacturer's protocol using the MaxPar labeling kits (Standard Biotools). Conjugated antibodies were stored at 500 μm/mL in tris-based antibody stabilizing solution (Candor Biosciences) at 4°C. For staining, ready-to-use antibody mixes for IMC were prepared in in TBS with 3% BSA and 0.1% Tween, aliquoted and stored at −80°C.

This study only utilized IMC panel 1 from IMMUcan, comprising the following markers as described previously[29]: MPO, H3, SMA, CD16, CD38, HLA-DR, CD27, CD15, CD45RA, CD163, B2M, CD20, CD68, IDO1, CD3, LAG3, CD11c, PD1, PDGFRβ, CD7, Granzyme B (GB), PD-L1, TCF7, CD45RO, FOXP3, ICOS, CD8a, CAIX, CD33, Ki67, VISTA, CD40, CD4, CD14, E-Cadherin (ECad), CD303, CD206, Cleaved PARP, DNA1, DNA2. FFPE slides were processed in batches of 19 slides. Each batch was removed from −80°C, equilibrated to room temperature, deparaffinized, rehydrated and stored in TBS (pH 7.6). After blocking (TBS, 3% BSA, 0.1% Tween), antibody mix aliquots were thawed, supplemented with fluorescent-labeled antibodies for region selection (pan Cytokeratin, CD45 and CD163) and slides stained at 4°C over night. Slides were washed with TBS, nuclear staining performed with Iridium intercalator (1 μM) and Hoechst solution (2 μg/mL), washed again, dipped into ddH2O and dried under air flow.

Dried slides were scanned within 48 h after staining with a Zeiss AxioScan with 10× magnification and filters for DAPI, 488, 555 and 647 using the ZEN software. Subsequently, Panoramas were generated for each batch of slides using a robotic slides loader for Hyperion+ (Standard BioTools) with customized software (Eling et al., 2025). The czi files from the slide scanner and the Panoramas

(MCD files) were aligned using a custom napari plugin. After landmark-based alignment, three regions of interest (ROIs) - containing 30–70% of tumor cells together with immune and stromal cells - were selected for a total of ~1 mm^2. Additionally, if CD20 dense regions were visible – potentially tertiary lymphoid structures – a fourth ROI was selected. Acquisitions were performed on a Hyperion+ system in batch mode using a robotic slides loader with 400 Hz and 4 dB ablation energy.

MCD files from IMC were processed using the steinbock toolkit (v0.14.1)[53] First, TIFF files were generated and hot pixel filtered. Segmentation was performed using the Mesmer[58] implementation within steinbock. Histone H3 and Iridium were used for nuclei detection and E-cadherin, CD3, CD8, CD20, CD163, were used as markers for cellular boundaries. Cellular interactions were quantified as cells that touch after a cell boundary expansion of 4 μm. Finally, the mean marker intensities, cellular area, centroid, major and minor axis length and eccentricity were calculated per cell. One CSV file per image containing the mean pixel intensities, one CSV per image containing the cellular information and one CSV per image containing the neighboring cell information were exported.

IMC downstream processing

Cell typing was performed as described in Eling et al., 2025. Briefly, manually gated cells were used to train a random forest classifier. The pre-trained classifier was used to predict cell types based on marker expression. Cells with a maximum probability of less than 30% for any cell type were labeled as "unknown". Cells co-expressing CD3 and CD20 were classified as 'BnT' cells, representing B cells that sit in close spatial proximity to T cells. HLA-DR^+ cells lacking canonical lineage markers (CD20, CD11c, CD163) were grouped into an $HLADR^+$ category to capture unresolved antigen-presenting cell populations. Classified cell types were manually quality controlled. For this, images of each cell type were prepared outlining the mask on top of relevant markers for the respective cell type. Warning messages were generated when the classification appeared incorrect. Extra images acquired on TLS – if present – were excluded from the primary analysis. Furthermore, images containing less than 10% tumor cells or less than 500 cells were excluded from the analysis. Cell type densities per patient were calculated by summing up individual cell type counts across all images and dividing them by the total acquired area of that patient in mm^2.

For IMC-derived cell densities (cells per mm^2) at baseline, univariate Cox proportional hazards models were applied for PFS and OS to estimate HRs with 95% confidence intervals. Univariate logistic regression models were fitted using the binary prognosis defined by risk-signature stratification, yielding ORs with 95% confidence intervals. All predictors were z-scored prior to modeling, so HRs and ORs represent the change in risk or odds per one standard deviation increase in the corresponding cell-type density. Reported *p*-values correspond to two-sided Wald tests without correction for multiple comparisons.

Cellohood

We trained a standard Cellohood model on all IMC samples from SYNERGY, using cell bags with a radius of 25 μm. This model yielded cell-level, neighborhood-enriched representations, which we clustered into 27 groups. For each cluster, we calculated the proportion of cell types assigned to it, as well as the average marker expression per cell type (restricted to clusters in which the given cell type exceeded 5% of the composition).

For the differential analyses (volcano plots) comparing better versus worse prognosis, and week 2 versus baseline, we used log1p-transformed counts. Clusters were considered differential if the absolute log2 fold change exceeded 2 and the false discovery rate (FDR; Benjamini-Hochberg correction) adjusted q-value (derived from a two-sided *t* test) was below 0.01.

Immunohistochemistry

Immunohistochemistry assessment of PD-L1 (VENTANA SP263 assay) and CD73 (Abcam EPR6115 clone, ab124725, 1:250 dilution) was described previously.[12]

TILs scoring

Tumor-infiltrating lymphocytes (TILs) were assessed on H&E slides from the SYNERGY trial as previously described.[12]

QUANTIFICATION AND STATISTICAL ANALYSIS

All analyses and visualizations were performed using R statistical computing software v4.4.1. Descriptions of each statistical test are given in the figure legends and relevant Method details. FDR indicates Benjamini-Hochberg corrected *p*-values. Sample sizes are indicated by (n =). Cox regression and logistic regression results are reported as point estimates (hazard ratio [HR] or odds ratio [OR]) with 95% confidence intervals, shown as whiskers in forest-style plots. Boxplots show the median (center line) and interquartile range (IQR, box), with whiskers extending to 1.5× the IQR. In the module score violin plots, red dashed lines indicate the 10th and 90th percentiles of all module scores, and yellow lines indicate median of celltype module scores.

ADDITIONAL RESOURCES

The SYNERGY trial was registered on ClinicalTrials.gov (https://clinicaltrials.gov/) under ID: NCT03616886. The EORTC-1553-SPECTA initiative under ID: NCT02834884. Wilkerson et al. under ID: NCT03121352. TBCRC-043 under ID: NCT03206203. TONIC under ID: NCT02499367. AURORA under ID: NCT02102165 and EORTC ID: EORTC-1408-BCG.

Cell Reports Medicine, Volume *7*

Supplemental information

A multi-omics analysis of advanced triple-negative breast cancer identifies predictors to chemo-immunotherapy and on-treatment dynamics

Nicolaas Van Renne, Elisa Agostinetto, Andrea Joaquín García, Marie Morfouace, Marcin Możejko, Zoë Denis, Sophie Déglise, Bernd Bodenmiller, Henoch S. Hong, David Venet, Marcela Carausu, Robin Liechti, Stéphanie Tissot, Sylvie Rusakiewicz, Mattia Rediti, Xavier Catteau, Daniel Schulz, Delphine Loirat, Daniel Herrero Saboya, Philippe Aftimos, Matteo Benelli, Angel L. Guerrero-Zotano, Andrea Gombos, Hans Wildiers, Jip Van Dinter, Ariane Sickinghe, Nicolas Penel, Jean-Charles Goeminne, Júlio Oliveira, Birute Brasiuniene, Emanuela Romano, Justin M. Balko, Marleen Kok, Ewa Szczurek, Christos Sotiriou, and Laurence Buisseret

Supplemental Information

A multi-omics analysis of advanced Triple-Negative Breast Cancer identifies predictors to chemo-immunotherapy and on-treatment dynamics

Nicolaas Van Renne, Elisa Agostinetto, Andrea Joaquín García, Marie Morfouace, Marcin Możejko, Zoë Denis, Sophie Déglise, Bernd Bodenmiller, Henoch S Hong, David Venet, Marcela Carausu, Robin Liechti, Stéphanie Tissot, Sylvie Rusakiewicz, Mattia Rediti, Xavier Catteau, Daniel Schulz, Delphine Loirat, Daniel Herrero Saboya, Philippe Aftimos, Matteo Benelli, Angel L Guerrero-Zotano, Andrea Gombos, Hans Wildiers, Jip Van Dinter, Ariane Sickinghe, Nicolas Penel, Jean-Charles Goeminne, Júlio Oliveira, Birute Brasiuniene, Emanuela Romano, Justin Balko, Marleen Kok, Ewa Szczurek, Christos Sotiriou and Laurence Buisseret.

Figures S1-S6

Figure S1. Extended characterization of the transcriptomic risk signature in the SYNERGY cohort, related to figures 2 and 3.

Figure S2. Performance of the transcriptomic risk signature across independent metastatic TNBC cohorts, related to figures 2.

Figure S3. TNBC molecular subtypes and clinical outcome across 1st line metastatic TNBC cohorts, related to figure 2.

Figure S4. On-treatment differentially expressed genes from RNA-seq projected on scRNA-seq, related to figure 5.

Figure S5: scRNA-seq landscape of SYNERGY, related to figure 5.

Figure S6: mIF and IMC density analysis, related to figure 6.

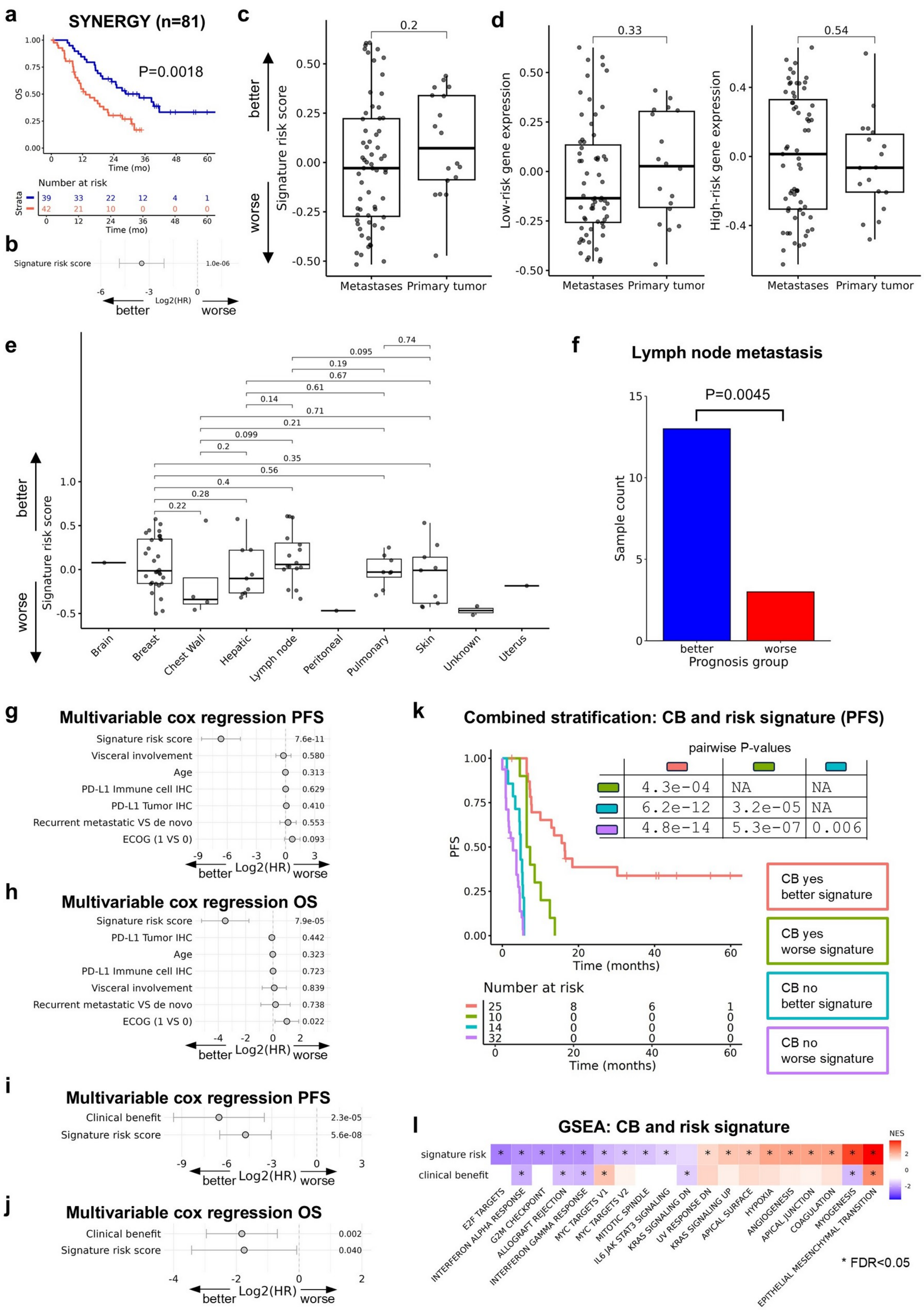
a
SYNERGY (n=81)
P=0.0018
OS
Time (mo)
Number at risk
Strata
39 33 22 12 4 1
42 21 10 0 0 0
b
Signature risk score
1.0e-06
Log2(HR)
better
worse
c
Signature risk score
better
worse
Metastases
Primary tumor
d
Low-risk gene expression
High-risk gene expression
e
Brain
Breast
Chest Wall
Hepatic
Lymph node
Peritoneal
Pulmonary
Skin
Unknown
Uterus
f
Lymph node metastasis
P=0.0045
Sample count
Prognosis group
g
Multivariable cox regression PFS
Signature risk score 7.6e-11
Visceral involvement 0.580
Age 0.313
PD-L1 Immune cell IHC 0.629
PD-L1 Tumor IHC 0.410
Recurrent metastatic VS de novo 0.553
ECOG (1 VS 0) 0.093
h
Multivariable cox regression OS
Signature risk score 7.9e-05
PD-L1 Tumor IHC 0.442
Age 0.323
PD-L1 Immune cell IHC 0.723
Visceral involvement 0.839
Recurrent metastatic VS de novo 0.738
ECOG (1 VS 0) 0.022
i
Multivariable cox regression PFS
Clinical benefit 2.3e-05
Signature risk score 5.6e-08
j
Multivariable cox regression OS
Clinical benefit 0.002
Signature risk score 0.040
k
Combined stratification: CB and risk signature (PFS)
pairwise P-values
4.3e-04 NA NA
6.2e-12 3.2e-05 NA
4.8e-14 5.3e-07 0.006
PFS
Time (months)
Number at risk
25 8 6 1
10 0 0 0
14 0 0 0
32 0 0 0
CB yes better signature
CB yes worse signature
CB no better signature
CB no worse signature
l
GSEA: CB and risk signature
signature risk
clinical benefit
NES
E2F TARGETS
INTERFERON ALPHA RESPONSE
G2M CHECKPOINT
ALLOGRAFT REJECTION
INTERFERON GAMMA RESPONSE
MYC TARGETS V1
MYC TARGETS V2
MITOTIC SPINDLE
IL6 JAK STAT3 SIGNALING
KRAS SIGNALING DN
UV RESPONSE DN
KRAS SIGNALING UP
APICAL SURFACE
HYPOXIA
ANGIOGENESIS
APICAL JUNCTION
COAGULATION
MYOGENESIS
EPITHELIAL MESENCHYMAL TRANSITION
* FDR<0.05

Figure S1: Extended characterization of the transcriptomic risk signature in the SYNERGY cohort. (a) Kaplan-Meier curves for OS stratified by the transcriptomic risk signature and **(b)** univariable Cox regression analysis showing the association between the continuous signature risk score and OS. **(c)** Signature risk scores and **(d)** GSVA-derived enrichment scores of the high- and low-risk gene sets in primary tumor versus metastatic biopsy samples. P-values indicate two-sided Wilcoxon rank-sum test. **(e)** Distribution of signature risk scores across individual metastatic biopsy sites. P-values indicate two-sided Wilcoxon rank-sum test. **(f)** Distribution of prognosis groups within lymph node metastases. P-value indicates two-sided Fisher's exact test. **(g-h)** Forest plots from multivariable Cox proportional hazards regression analysis including the signature risk score and key clinicopathological variables for PFS and OS. **(i-j)** Multivariable Cox regression including CB status and the signature risk stratification for PFS. **(k)** Kaplan-Meier curves showing joint stratification by CB status and transcriptomic risk signature. **(l)** GSEA showing hallmark pathway enrichment of tumors stratified by the transcriptomic risk signature and CB status. P-values for Kaplan-Meier curves were determined using the two-sided log-rank test. Cox regression P-values indicate two-sided Wald test.

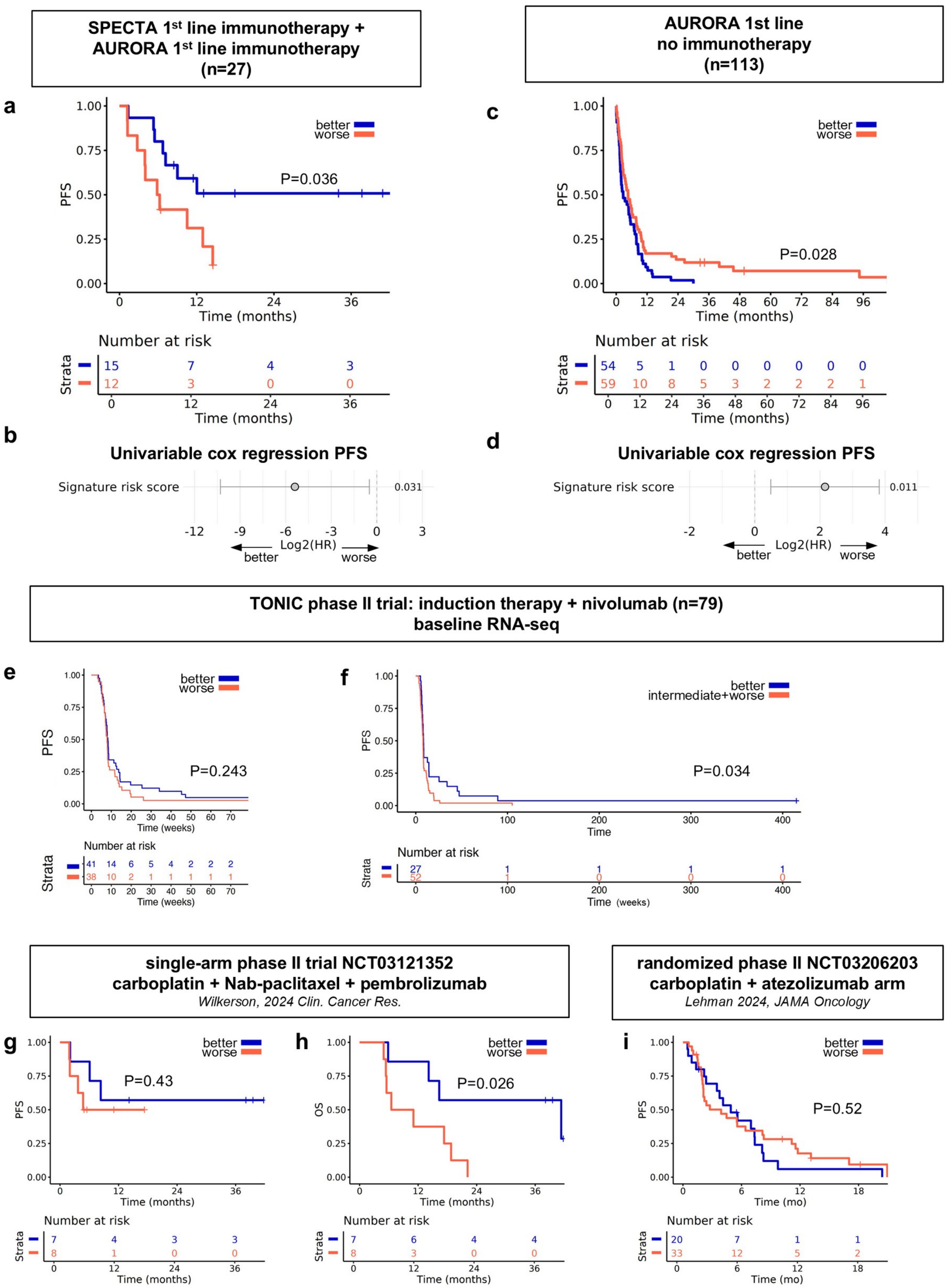

SPECTA 1st line immunotherapy + AURORA 1st line immunotherapy (n=27)
a
PFS
better
worse
P=0.036
Time (months)
Number at risk
Strata
15 7 4 3
12 3 0 0
b
Univariable cox regression PFS
Signature risk score
0.031
Log2(HR)
better
worse
AURORA 1st line no immunotherapy (n=113)
c
PFS
better
worse
P=0.028
Time (months)
Number at risk
Strata
54 5 1 0 0 0 0 0 0
59 10 8 5 3 2 2 2 1
d
Univariable cox regression PFS
Signature risk score
0.011
Log2(HR)
better
worse
TONIC phase II trial: induction therapy + nivolumab (n=79)
baseline RNA-seq
e
PFS
better
worse
P=0.243
Time (weeks)
Number at risk
Strata
41 14 6 5 4 2 2 2
38 10 2 1 1 1 1 1
f
PFS
better
intermediate+worse
P=0.034
Time
Number at risk
Strata
27 1 1 1 1
52 1 0 0 0
Time (weeks)
single-arm phase II trial NCT03121352
carboplatin + Nab-paclitaxel + pembrolizumab
Wilkerson, 2024 Clin. Cancer Res.
randomized phase II NCT03206203
carboplatin + atezolizumab arm
Lehman 2024, JAMA Oncology
g
PFS
better
worse
P=0.43
Time (months)
Number at risk
Strata
7 4 3 3
8 1 0 0
h
OS
better
worse
P=0.026
Time (months)
Number at risk
Strata
7 6 4 4
8 3 0 0
i
PFS
better
worse
P=0.52
Time (mo)
Number at risk
Strata
20 7 1 1
33 12 5 2

Figure S2: Performance of the transcriptomic risk signature across independent metastatic TNBC cohorts. (a) Kaplan-Meier curves for PFS stratified by the transcriptomic risk signature and **(b)** univariable Cox regression analysis in the combined SPECTA and AURORA first-line chemo-ICI cohort (n=27). **(c-d)** Similarly, for AURORA patients treated with first-line systemic therapy without immunotherapy (n=113). **(e)** Kaplan-Meier curves for PFS stratified by the better-versus-worse transcriptomic risk classification in the TONIC phase II trial (n=79, Kaplan Meier curves truncated at 80 weeks of follow-up) and **(f)** by using a three-group stratification, where samples with low classification confidence (permutation test P>0.05) were assigned to an intermediate group; intermediate and worse-risk groups were combined for comparison. **(g)** Kaplan-Meier curves for PFS and **(h)** OS in the Wilkerson phase II trial (NCT03121352) and **(i)** for PFS in the TBCRC-043 phase II trial (NCT03206203). P-values for Kaplan-Meier curves indicate two-sided log-rank test. Cox regression P-values indicate two-sided Wald test.

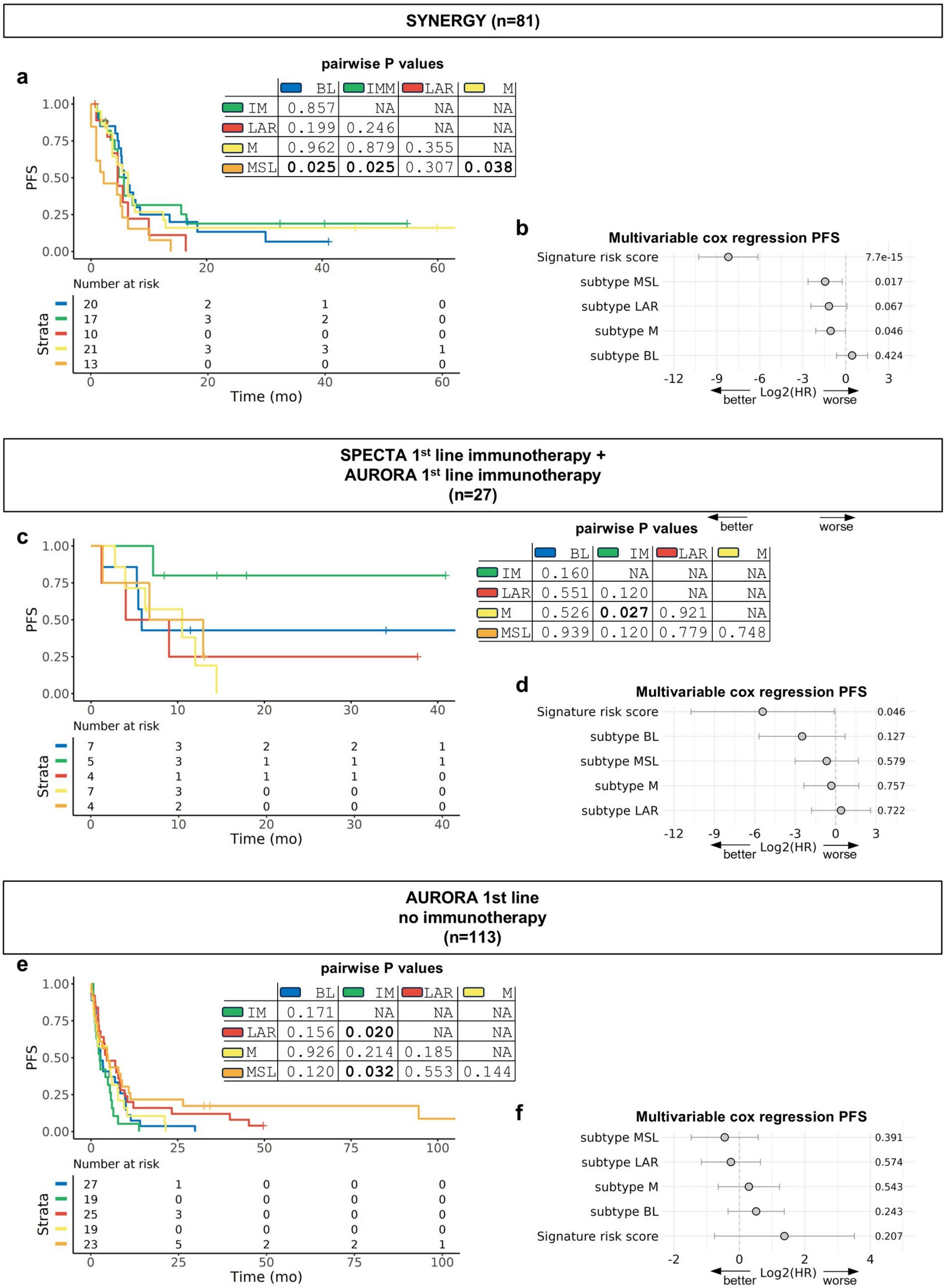
SYNERGY (n=81)
a
pairwise P values
BL IMM LAR M
IM 0.857 NA NA NA
LAR 0.199 0.246 NA NA
M 0.962 0.879 0.355 NA
MSL 0.025 0.025 0.307 0.038
PFS
1.00 0.75 0.50 0.25 0.00
0 20 40 60
Number at risk
Strata
20 2 1 0
17 3 2 0
10 0 0 0
21 3 3 1
13 0 0 0
0 20 40 60
Time (mo)
b
Multivariable cox regression PFS
Signature risk score 7.7e-15
subtype MSL 0.017
subtype LAR 0.067
subtype M 0.046
subtype BL 0.424
-12 -9 -6 -3 0 3
better Log2(HR) worse
SPECTA 1st line immunotherapy +
AURORA 1st line immunotherapy
(n=27)
c
pairwise P values
better worse
BL IM LAR M
IM 0.160 NA NA NA
LAR 0.551 0.120 NA NA
M 0.526 0.027 0.921 NA
MSL 0.939 0.120 0.779 0.748
PFS
1.00 0.75 0.50 0.25 0.00
0 10 20 30 40
Number at risk
Strata
7 3 2 2 1
5 3 1 1 1
4 1 1 1 0
7 3 0 0 0
4 2 0 0 0
0 10 20 30 40
Time (mo)
d
Multivariable cox regression PFS
Signature risk score 0.046
subtype BL 0.127
subtype MSL 0.579
subtype M 0.757
subtype LAR 0.722
-12 -9 -6 -3 0 3
better Log2(HR) worse
AURORA 1st line
no immunotherapy
(n=113)
e
pairwise P values
BL IM LAR M
IM 0.171 NA NA NA
LAR 0.156 0.020 NA NA
M 0.926 0.214 0.185 NA
MSL 0.120 0.032 0.553 0.144
PFS
1.00 0.75 0.50 0.25 0.00
0 25 50 75 100
Number at risk
Strata
27 1 0 0 0
19 0 0 0 0
25 3 0 0 0
19 0 0 0 0
23 5 2 2 1
0 25 50 75 100
Time (mo)
f
Multivariable cox regression PFS
subtype MSL 0.391
subtype LAR 0.574
subtype M 0.543
subtype BL 0.243
Signature risk score 0.207
-2 0 2 4
better Log2(HR) worse

Figure S3: TNBC molecular subtypes and clinical outcome across 1st line metastatic TNBC cohorts. (a) Kaplan-Meier curves for PFS across molecular subtypes in the SYNERGY cohort. **(b)** Forest plot from multivariable Cox regression including molecular subtype (IM as reference) and the transcriptomic risk score for PFS in SYNERGY. **(c-d)** Similarly for the combined SPECTA and AURORA first-line chemo-ICI cohort (n=27) and **(e-f)** the AURORA patients treated with first-line systemic therapy without immunotherapy (n=113). P-values for Kaplan-Meier curves were determined using the two-sided log-rank test. Cox regression P-values indicate two-sided Wald test.

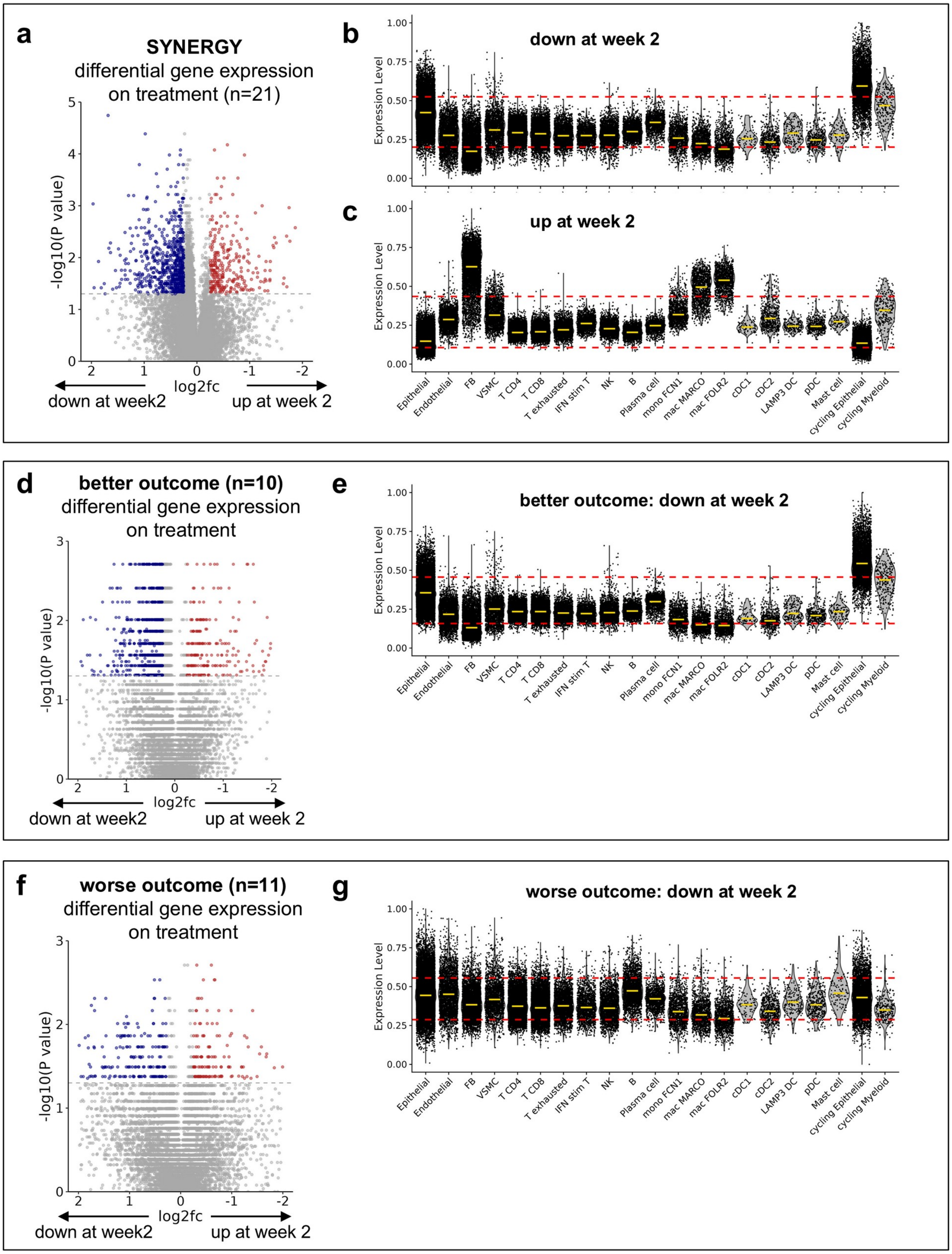
a
SYNERGY
differential gene expression
on treatment (n=21)
-log10(P value)
log2fc
down at week2
up at week 2
b
down at week 2
Expression Level
c
up at week 2
Expression Level
Epithelial
Endothelial
FB
VSMC
T CD4
T CD8
T exhausted
IFN stim T
NK
B
Plasma cell
mono FCN1
mac MARCO
mac FOLR2
cDC1
cDC2
LAMP3 DC
pDC
Mast cell
cycling Epithelial
cycling Myeloid
d
better outcome (n=10)
differential gene expression
on treatment
-log10(P value)
log2fc
down at week2
up at week 2
e
better outcome: down at week 2
Expression Level
f
worse outcome (n=11)
differential gene expression
on treatment
-log10(P value)
log2fc
down at week2
up at week 2
g
worse outcome: down at week 2
Expression Level

Figure S4: On-treatment differentially expressed genes from RNA-seq projected on scRNA-seq. (a) Volcano plot of differentially expressed genes showing bulk RNA-seq genes significantly upregulated (red) and downregulated (blue) on treatment (n=21). Dashed lines indicate 10th and 90th percentile of all module scores. **(b-c)** Scaled module scores of upregulated and downregulated genes are projected on the SYNERGY scRNA-seq landscape. **(d-e)** Similar volcano plots and single-cell projections were generated for the subset of patients with a better prognosis (n=10) and **(f-g)** a worse prognosis (n=11). Volcano plot P values were calculated by two-sided Wilcoxon signed-rank test. Fc: fold change.

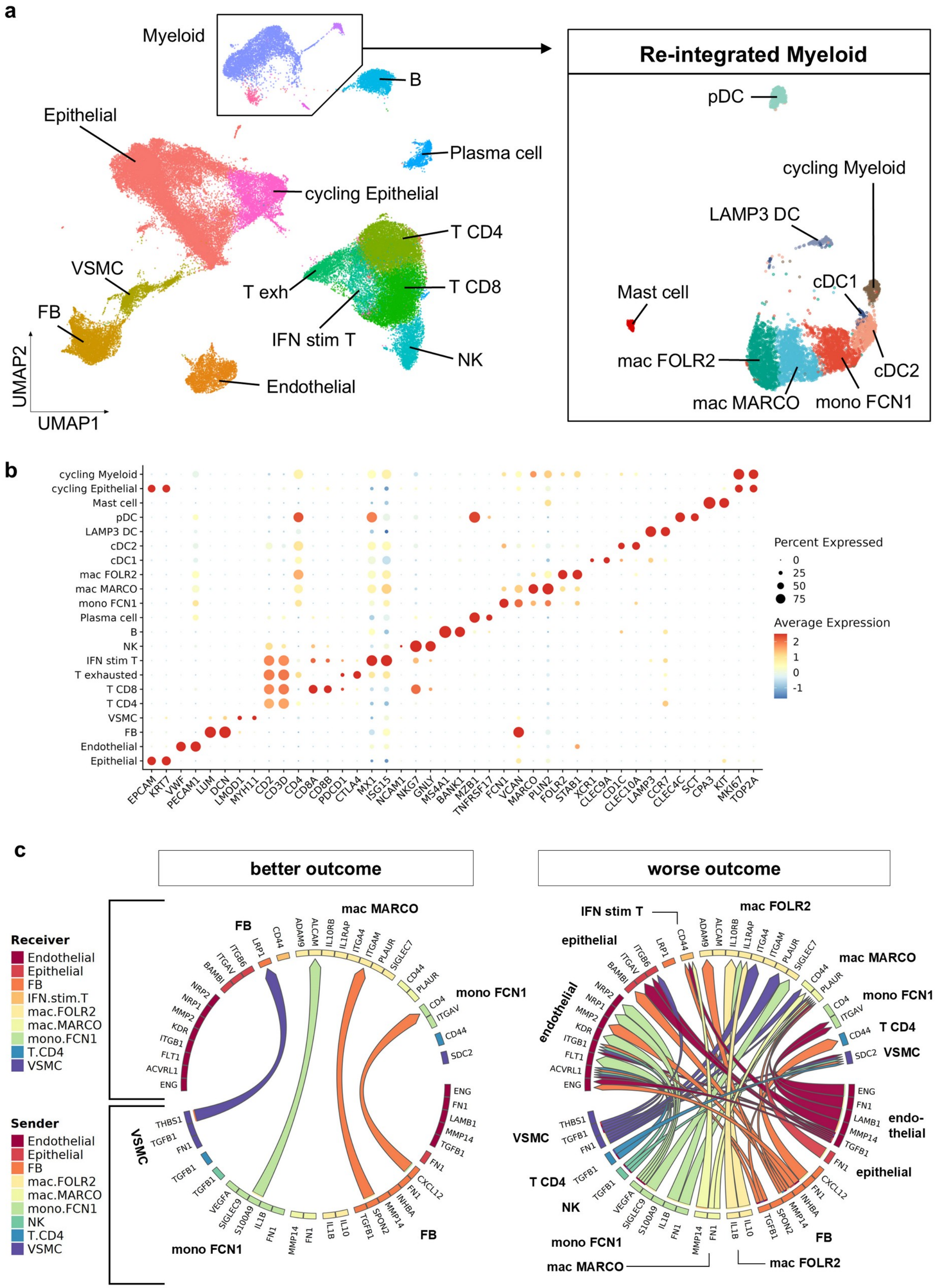

a
Myeloid
B
Epithelial
Plasma cell
cycling Epithelial
T CD4
VSMC
T exh
T CD8
FB
IFN stim T
NK
Endothelial
UMAP2
UMAP1
Re-integrated Myeloid
pDC
cycling Myeloid
LAMP3 DC
cDC1
Mast cell
mac FOLR2
cDC2
mac MARCO
mono FCN1
b
cycling Myeloid
cycling Epithelial
Mast cell
pDC
LAMP3 DC
cDC2
cDC1
mac FOLR2
mac MARCO
mono FCN1
Plasma cell
B
NK
IFN stim T
T exhausted
T CD8
T CD4
VSMC
FB
Endothelial
Epithelial
EPCAM KRT7 VWF PECAM1 LUM DCN LMOD1 MYH11 CD2 CD3D CD4 CD8A CD8B PDCD1 CTLA4 MX1 ISG15 NCAM1 NKG7 GNLY MS4A1 BANK1 MZB1 TNFRSF17 FCN1 VCAN MARCO PLIN2 FOLR2 STAB1 XCR1 CLEC9A CD1C CLEC10A LAMP3 CCR7 CLEC4C SCT CPA3 KIT MKI67 TOP2A
Percent Expressed
0
25
50
75
Average Expression
2
1
0
-1
c
better outcome
worse outcome
Receiver
Endothelial
Epithelial
FB
IFN.stim.T
mac.FOLR2
mac.MARCO
mono.FCN1
T.CD4
VSMC
Sender
Endothelial
Epithelial
FB
mac.FOLR2
mac.MARCO
mono.FCN1
NK
T.CD4
VSMC
FB
mac MARCO
mono FCN1
VSMC
mono FCN1
FB
IFN stim T
mac FOLR2
epithelial
mac MARCO
mono FCN1
endothelial
T CD4
VSMC
endo-thelial
epithelial
VSMC
T CD4
NK
mono FCN1
FB
mac MARCO
mac FOLR2

Figure S5: scRNA-seq landscape of SYNERGY. (a) UMAP dimensionality reduction plot with annotated clusters, with the inset showing re-integrated myeloid and granulocyte compartment. **(b)** Dot plots display marker gene expression for each cluster. Dot size indicates the proportions of expressing cells, colored by standardized expression levels. **(c)** MultiNicheNet analysis of cell-cell communication at baseline in better versus worse prognosis patients. Circos plots shows the top 50 differential ligand-receptor interactions between cell types in patients with better outcome (left) and worse outcome (right) at baseline. Arrows indicate directionality from sender to receiver cell type, and arrow color denotes sender cell type. Mac: macrophage. Mono: monocyte. cDC and pDC: conventional and plasmacytoid dendritic cell. IFN stim T: interferon stimulated T cell. Texh: exhausted T cell. VSMC: vascular smooth cell. FB: fibroblast.

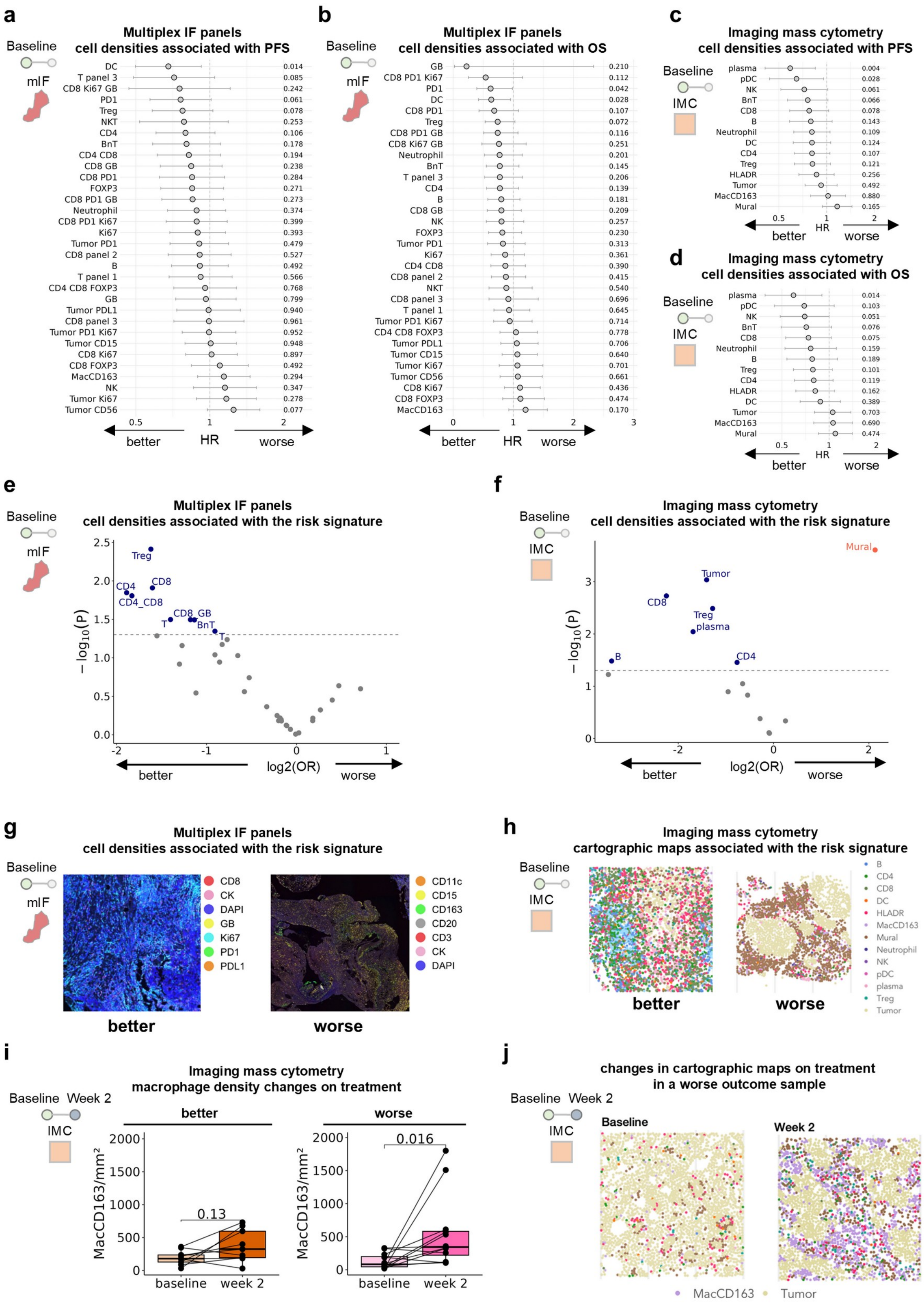
a
Baseline
mIF
Multiplex IF panels
cell densities associated with PFS
DC 0.014
T panel 3 0.085
CD8 Ki67 GB 0.242
PD1 0.061
Treg 0.078
NKT 0.253
CD4 0.106
BnT 0.178
CD4 CD8 0.194
CD8 GB 0.238
CD8 PD1 0.284
FOXP3 0.271
CD8 PD1 GB 0.273
Neutrophil 0.374
CD8 PD1 Ki67 0.399
Ki67 0.393
Tumor PD1 0.479
CD8 panel 2 0.527
B 0.492
T panel 1 0.566
CD4 CD8 FOXP3 0.768
GB 0.799
Tumor PDL1 0.940
CD8 panel 3 0.961
Tumor PD1 Ki67 0.952
Tumor CD15 0.948
CD8 Ki67 0.897
CD8 FOXP3 0.492
MacCD163 0.294
NK 0.347
Tumor Ki67 0.278
Tumor CD56 0.077
0.5
1
2
better
HR
worse
b
Baseline
mIF
Multiplex IF panels
cell densities associated with OS
GB 0.210
CD8 PD1 Ki67 0.112
PD1 0.042
DC 0.028
CD8 PD1 0.107
Treg 0.072
CD8 PD1 GB 0.116
CD8 Ki67 GB 0.251
Neutrophil 0.201
BnT 0.145
T panel 3 0.206
CD4 0.139
B 0.181
CD8 GB 0.209
NK 0.257
FOXP3 0.230
Tumor PD1 0.313
Ki67 0.361
CD4 CD8 0.390
CD8 panel 2 0.415
NKT 0.540
CD8 panel 3 0.696
T panel 1 0.645
Tumor PD1 Ki67 0.714
CD4 CD8 FOXP3 0.778
Tumor PDL1 0.706
Tumor CD15 0.640
Tumor Ki67 0.701
Tumor CD56 0.661
CD8 Ki67 0.436
CD8 FOXP3 0.474
MacCD163 0.170
0
1
2
3
better
HR
worse
c
Baseline
IMC
Imaging mass cytometry
cell densities associated with PFS
plasma 0.004
pDC 0.028
NK 0.061
BnT 0.066
CD8 0.078
B 0.143
Neutrophil 0.109
DC 0.124
CD4 0.107
Treg 0.121
HLADR 0.256
Tumor 0.492
MacCD163 0.880
Mural 0.165
0.5
1
2
better
HR
worse
d
Baseline
IMC
Imaging mass cytometry
cell densities associated with OS
plasma 0.014
pDC 0.103
NK 0.051
BnT 0.076
CD8 0.075
Neutrophil 0.159
B 0.189
Treg 0.101
CD4 0.119
HLADR 0.162
DC 0.389
Tumor 0.703
MacCD163 0.690
Mural 0.474
0.5
1
2
better
HR
worse
e
Baseline
mIF
Multiplex IF panels
cell densities associated with the risk signature
Treg
CD8
CD4
CD4_CD8
CD8_GB
T
BnT
T
− log10(P)
log2(OR)
better
worse
f
Baseline
IMC
Imaging mass cytometry
cell densities associated with the risk signature
Mural
Tumor
CD8
Treg
plasma
B
CD4
− log10(P)
log2(OR)
better
worse
g
Baseline
mIF
Multiplex IF panels
cell densities associated with the risk signature
CD8
CK
DAPI
GB
Ki67
PD1
PDL1
CD11c
CD15
CD163
CD20
CD3
CK
DAPI
better
worse
h
Baseline
IMC
Imaging mass cytometry
cartographic maps associated with the risk signature
B
CD4
CD8
DC
HLADR
MacCD163
Mural
Neutrophil
NK
pDC
plasma
Treg
Tumor
better
worse
i
Baseline Week 2
IMC
Imaging mass cytometry
macrophage density changes on treatment
better
worse
0.13
0.016
MacCD163/mm²
baseline
week 2
j
changes in cartographic maps on treatment
in a worse outcome sample
Baseline Week 2
IMC
Baseline
Week 2
MacCD163
Tumor

Figure S6: mIF and IMC density analysis (a-b) Cox proportional hazards regression analyses for overall survival (OS) and progression-free survival (PFS) across mIF-derived and **(c-d)** IMC-derived cell-type densities. HR: hazard ratio; whiskers show 95% confidence interval. P-values indicate two-sided Wald test. **(e)** Volcano plots showing associations between mIF- and **(f)** IMC-derived cell-type densities and patient outcome according to the risk signature. The x-axis represents the $\log_2$ odds ratio (OR) from logistic regression models for each cell type's association with worse outcome (positive values) or better outcome (negative values) patients. P values indicate two-sided Wald test. GB: granzyme B^+ T cell, pDC plasmacytoid dendritic cell, BnT: B or T cell in close proximity, HLADR: undefined HLA-DR^+ antigen presenting cell, Plasma: plasma cell. **(g)** Example miF panels and **(h)** IMC cartographic maps of patients with a differential clinical outcome. **(i)** Boxplots showing differences in macrophage levels between baseline and week 2 on-treatment. P values indicate Wilcoxon signed-rank test. **(j)** Representative worse outcome sample at baseline and week 2, illustrating increased macrophage levels on-treatment.
